# Poly(acrylamido) PEG-Alternative Lipids Rescue mRNA Efficacy during Repeated Dosing and Enhance Immune Cell Delivery

**DOI:** 10.1021/acsnano.6c11940

**Published:** 2026-10-22

**Authors:** Benjamin M. Fiedler, Charlotte Galley, Margarita Strimaite, Nga Man Cheng, Najet Mahmoudi, Zhiping Feng, Tammy L. Kalber, Maria-Jose Martinez-Bravo, Christopher J. Morris, Jenny K. W. Lam, Daniel J. Stuckey, Gareth R. Williams, Clare L. Bennett, Pratik Gurnani

**Affiliations:** † UCL School of Pharmacy, 6123 University College London, London WC1N 1AX, U.K.; ‡ UCL Cancer Institute, Department of Haematology, 4919 University College London, London WC1E 6DD, U.K.; § UCL Centre for Advanced Biomedical Imaging, 4919 University College London, London WC1E 6DD, U.K.; ∥ School of Pharmacy, University of Nottingham to Division of Molecular Therapeutics and Formulation, Boots Science Building, School of Pharmacy, University of Nottingham, Nottingham NG7 2RD, U.K.; ⊥ ISIS Neutron and Muon Source, 15585 Rutherford Appleton Laboratory, Didcot OX11 0DE, U.K.

**Keywords:** lipid nanoparticle, mRNA delivery, PEG alternative, anti-PEG antibody, accelerated blood clearance, poly(acrylamido) lipid, dendritic cell

## Abstract

Following the successes of the messenger RNA (mRNA) lipid nanoparticle (LNP) vaccines against COVID-19, mRNA-LNPs are being explored for varied multidose applications including cancer immunotherapies and protein replacement therapies. LNPs rely on poly­(ethylene glycol)-lipid (PEG-lipid) coatings for stability, but increasing anti-PEG antibody prevalence threatens repeat-dose efficacy and increases pseudoallergic risk, creating an urgent need for alternatives. Here, we introduce poly­(acrylamido) lipids (PAM-lipids), a family of polymer lipids that effectively replace PEG-lipids in mRNA-LNP formulations. Systematic variation of monomer chemistry, molar mass, and end group reveals critical structure–activity relationships determining LNP size, polydispersity, and transfection efficiency. Varying PAM monomer chemistry allows minimization of antipolymer antibody cross-reactivity, with multiple PAM-lipids demonstrating no anti-PEG antibody binding. Several PAM-LNPs exhibit superior mRNA transfection efficacy to PEGylated clinical benchmarks *in vitro* and *in vivo* across multiple cell types, organs, routes of administration, and mRNA cargos. In particular, lead PAM-LNPs enhance mRNA delivery to dendritic cells (up to 120-fold *in vitro* and 3-fold *in vivo*) and macrophages (4-fold *in vivo*), with 2.5-fold superior lymph node expression, driven in part by increased cellular association and lymphoid targeting. Crucially, PAM-LNPs evade circulating anti-PEG antibodies to rescue lost mRNA efficacy after repeated dosing, resulting in 300% mRNA expression relative to that of PEG-LNPs. These findings demonstrate PAM-lipids as a chemically varied, immunologically compatible family of PEG alternatives for mRNA-LNP therapeutics.

## Graphical abstract

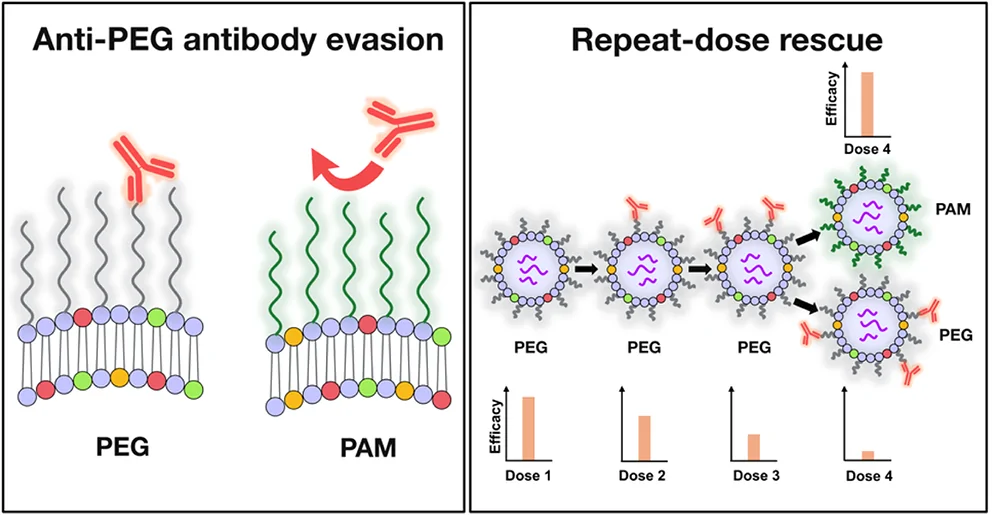

## Introduction

With the successful rollout of mRNA vaccines against COVID-19 (Comirnaty, Spikevax, Zapomeran, and Gemcovac), there has been increased development of mRNA lipid nanoparticle (LNP) technologies for a vast array of critical indications including cancer, cardiovascular disease, and genetic disorders.^1–3^ Currently, there are over 150 clinical trials for LNP-mediated gene delivery.^2^ LNPs are a crucial part of this technology as they protect RNA from degradation by nucleases and enhance cellular uptake/trafficking. Conventional LNPs are composed of four different components: an ionizable (cationic) lipid, cholesterol, a helper phospholipid, and a polymer–lipid conjugate. This multicomponent formulation allows control over the physical properties of the LNP, which in turn impact biological activity such as mRNA transfection efficiency, cell targeting, and tissue tropism. All clinically approved LNPs, and most under investigation, utilize poly­(ethylene glycol) (PEG)-lipids within their formulation to control particle size, aid storage stability, increase circulatory half-life, and reduce protein adsorption to the LNP surface.^1^ However, the ubiquity of PEG in other drug and cosmetic formulations meant that ∼72% of human blood samples tested positive for anti-PEG antibodies before the COVID-19 pandemic (2016).^4^ With antibody titers increasing up to 69-fold after the use of PEG in immunogenic COVID-19 vaccines.^5^ These pre-existing anti-PEG antibodies reduce the efficacy of future doses of PEGylated therapies in humans; a problem that is exacerbated during repeated dosing regimens.^6^ This is partly through the accelerated blood clearance effect (ABC), where opsonization by pre-existing anti-PEG antibodies increases clearance of LNPs by liver-resident macrophages (Kupffer cells).^7–10^ In addition to impacting intracellular trafficking (e.g., cellular uptake), opsonization can destabilize LNPs causing premature cargo leakage.^8,11,12^ By inducing structural instability and promoting immunogenicity, anti-PEG antibodies directly counteract the intended stealth and stabilizing properties of PEGylation in LNP formulations. It is therefore likely that future mRNA therapies will exhibit poor treatment outcomes in patients with pre-existing anti-PEG antibodies, for example, after COVID-19 mRNA-LNP vaccination, if PEGylated LNPs (PEG-LNPs) are used. It has been previously hypothesized, but not demonstrated, that it may be possible to recover this lost efficacy by dosing LNPs with a PEG-alternative surface, evading opsonization from anti-PEG antibodies since these are unable to bind to PEG alternatives with different chemical structures.^13^ Furthermore, sensitization to PEG has been associated with adverse pseudoallergic responses (complement-activation-related pseudoallergy, CARPA), with these events rare but increasing in prevalence.^14^ The overuse of PEG in mRNA-LNP vaccine formulations urgently necessitates the development of new PEG alternatives which convey the benefits of PEG while avoiding neutralizing anti-PEG antibodies.^13–15^

Existing work into the discovery of PEG alternatives for mRNA-LNPs has often followed a “one-by-one” approach. PEG alternatives including poly­(glycerol), poly­(vinylpyrrolidone), and zwitterionic polymers have been explored for lipid nanoparticle applications; however, these approaches have been investigated largely as individual polymer candidates, rather than through the systematic exploration of diverse polymer chemistries to identify broadly applicable PEG alternatives.^16^ Crucially, even promising individual PEG-alternative polymers such as poly­(sarcosine) and poly­(oxazoline) still stimulate an adverse antipolymer immune response, meaning individually they will not solve the general problem of antipolymer immunogenicity.^13,17–19^ Akin to antibiotics losing efficacy when overused, a wide selection of PEG alternatives is likely needed to secure the future efficacy of mRNA-LNP therapies. Therefore, a broader approach to the discovery of PEG alternatives must be taken.

To address this, we have developed a family of effective replacements for PEG-lipids in LNP formulations: N-substituted poly­(acrylamido) lipids (PAM-lipids). The synthetic control offered by synthesizing PAMs via reversible addition–fragmentation chain transfer (RAFT) polymerization allowed us to generate a library of 30 chemically varied PAM-lipids, all conjugated to the dimyristoyl glycerol (DMG) lipid, as DMG-PEG conjugates are used in FDA-approved LNP formulations. By using this library to replace PEG-lipids in Moderna’s clinical LNP formulation (SpikeVax), we explored the key chemical design features of PAMs that result in mRNA-LNPs with favorable small particle sizes (∼100 nm) and high transfection efficiencies *in vitro* compared to PEGylated LNPs. As initiators of adaptive immunity after vaccination, dendritic cells (DCs) are key targets for >80% of RNA-LNP clinical trials.^2^ We therefore demonstrate that PAMs are superior alternatives to PEG in terms of mRNA-LNP transfection efficiency of immortalized and primary DCs *in vitro* (up to 120-fold), and in monocytes after 3 weeks of storage. We have characterized the nanoparticle structure of PAM-LNPs, and investigated their use across different common routes of administration (IM, ID, IV), with multiple PAM-LNPs showing effective transfection *in vivo*. In particular, DMA-based PAM-LNPs significantly enhanced mRNA expression within lymph nodes (2.5-fold), and increased *in vivo* transfection of myeloid cells in draining lymph nodes by up to ∼4-fold (macrophages and DCs), compared to PEG-LNPs. Crucially, we demonstrate that PAM-lipids avoid anti-PEG antibodies to recover the lost transfection efficiency caused by repeated dosing of PEG-LNPs. Finally, we discover that PAM monomer chemistry, rather than the end group, is the key structural feature for polymers to effectively evade antipolymer antibody binding. Ultimately, these data establish PAM-lipids as a family of effective replacements for PEG-lipids in mRNA-LNP technologies.

## Results and Discussion

### PAM-Lipids with −H or −OH End Group Enhance Efficacy of mRNA-LNPs Compared to PEG-Lipids

To produce effective PEG alternatives, we synthesized a library of poly­(acrylamido) lipids (PAM-lipids) via reversible addition–fragmentation chain transfer (RAFT) polymerization (Figure 1A, Schematic S4). This allowed us to produce a chemically diverse library of PAM-lipids, with highly controlled polymer chemistry, molecular weights, and end groups, in agreement with results reported for other polymers synthesized via RAFT polymerization.^20^ These parameters were hypothesized to be critical design parameters for LNP function. RAFT polymerization was mediated by a synthesized dimyristoylglycerol (DMG)-functionalized chain transfer agent (CTA) (Schematic S1–S4, Figures S13–S17) to impart a DMG lipid tail end group to the polymer, which enables integration into the LNP bilayer. Five substituted acrylamide monomers with different chemical properties (e.g., functional groups, steric bulk) were chosen due to their hydrophilic nature, which is a key property of PEG in stabilizing LNPs. These monomers were used to prepare polymers of two chain lengths (degree of polymerization, DP = 20 or 60). These specifications were chosen to roughly match the molar mass (DP20, ∼2000 Da), fully extended length (DP60, ∼120 atoms long), and lipid chain end (DMG) of the PEGylated lipid DMG-PEG_2k_ used in Moderna’s SpikeVax formulation.^1,21–23^ The native −S_3_C_5_H_9_ trithiocarbonate end group of all PAM-lipids was then modified to either −OH or −H via oxidation or hydrogen radical donation, respectively, resulting in a library of 30 unique PAM-lipids, each with one of five monomers, two chain lengths, and three end groups. ^1^H NMR spectroscopy confirmed high monomer conversion (>95%), that the expected structure was synthesized, and that any residual monomer was removed during purification (Table S5, Figures S18–S37). Gel permeation chromatography (GPC) confirmed efficient RAFT-mediated control of molecular weight and dispersity from using the DMG-CTA, with PAM-lipids displaying low dispersities (mean dispersity = 1.16 ± 0.10). GPC-coupled UV/vis detection at 309 nm verified the presence, and subsequent removal, of the trithiocarbonate end group, corresponding to polymers with the −S_3_C_5_H_9_ or −OH/–H end groups, respectively (Table S1, Figure S9).

**Figure 1:**
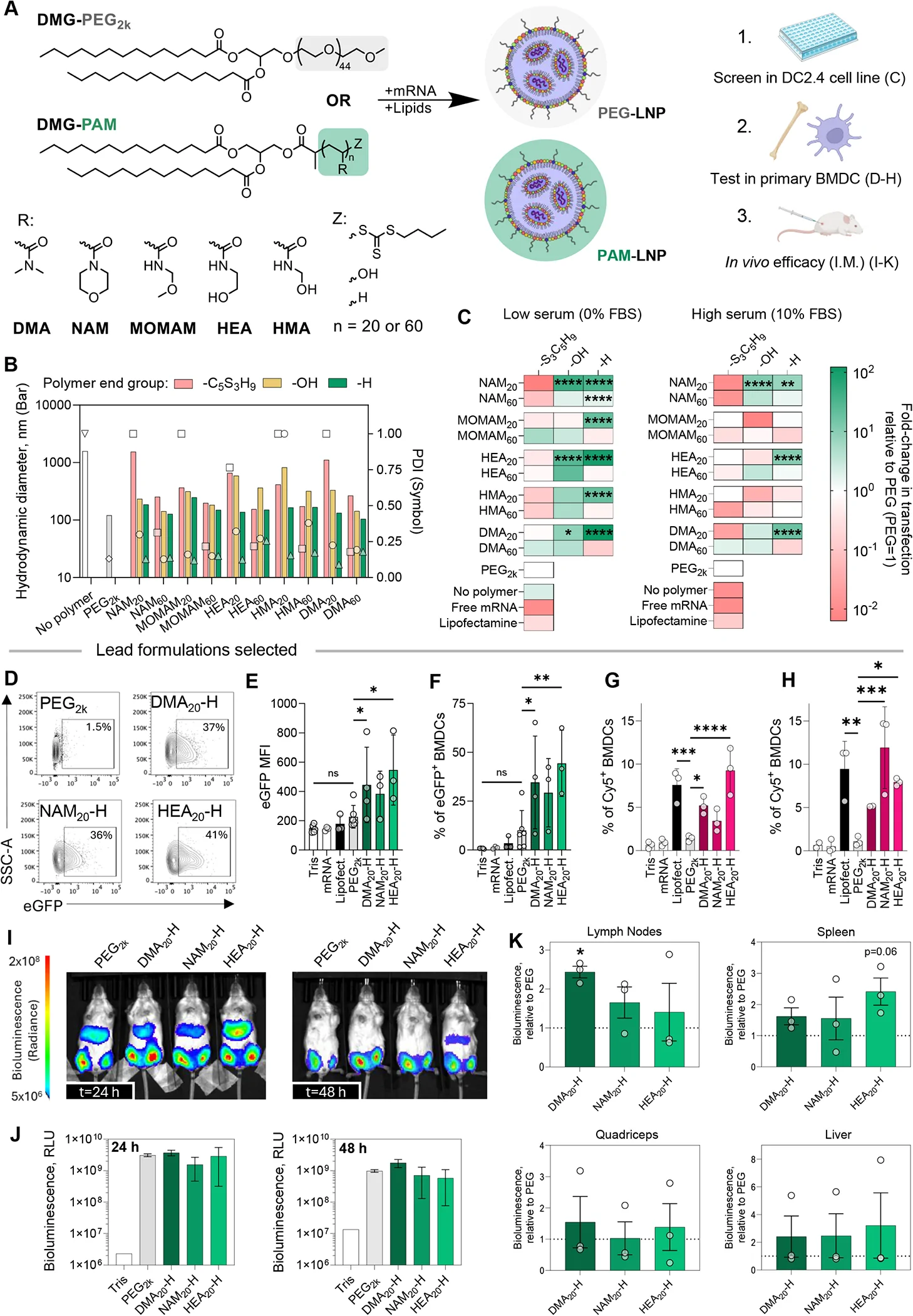
PAM-lipid chemistry modulates LNP size and mRNA delivery performance *in vitro* and *in vivo*. (A) Formulation of PAM-LNPs from synthesized DMG-based PAM-lipids with varied chemistries (5 monomers, 3 end groups and 2 molar masses) and control PEG-LNPs, and the following testing cascades. R = monomer/polymer side group; Z = end group; *n* = target number of repeating units. (B) Z-average hydrodynamic particle diameter (intensity) and polydispersity index of LNPs in Tris (50 mM, pH 7.4) at 25 °C from dynamic light scattering. (C) Firefly luciferase (FLuc) activity (relative to control PEG-LNPs) in DC2.4 cells 24 h after treatment with PAM-LNPs loaded with mRNA encoding firefly luciferase in Opti-MEM, with or without supplemental fetal bovine serum (10% w/v). Data are presented as the mean of *n* = 3 technical replicates, *n* = 3 biological replicates; statistical significance was determined by one-way ANOVA against the control PEG-LNPs. *P < 0.05, **P < 0.01, ***P < 0.001, ****P < 0.0001. eGFP expression in BMDCs treated with mRNA-LNPs encoding eGFP, assessed via flow cytometry: (D) representative pseudoplots; (E) median fluorescence intensity; and (F) percentage of eGFP^+^ cells. Quantification of Cy5^+^ BMDCs treated with LNPs containing Cy5-tagged eGFP mRNA via flow cytometry in high-serum (G) and low-serum (H) conditions. Data are presented as mean ± SEM of biological replicates (*n* = 6 for Tris and PEG_2k_, *n* = 4 for DMA_20_-H, *n* = 3 for all other conditions); statistical significance was determined by a mixed effects model with Dunnett’s correction compared against the control PEG-LNPs. *P < 0.05, **P < 0.01. *In vivo* evaluation of FLuc mRNA (2.5 μg of mRNA per quadricep, 2 injections per mouse) expression after delivery by PAM-LNPs following IM administration represented as absolute whole-body bioluminescence at 24 and 48 h postadministration (I, J) and as individual tissues excised after 48 h (K) relative to PEG-LNPs. Quadriceps and lymph node data points were summed from each quadricep and corresponding draining lymph node for each animal, respectively (*n* = 2 lymph nodes per animal). Data are presented as mean ± SEM of biological replicates (*n* = 4 for PEG_2k_; *n* = 3 for DMA_20_-H, NAM_20_-H, and HEA_20_-H; *n* = 1 for Tris control); statistical significance was determined by Welch one-way ANOVA against the control PEG-LNPs, with Dunnett’s correction. *P < 0.05. Schematic created with BioRender.

PAM-lipids were then used to prepare LNPs (PAM-LNPs) by directly replacing the DMG-PEG_2k_ in Moderna’s commercial SpikeVax formulation (Figure 1A). Otherwise, the formulation was kept the same regardless of the polymer used, comprising: ionizable lipid SM-102, DSPC, cholesterol, and either DMG-PEG or a DMG-PAM at a 50:10:38.5:1.5 molar ratio, respectively. An organic solution of lipids was rapidly mixed with mRNA dissolved in an acidic aqueous buffer to produce PEG-LNPs or PAM-LNPs at a nitrogen-to-phosphate ratio (N/P) of 6, following established protocols.^24^ Particle size measurements revealed that over half of the formulations containing PAM-lipids produced LNPs below 200 nm diameter and PDI < 0.25, similar to the PEG-2k LNP (∼120 nm) (Figures 1B, 3D, 4A). The majority of LNPs derived from DMG-PAMs that retained the DMG-CTA’s native hydrophobic trithiocarbonate end group (−S_3_C_5_H_9_) exhibited larger particle diameters (>200 nm) and higher PDI values (>0.25) than when this end group was replaced with −OH or −H (Figure 1B). In contrast, PAM-LNPs with −OH and −H end groups predominantly yielded smaller and more uniform LNPs, indicating the benefit of nonhydrophobic end groups to stabilize LNP formation. mRNA encapsulation efficiency was >90% for all PAM-LNPs and PEG-LNPs (Figure S1).

The efficacy of mRNA encapsulated within PAM-LNPs was first screened in a DC line (DC2.4) as DCs are a crucial target for key therapeutic applications of mRNA-LNPs (Figure 1C).^2^ This is in part due to their role as professional antigen-presenting cells during the generation of vaccine-induced immunity.^25^ PAM- and PEG- LNPs were incubated with cells in high and low serum conditions (Opti-MEM, ±10% FBS w/v, respectively) for 24 h. Figure 1C demonstrates that 9/30 PAM-LNPs significantly outperformed the control PEG-LNP transfection efficiency in either low serum (up to 120-fold) or high serum media (up to 20-fold). Inclusion of serum in *in vitro* studies of mRNA-LNPs is often overlooked, but improves the physiological relevance of the assay given PEG’s role in controlling protein adsorption to-, and lipoprotein integration into-, the LNP surface when exposed to serum after injection.^26,27^ Which serum proteins adsorb to nanoparticle surfaces can change between particles with different polymer surface chemistries,^28–30^ which in turn can impact LNP uptake/endosomal escape,^31^ inflammatory response,^32^ and organ biodistribution.^33^ Moreover, serum can abstract PEG-lipids, modulating LNP uptake, endosomal escape, and mRNA efficacy.^32–38^ Serum proteins such as apolipoprotein E can also restructure LNPs and cause cargo leakage.^39^ Unsurprisingly then, LNPs prepared without a PEG-lipid or PAM-lipid transfected ∼100-fold less effectively than PEG-LNPs in high serum conditions, despite transfecting well in low serum media (Figure 1C). Broadly, the highest transfection efficiencies were observed for LNPs prepared with short ∼DP20 PAM-lipids bearing either hydrophilic −OH or −H end groups, while near-complete loss in activity was regularly seen when PAMs with the more hydrophobic −S_3_C_5_H_9_ end group were used. This was not due to any noticeable toxicity (Figure S2), but instead large particle size may reduce the efficiency of endocytic uptake.^40^ This end group effect on mRNA efficacy was most pronounced for short DP20 polymers (Figure S3), where the end group represents a greater portion of the polymer as a whole. Generally, the −H end group was best for DP20 polymers, while −OH was best for DP60 polymers. These data highlight how surface polymer chemistry can greatly modulate LNP efficacy. In particular, the presence/absence of small hydrophobic end groups are a disproportionately impactful parameter for the design of polymers for mRNA-LNPs, considering their small size relative to that of the polymer chain (∼5% of polymer molecular weight), which itself makes up only ∼6% of the whole LNP formulation mass. As such, the −S_3_C_5_H_9_ end group is only ∼0.3% of the total mRNA-LNP formulation by weight, but can modulate efficacy across 4 orders of magnitude. To the best of our knowledge, this has not yet been reported as a key design feature of PEG-alternative polymers.

To determine whether PAM-LNPs were also superior at transfecting primary DCs, we used three high-performing PAMs (DMA_20_-H, HEA_20_-H and NAM_20_-H) from the screen in DC2.4s to prepare LNPs loaded with eGFP-encoding mRNA. Bone marrow-derived DCs (BMDCs) were generated from murine bone marrow (C57BL/6) cells cultured with Flt3L, incubated with PAM-LNPs or PEG-LNPs, and analyzed via flow cytometry (gating strategy demonstrated in Figure S10). All three PAM-LNPs effectively transfected BMDCs, with DMA_20_-H and HEA_20_-H LNPs both significantly outperforming the benchmark PEG-LNPs, increasing the percentage of transfected cells to 34% and 44%, respectively, compared to 10% with PEG. Transfection using fluorescently tagged mRNA (Cy5-mRNA) revealed increased association of PAM-LNPs (∼4–10x increase) by BMDCs compared to PEG-LNPs (Figure 1D–H, Figure S4A–G) in both high- and low-serum media. This is likely due to increased cellular uptake and/or retention of LNPs (e.g., via increased endosomal escape), which could mechanistically explain how modulating LNP surfaces with PAMs increases mRNA-LNP transfection in BMDCs compared to PEG. Replacing PEG with these lead PAM formulations did not impact cellular viability (Figure S2) or activation (CD86 expression, Figure S4D) of BMDCs.

We then investigated if the superiority of PAM-LNPs at mRNA transfection *in vitro* translated to enhanced mRNA expression *in vivo* and whether PAM-LNPs with different surface chemistries had different tissue tropisms. We injected the same three high-performing PAM-LNP formulations prepared with mRNA encoding firefly luciferase (fLuc) intramuscularly (IM) into BALB/c mice, as IM is a common route of administration for vaccines/immunotherapies.^41,42^ Bioluminescence location and intensity were imaged 24- and 48 h postinjection. This showed that all 3 PAM-LNPs are effective transfection reagents, with most expression visualized at the site of injection (muscle) and in the liver for all formulations (Figure 1I and J). Direct measurement of excised tissues after 48 h revealed that DMA_20_-H LNPs showed a significant 2.5-fold increase in mRNA expression in lymph nodes relative to PEG-LNPs (Figure 1K). We suggest that by screening PAM-LNPs in serum-containing media and relevant cell types (high-serum, DC2.4s) and confirming their efficacy in BMDCs, we have identified PAM-LNP formulations with a high affinity for tissues rich in immune cells. mRNA expression was otherwise comparable between PEG-LNPs and PAM-LNPs in the quadriceps and liver. It should be noted that the exact composition of an LNP protein corona may differ between FBS and mouse (/human) serum; however, *in vivo* experiments (Figure 1I–K) correlated well with our *in vitro* data, indicating that the protein coronas had similar species-independent effects and ultimately that FBS is a suitable way to increase the biological relevance of *in vitro* conditions compared to serum-free media. Together, these data demonstrate that replacement of PEG-lipids with PAM-lipids in mRNA-LNP formulations results in enhanced transfection of DCs *in vitro* and antigen expression in lymphoid organs *in vivo*, particularly after selecting an optimal polymer chain length (DP20) and end group (−H).

In particular, LNPs prepared with DMA_20_-H displayed more potent performance than PEG in primary DCs and immune-rich tissues and are therefore promising for vaccine and immunotherapy applications. Despite only representing 1.5 mol % of the formulation, polymer lipids (and in particular their end groups) are furthest away from the LNP core and thus likely to interact first with biological entities and strongly influence potency. Promisingly, PAMs prepared from 3 of the 5 monomers investigated (DMA, NAM, and HEA) can be used to replace PEG without compromising particle size, *in vitro*, or *in vivo* performance, highlighting PAMs as a family of useful polymers to replace PEG in LNP-mediated mRNA delivery.

Having established the efficacy of PAM-lipids for mRNA-LNPs *in vitro* and *in vivo*, we sought to investigate (i) if PAM-LNPs improved transfection in immune cells *in vivo*; (ii) if the improved efficacy by using PAM-lipids over PEG-lipids could be attributed to changes in LNP structure or stabilization; and (iii) if PAM-lipids recovered lost transfection in the presence of circulating anti-PEG antibodies during repeated dosing. We address each of these in the following three sections.

### PAM-LNPs Enhance mRNA Expression in Macrophages and DCs Compared to PEG_2k_-LNPs

Having demonstrated that PAMs enhanced the mRNA transfection efficacy of mRNA-LNPs in immune cells *in vitro* and in lymphoid organs *in vivo* compared with the clinical gold-standard PEG-LNPs, we sought to investigate transfection efficiency at a single-cell resolution *in vivo*. We performed spectral flow cytometry analysis on liver, spleen, draining lymph node (dLN), and contralateral lymph node (cLN) from C57BL/6 mice at 24, 48, and 72 h following intradermal (ID) injections of LNPs prepared with PAM-lipid DMG-DMA_20_-H (DMA-LNPs) or DMG-PEG_2k_ (PEG-LNPs), containing mRNA encoding mCherry as a marker for positive transfection (Figure 2A).^43,44^

**Figure 2:**
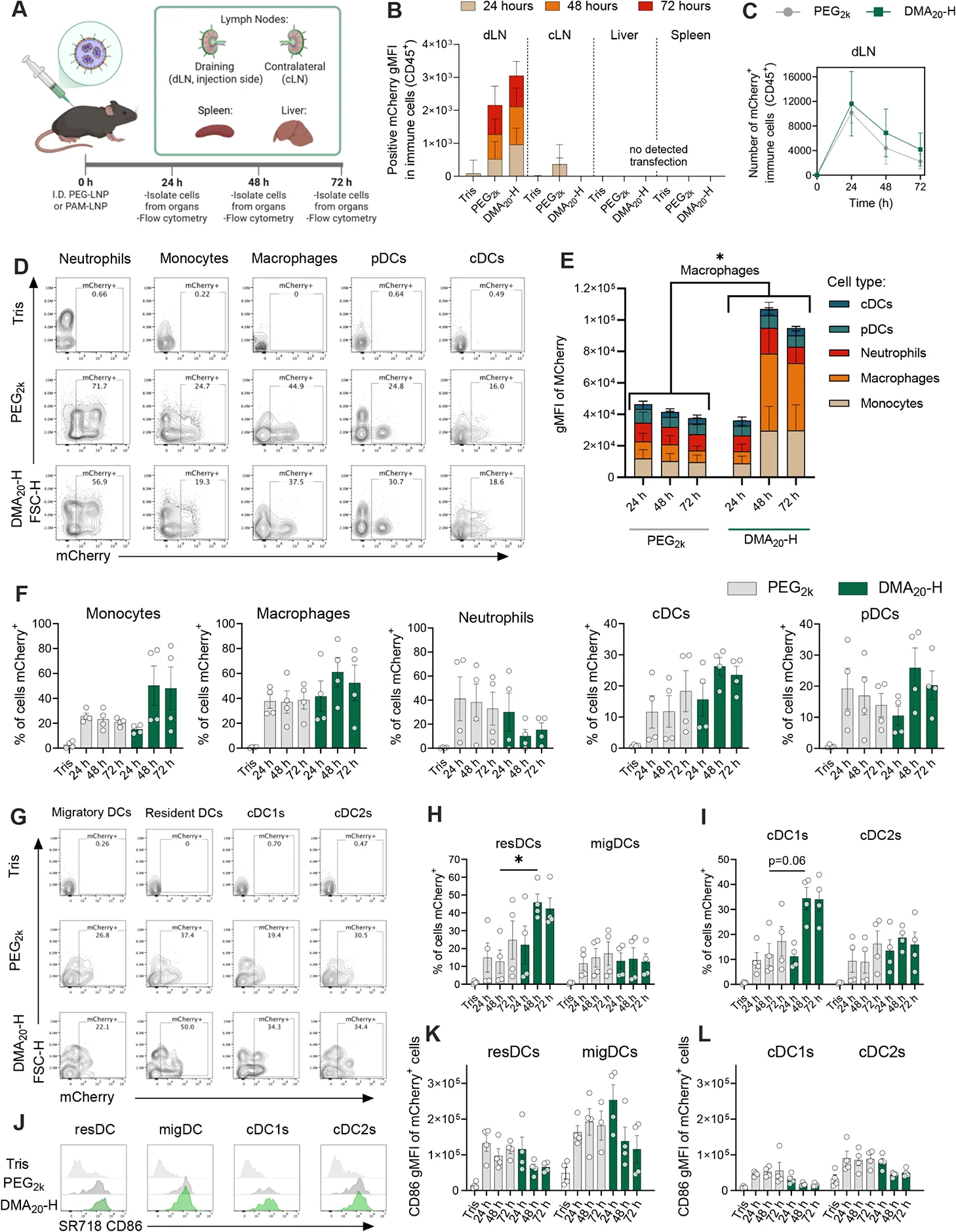
DMA-LNPs improve transfection of myeloid cells relative to PEG-LNPs in draining lymph nodes: (A) Schematic showing experiment where C57BL/6 mice were injected ID with mCherry mRNA-LNPs (3 μg mRNA), formulated with PEG-lipids or the PAM-lipid DMA_20_-H, or Tris buffer control. Liver, spleen, draining lymph node (dLN), and contralateral lymph node (cLN) were harvested for analysis via spectral flow cytometry. (B) Stacked bar chart of mCherry-positive geometric mean fluorescence intensity (gMFI) of CD45^+^mCherry^+^ cells at 24, 48, and 72 h. Data are presented as mean ± SEM, *n* = 4 biological repeats pooled from 2 independent experiments. Statistical significance was determined by two-way ANOVA. (C) Line graph showing the number of live CD45^+^ mCherry^+^ cells at 24, 48, and 72 h. Data are presented as mean ± SEM, *n* = 4 biological repeats pooled over 2 experiments. (D) Representative flow plots showing mCherry expression (gMFI) in gated immune cell populations (CD45^+^) in draining LNs (see Figure S12 for gating strategy). (E) Stacked bar graph showing mCherry gMFI across myeloid cell types at 24, 48, and 72 h. (F) Bar graphs showing the percent (%) of mCherry^+^ cells in dLN across myeloid cell types over 24, 48, and 72 h. Significance determined via Kruskal–Wallis test followed by Dunn’s posthoc test for multiple comparisons. (G) Representative flow plots showing mCherry expression in migratory DCs, resident DCs, cDC1s, and cDC2s (see Supplemental Figure S12 for gating strategy), *n* = 4 biological repeats pooled over 2 experiments. (H) Bar graph showing mCherry+ frequency of migratory and resident DCs (white: Tris, gray: PEG-LNPs, green: DMA-LNPs). Data are presented as mean ± SEM, *n* = 4 biological repeats pooled over 2 experiments. Statistical significance was determined by two-tailed Mann–Whitney test, *P < 0.05. (I) Bar graph showing mCherry+ frequency of cDC 1s and cDC 2s (white: Tris, gray: PEG-LNPs, green: DMA-LNPs). Data are presented as mean ± SEM, *n* = 4 biological repeats pooled over 2 experiments. Statistical significance was determined by two-tailed Mann–Whitney test, *P < 0.05. (J) Representative flow cytometry histogram plots of CD86 expression of total BMDCs in the Tris control, to mCherry+ resident DC, migratory DC, cDC1, and cDC2 populations (Light gray: Tris, gray: PEG-LNPs, green: DMA-LNPs), *n* = 4 biological repeats pooled over 2 experiments. Bar graphs showing CD86 gMFI of resident DCs, migratory DCs (K) and cDC 1s and cDC 2s (L) (white: Tris, gray: PEG-LNPs, green: DMA-LNPs). Data are presented as median with range, *n* = 4 biological repeats pooled over 2 experiments. Significance determined via Brown-Forsythe and Welch one-way ANOVA test followed by Dunn’s posthoc test for multiple comparisons. Schematic created with BioRender.

Analysis of all organs collected revealed that mCherry expression was the greatest in dLNs (Figure 2B). The number of mCherry^+^ immune cells (CD45^+^) in the dLNs showed similar kinetics across both formulations, peaking at 24 h and decreasing at 48 and 72 h (Figure 2C). Transfection was primarily driven by myeloid cells (Figure 2D–L), with little transfection of lymphocytes (Figure S12).

Interrogation of transfected cells within dLNs demonstrated that macrophages, monocytes, neutrophils, conventional DCs (cDCs), and plasmacytoid DCs (pDCs) showed comparable transfection efficiencies between PEG-LNPs and DMA-LNP formulations (Figure 2E and F). However, we observed a significant increase in the transfection of macrophages from mice that had been injected with DMA-LNPs, driven by 4-fold increase in transfection intensity (gMFI) at 48- and 72 h time points (Figure 2E). Increased transfection of macrophages can adjuvant immunotherapy responses^45^ and could also benefit various other therapeutic mRNA targets including those related to inflammatory diseases, wound healing, and nerve regeneration.^46,47^

Finally, a key aim for vaccine design is targeting and activation of DCs to prime the naive T cell response. As such, we studied transfection across various DC subpopulations (Figure 2G–L). cDC 1s are a subpopulation of cDCs that are highly specialized for cross-presentation mechanisms, meaning they are extremely efficient at promoting the CD8+ T cell activation required for effective vaccination and are therefore central to many vaccination strategies.^48–50^ DMA-LNPs effectively transfected this subpopulation, although the ∼3-fold increase in transfection compared to PEG-LNPs after 48 h was not significant (*p* = 0.06) (Figure 2I). Crucially, however, injection of DMA-LNPs significantly enriched the percentage of mCherry^+^ transfection of DCs resident to the LNs (resDCs, MHC^lo^ CD11c^+^) at 48 h over PEG-LNPs (Figure 2H). This increase in LN-resident DCs may be due to DCs directly transfected by LNPs that have drained to the LNs, or they can acquire mRNA-encoded antigen from migrating DC which can amplify the T cell priming response.^50–52^

Transfected DCs expressed high levels of the costimulatory ligand CD86 across all groups of PEG-LNPs and DMA-LNPs compared to Tris control, suggesting that they were effectively poised to prime T cells (Figure 2J–L). This was consistent with our *in vitro* BMDC data in which increased CD86 expression was observed across formulations (Figure S4D), likely due to the fact that the most immunostimulatory components of the formulations (ionizable lipid, single-stranded mRNA) were consistent between formulations.^53^ Taken together, these data demonstrate that PAM-LNP formulations prepared with DMA_20_-H outperform PEG-LNPs at transfecting myeloid immune cells in the dLNs after ID administration, aligning with increased transfection by DMA-LNPs of primary and immortalized DCs (Figure 1C–F) and in the lymph node after IM injection (Figure 1I–K). This is particularly promising, as it suggests that using DMA-LNPs in place of PEG-LNPs can increase the transfection in cells in the LNs independent of mRNA cargo, route of administration, or animal strain. Re-engineering mRNA-LNP surfaces with PAM-lipids therefore presents a promising strategy to increase mRNA transfection without compromising activation and highlights the potential of PAM-lipids to stimulate vaccination/immunotherapeutic immune responses.

### PAM-Lipids Form Similar LNP Structures to PEG-Lipids with Improved Efficacy before and after Storage

Having characterized the biological efficacy of PAM-lipids and identified DMA_20_-H, NAM_20_-H, and HEA_20_-H as effective replacements for PEG_2k_ in mRNA-LNP formulations, we then sought to establish how polymer–lipid chemistry affected LNP size, morphology, internal structure, and stability. Any changes in these physicochemical properties could explain the ability of PAMs to enhance the mRNA-LNP efficacy *in vitro* and *in vivo*. PEG-lipids also have a crucial role in stabilizing the LNP structure during the formulation process by inserting into the surface of the nanoparticles after self-assembly and preventing excessive particle agglomeration. LNPs prepared with PEG_2k_, DMA_20_-H, NAM_20_-H, or HEA_20_-H were analyzed via cryo-electron microscopy (cryo-EM), small-angle neutron scattering (SANS), small-angle X-ray scattering (SAXS), and dynamic light scattering (DLS) as orthogonal techniques to characterize particle size, morphology, internal structure, and heterogeneity (Figure 3A–E).

**Figure 3:**
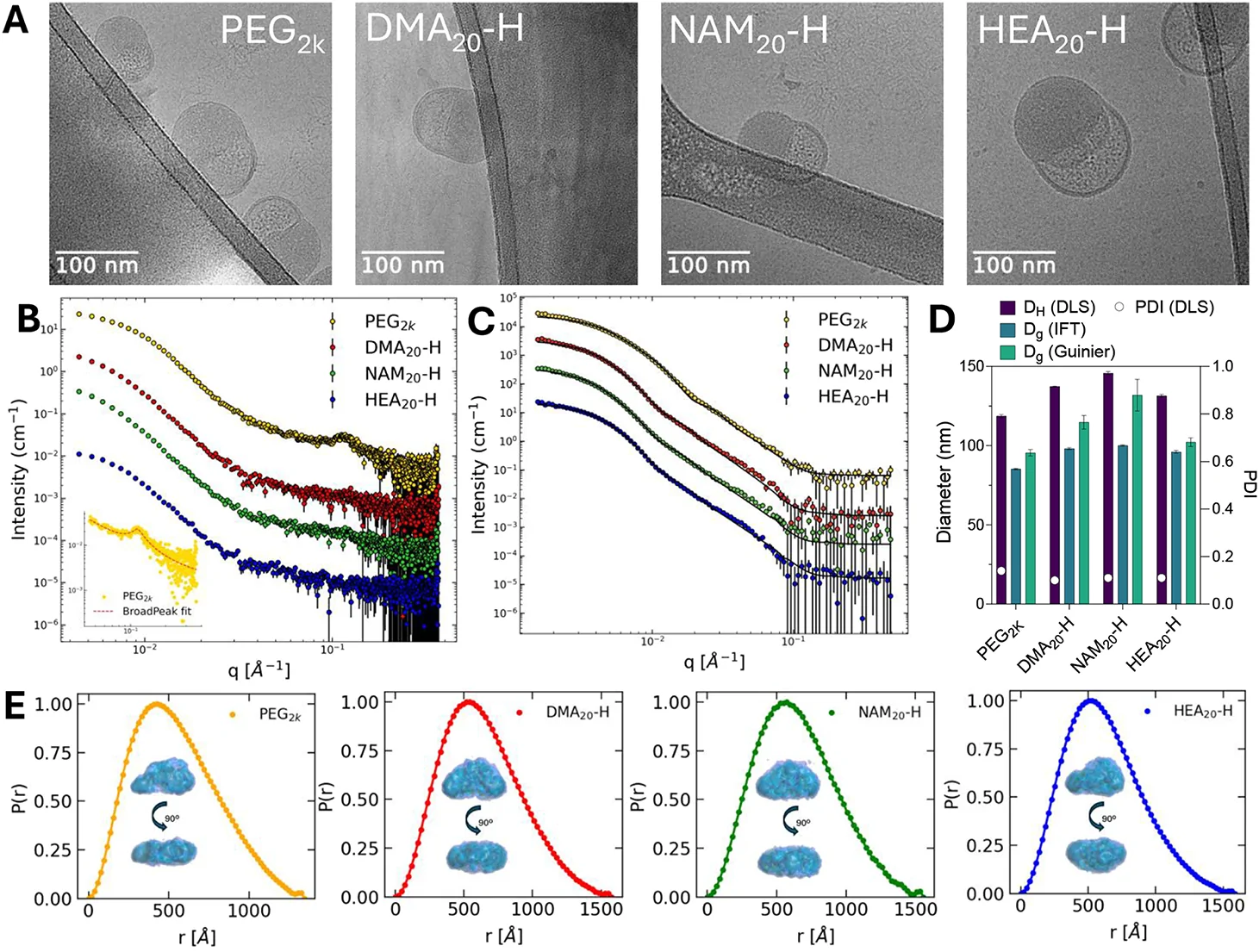
PAM-lipids and PEG-lipids produce LNPs with similar internal structure and morphology. (A) Cryo-electron microscopy images of LNPs prepared with PEG-lipids or PAM-lipids, loaded with eGFP mRNA. (B) SAXS and (C) SANS profiles of LNPs prepared with PEG-lipids or PAM-lipids loaded with poly­(A), with the inset of (B) showing a broad peak fit of the high-q PEG_2k_ experimental data. The solid lines are fits using a core-two-shell cylinder model. (D) Hydrodynamic diameter (Bar, left *Y*-axis) and polydispersity (PDI, symbols, right *Y*-axis) of LNPs prepared with PEG-lipids or PAM-lipids, loaded with eGFP mRNA, analyzed with dynamic light scattering (DLS). Data presented as mean ± SD, *n* = 3, and compared to the diameter of gyration from the Guinier analysis (Table S2) and the *P*(*r*) evaluation. (E) *P*(*r*) analyses and DENSS *ab initio* reconstructions (insets) of SANS data.

DLS revealed that the nanoparticle size (hydrodynamic diameter, D_H_) and polydispersity were 100–150 nm and ∼0.1, respectively, regardless of the polymer–lipid used (Figures 1B, 3D, 4A), with particle sizes (Diameter of Gyration, *D*_g_) determined via SANS analysis similar in magnitude (Figure 3D). Cryo-EM images revealed slightly elongated structures with phase separation between the internal aqueous and lipid-rich compartments (“blebbing”) observed in all formulations (Figure 3A), consistent with the bleb structures observed for PEGylated mRNA-LNPs in other studies. Blebs have been suggested as crucial for effective mRNA transfection.^54^ SAXS analysis of the internal structure revealed a small Bragg peak at *q* = 0.11 Å^–1^ in LNPs prepared with PEG_2k_, corresponding to an internal mesophase of ∼5.5 nm, consistent with other PEGylated mRNA-LNPs (Figure 3B).^55,56^ Interestingly, this peak was not well resolved in the PAM-LNPs, suggesting a less ordered internal structure. Decreases in this peak, but not complete disappearance, have been seen in other reports of LNPs prepared with other PEG alternatives that also improved mRNA efficacy.^56^ This elongated, anisotropic structure observed in cryo-EM was confirmed with SANS *P*(*r*) pair distance distribution function analysis and *ab initio* three-dimensional structures (DENSS; insets in Figure 3E, Table S1)^55,57,58^ and is consistent with the core–shell bleb morphology seen in cryo-EM (Figure 3A). Model-based analysis (cylindrical core-two-shell model, Figure 3C; and core–shell ellipsoid model, Figure S5) implies that the representative LNP shape is elongated with long and short dimensions, respectively, varying from 112 and 26 nm for PEG to 138 and 50 nm for NAM (Table S4). While some report spherical mRNA-LNPs, they are typically prepared with different ionizable lipids to SM-102, such as MC3 (D-Lin-Mc3-DMA).^39,59^ Ultimately, PAM-lipids form LNPs of similar size and morphology to PEG-lipids, potentially with small differences in internal organization.

**Figure 4:**
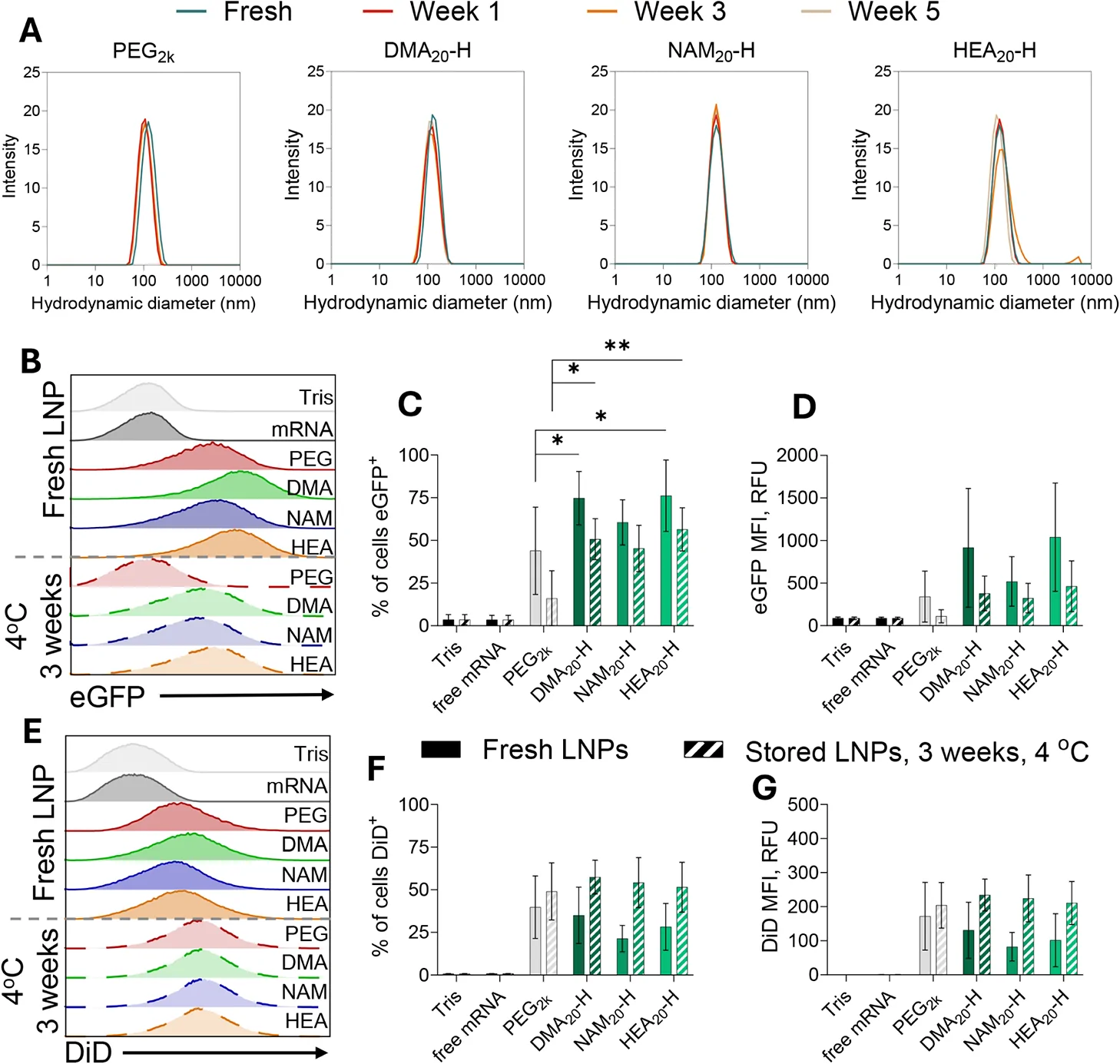
PAM-LNPs retain colloidal stability and biological performance equivalent to PEG-LNPs in fridge storage. PAM- and PEG-LNPs were formulated with eGFP-encoding mRNA then stored at 4 °C for up to 5 weeks. (A) Intensity-weighted particle size distributions as measured by dynamic light scattering. Fresh and stored LNPs containing eGFP mRNA and fluorescently labeled with DiD fluorescent dye were incubated with THP-1 cells for 24 h, compared with free mRNA and an untreated Tris buffer vehicle control analyzed by flow cytometry (gating strategy, Figure S11). Data are represented as flow cytometry histograms (B, E), % of positive fluorescent cells (C, F), and median fluorescence intensity (D, G) for eGFP and DiD, respectively. Data are presented as mean ± SD of *n* = 3 biological repeats. Statistical significance was determined by two-way ANOVA with Dunnett’s test against the control PEG-LNPs. *P < 0.05, **P < 0.01.

Alongside stabilizing LNP structure during the formulation process, one of the key properties that PEGylation bestows on nanoparticles is increased storage stability.^60^ The polymer shell acts as a steric barrier to minimize aggregation which, over time, can change nanoparticle structure and negatively impact biological efficacy, since the efficacy of clathrin-mediated endocytosis begins diminishing for nanoparticles above ∼100 nm.^40^ Effective PEG alternatives must therefore fulfill this stabilizing role. The medium- to long-term instability at fridge temperature of both Moderna and Pfizer/BioNTech mRNA COVID-19 vaccines also posed a significant issue during the early phase of the COVID-19 pandemic vaccine response, requiring these products to be frozen below −20 °C or −80 °C for extended periods.^61^ Hence, innovations to reduce the cold-chain requirement for mRNA LNPs are critical to minimizing the cost and expanding the scope of mRNA medicines. We therefore compared the stability of the three high-performing PAM-lipids, DMA_20_-H, NAM_20_-H, and HEA_20_-H against the benchmark PEG_2k_ in this context. Figure 4A shows that PAM-LNPs and PEG-LNPs had similar initial particle sizes (∼100 nm) and displayed minimal change in particle size (hydrodynamic diameter) over a 5-week period stored at 4 °C.

To investigate the impact of storage on transfection efficiency, 3-week-old and fresh PAM-LNPs (DMA_20_-H, NAM_20_-H, HEA_20_-H) and PEG-LNPs loaded with eGFP mRNA and DiD fluorescent dye were incubated with human monocyte-like (THP-1) cells. As THP-1 cells grow in suspension, they are theoretically less susceptible to aggregates that may form during the storage. This is because aggregates may sink to the bottom (or float) when left for long periods in suspension, such as the 24 h incubation when applied to cells. This can alter the local concentration of nanoparticles above a 2D monolayer.^62,63^ While all formulations displayed a loss in efficacy over 3 weeks of storage, fresh and stored PAM-LNPs containing DMA_20_-H and HEA_20_-H both outperformed PEG-LNPs, showing higher percentages of eGFP-transfected cells, when fresh (75% and 50%, respectively) and stored for 3 weeks (50% and 15%, respectively) (Figure 4B–G). We suggest that the efficacy loss seen during storage is likely due to inherent degradation of the mRNA cargo via hydrolysis or reaction with impurities in the formulation.^61,64,65^ DLS data show that it was not due to changes in LNP size (Figure 4A), and DiD fluorescence data suggested that changes in transfection due to storage cannot be attributed to changes in cellular association/uptake of PAM-LNPs compared to PEG-LNPs (Figure 4E,F,G). Moreover, as PAM-lipids are likely on the surface of the LNPs (as PEG-lipids are), it is unlikely that they would greatly impact degradation of encapsulated mRNA, which could be why efficacy loss occurs in all formulations regardless of polymer surface. Overall, our findings show that PAM-lipids exhibit equivalent colloidal stability and enhanced efficacy after storage compared to PEG-LNPs, highlighting their suitability as potential PEG alternatives.

### PAM-Lipids Do Not Generate—or Bind to—Anti-PEG Antibodies and Rescue Delivered mRNA Expression after Repeated Dosing of PEG-LNPs

The major driver for the development of PEG-alternative LNPs is avoiding the generation of—and binding to—anti-PEG antibodies, which reduce the transfection efficiency of encapsulated mRNA. Anti-PEG antibodies can compromise LNP integrity, leading to cargo leakage,^11,12^ and cause ABC of LNPs from the body.^7,8,10,66^ This is particularly pertinent after the global rollout of COVID-19 vaccination, where anti-PEG antibody IgM increased 69-fold after patient vaccination with Moderna’s SpikeVax mRNA-LNP formulation, as the composition of which is replicated in this study.^5^ It has been previously hypothesized, but not demonstrated, that it may be possible to recover this lost efficacy by dosing LNPs with a PEG-alternative surface, evading opsonization from anti-PEG antibodies which are unable to bind to PEG alternatives with different chemical structures.^13^

Therefore, to determine whether PAM-LNPs are recognized by anti-PEG antibodies, we incubated our leading PEG-alternative PAM-lipid (DMA_20_-H) with anti-PEG IgM antibodies across concentrations relevant to clinically determined anti-PEG antibody titers and tested binding via an enzyme-linked immunosorbent assay (ELISA) (Figures 5A, S7B).^4,5,67,68^ Other PAM-lipids with varied monomer and end group chemistry also did not bind to anti-PEG antibodies (Figure S7A and B). We hypothesized that PAM-LNPs would therefore evade circulating anti-PEG IgM in mice previously injected with PEG-LNPs and result in enhanced transfection efficiencies compared to the PEG-LNP. To test this, we conducted a repeat dosing experiment comparing three different dosing regimens: 1) a single dose of either PEG-LNPs (1PEG) or DMA_20_-H PAM-LNPs (1DMA); 2) four doses of either PEG-LNPs (4PEG) or DMA_20_-H PAM-LNPs (4DMA) administered weekly; and 3) three doses of the PEG-LNP, followed by a fourth dose of the DMA_20_-H PAM-LNP administered weekly (3PEG-1DMA) (Figure 5B). This allowed us to investigate how PAM-LNPs perform in animals with/without circulating antipolymer antibodies.^7,69^ All LNPs were prepared with fLuc mRNA and the fluorescent lipid dye DiR to allow independent tracking of mRNA transfection (bioluminescence) (Figure 5D and E) from LNP organ biodistribution (fluorescence) (Figure 5F and G). Organs and plasma were collected 24 h after the final dose in each regimen and imaged *ex vivo* or analyzed for anti-PEG_2k_ and anti-DMA_20_-H antibody titers, respectively.

**Figure 5:**
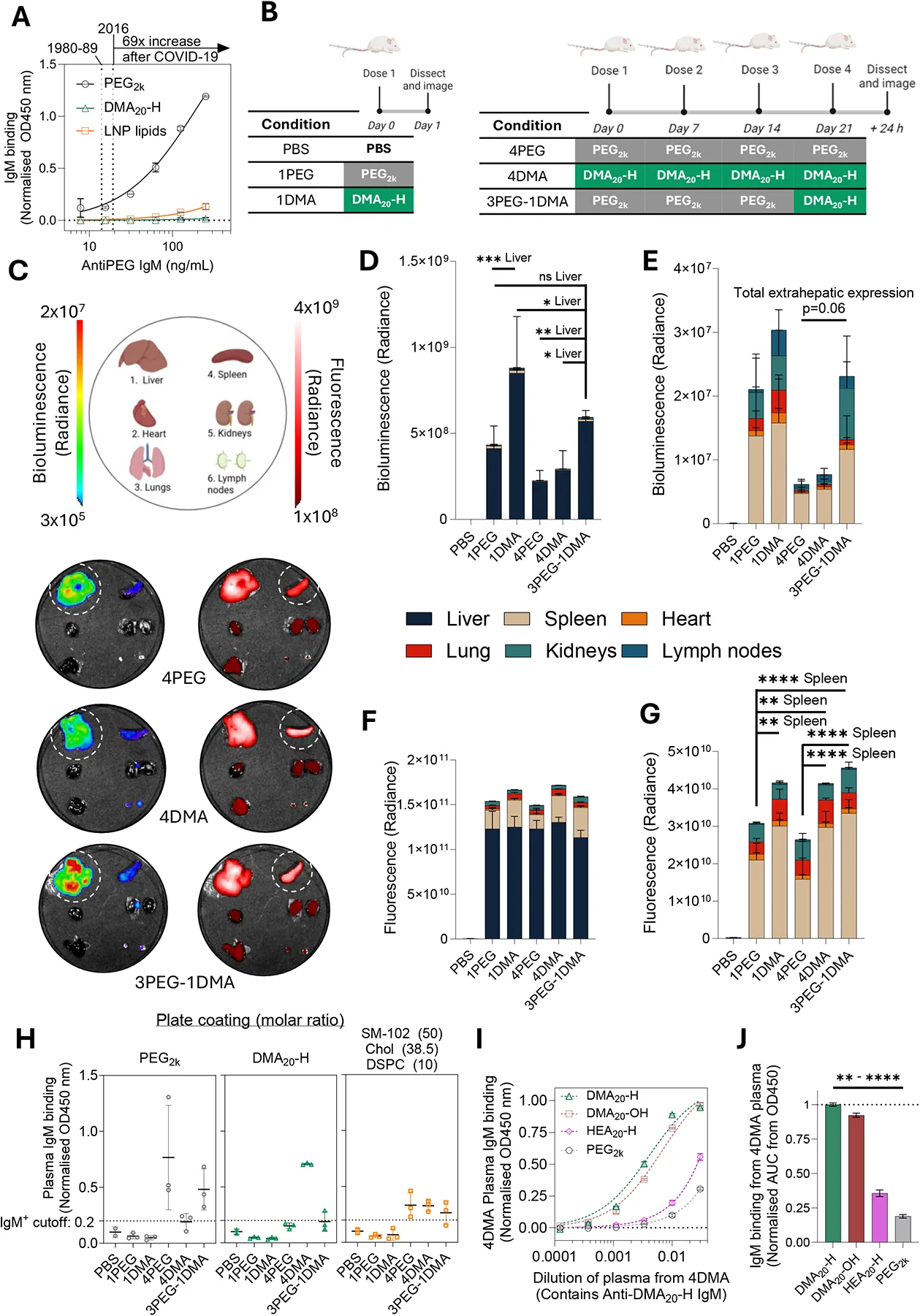
PAM-LNPs recover hepatic and extrahepatic efficacy loss compared to PEG-LNPs when used in heterologous dosing regimen (A) Binding of commercially available murine anti-PEG IgM to PEG_2k_, DMA_20_-H, or nonpolymer LNP components SM-102, cholesterol, and DSPC mixed in 50:38.5:10 molar ratios. Dotted lines represent clinically determined anti-PEG IgM levels from specified years before COVID-19 and the fold increase after COVID-19 vaccination.^4,5^ (B) Schematics showing single dosing and repeat dosing of fLuc mRNA LNPs (5 μg of mRNA per dose) prepared with PAM-lipid DMA_20_-H (green-shaded cells) or PEG-lipid PEG_2k_ (gray-shaded cells). Organs and blood collected 4 h after the final dose (either 1 dose or 4 doses). Organs imaged using *in vivo* imaging system (IVIS) to image bioluminescence (C) from mRNA and fluorescence from FLuc mRNA expression and fluorescently tagged LNP (DiR), respectively. Dotted circles highlight organs with statistically significant differences. Quantified bioluminescence (D, E) and fluorescence (F, G) determined by regions of interest (equal area between images) are presented as the mean of biological repeats ± SEM (*n* = 3 for all LNP conditions; *n* = 2 for the PBS vehicle control). Statistical significance was determined by two-way ANOVA with Tukey’s correction, *P < 0.05. (H) Plasma IgM detected via enzyme-linked immunosorbent assay (ELISA) of plates coated with the specified polymer–lipids/lipids (LNP components). Data were normalized to OD450 nm of the PBS control, set to OD450 = 0.1. IgM^+^ Cutoff set as 2× OD450 nm of the PBS control. Data presented as the mean of biological repeats ± SD (*n* = 3). (I) Binding of IgM in dilutions of pooled anti-DMA20-H Ab+ plasma to different polymer–lipids. (J) Total IgM binding in anti-DMA_20_-H Ab^+^ plasma determined by area under the curve (AUC) integration analysis, normalized to DMA_20_-H. Data for (A, I) and (J) presented as the mean of technical replicates ± SEM (*n* = 3), **P < 0.01, ****P < 0.0001. Schematic created with BioRender.

As expected after IV administration, the majority of the fLuc expression was seen in the liver,^21^ where after a single dose DMA_20_-H LNPs delivered a 2-fold greater total luciferase expression than the benchmark PEG-LNPs (Figure 5C–E, Figure S6A–C). This contrasts with IM and ID administration routes, where DMA-LNPs performed comparably to PEG in the liver, suggesting tissue tropism is dependent upon both polymer–lipid chemistry and route of administration. Interestingly, DMA_20_-H LNPs also showed a significantly higher propensity to transfect LNs (Figure S6D) when normalized for LNP biodistribution, consistent with improvements in lymphoid transfection when administered IM and ID. As previously discussed, repeated injections of PEG-LNPs resulted in an ∼2-fold drop in luciferase expression (Figure 5D) alongside the generation of anti-PEG IgM antibodies (Figure 5H).^7^ DMA_20_-H did not elicit the generation of anti-PEG IgM, suggesting that clinical use of DMA_20_-H would not compromise the efficacy of other future PEGylated doses/drugs (Figure 5H). Interestingly, however, a reduction in mRNA expression was also observed after repeatedly dosing DMA_20_-H LNPs, which also generated anti-DMA IgM. The production of antibodies against polymers other than PEG was not surprising, as prior work has reported antibody formation against other established PEG alternatives poly­(oxazoline) and poly­(sarcosine).^13,17^ Crucially, however, we demonstrate that we can fully recover all lost hepatic and extrahepatic transfection efficacy back to the level of a single-dose PEG LNP (1PEG) by simply swapping only the polymer–lipid used in the final dose formulation, from PEG_2k_ to DMA_20_-H (3PEG-1DMA condition). This is likely due to DMA_20_-H LNPs’ resistance to recognition and subsequent opsonization from circulating anti-PEG IgM, as there was minimal cross-reactive binding of anti-PEG IgM with DMA_20_-H (Figure 5A and Figure S7B). PAM-LNPs are therefore well suited to replace PEG-LNPs in both single-dose and repeated dosing applications, particularly in patient populations with pre-existing anti-PEG antibodies (up to 90% of people).^5^

We noted that while the level of luciferase expression from the final dose of DMA_20_-H LNPs in 3PEG-1DMA fully recovered to the level of 1PEG, which was our key end point, it did not entirely recover to the levels of a single dose of DMA_20_-H LNPs in 1DMA. We hypothesized that this effect may arise from opsonization by nonpolymer related antibodies which are generated from LNP doses. To investigate antibodies against nonpolymer components within the LNPs (e.g., ionizable lipid, cholesterol, and helper lipids), ELISAs were conducted using plates coated with all of the lipids found in the PEG LNPs, DMA_20_-H LNPs (Figure 5I and J, Figure S7C and D) and a three-lipid mixture corresponding to the LNP formulation but without a polymer–lipid (i.e., only SM-102, DSPC, and cholesterol; Figure 5H). Analysis of plasma from repeated dosing experiments revealed low levels of “anti-lipid” antibodies in all repeat dose regimens (4PEG, 4DMA, and 3PEG-1DMA). These antilipid antibodies could be responsible for incomplete recovery of transfection by 3PEG-1DMA to the initial level of 1DMA. The presence of these anti-LNP antibodies is rarely assessed but we propose they should be monitored more frequently by those assessing the immunogenicity of mRNA-LNPs, particularly as anti-LNP antibodies have also been detected in the population.^70^ Nonetheless, our data suggest that antipolymer antibodies are the main driving force for LNP sensitization, causing efficacy loss after repeat dosing, and highlighting the importance of this feature in future mRNA-LNP design.

Assessing the biodistribution of LNPs via DiR fluorescence highlighted that, aside from the expected liver-dominated biodistribution,^71^ PAM-LNPs had significantly increased biodistribution to the spleen over PEG-LNPs (Figure 5C,F,G), although the 1.1- and 2.5-fold increases in splenic transfection were statistically insignificant. Looking broadly across all dosing regimens and organs showed that there was no evidence of increased LNP clearance due to repeated dosing and indicates that the transfection efficacy loss observed from repeat dosing is not dependent on organ accumulation. This is in contradiction to the conventional wisdom that the reduction in efficacy from antibody opsonization is caused only by increased clearance of nanoparticles.^7^ For example, Pilkington and coworkers have shown that the ABC effect of LNPs in blood is minimized when switching LNP surface chemistries for a two-dose regimen; however, they do not report the impact on mRNA efficacy.^66^ Our data suggest a separate mechanism from ABC as the reason for reduced efficacy during repeat dosing. Instead, opsonization via antipolymer antibodies (and potentially some anti-LNP antibodies) may modulate LNP transfection capability, possibly by impacting the cellular response to LNPs (e.g., reducing uptake or endosomal escape) or by destabilizing LNPs and causing premature mRNA cargo release.^8,11,12,72^ This is corroborated by Figure S6D, which showed that, when mRNA transfection (fLuc bioluminescence) was normalized to LNP biodistribution (DiR fluorescence), the same amount of LNP formulation transfected the same organs to different degrees depending only on the presence/absence of antipolymer antibodies. The underlying mechanism behind avoiding anti-PEG antibody binding with polymers such as DMA_20_-H resulting in improved efficacy is the subject of ongoing work. Overall, to the best of our knowledge, this is the first demonstration of mRNA efficacy recovery in mRNA-LNPs using a heterologous dosing regimen between LNPs with different polymer surfaces, highlighting the potential for PAM-lipids as an alternative to PEG in cases of pre-existing anti-PEG antibodies.

As the reduction in mRNA-LNP efficacy caused by repeated dosing was predominantly mediated by the polymer surface and associated antibodies, we then sought to investigate which features of polymer–lipid chemistry are implicated in antipolymer antibody binding/evasion. Murine plasma positive for anti-DMA_20_-H IgM from the 4DMA condition was tested for binding to polymers with different chemistries: 1) the original antigen (DMA_20_–H); 2) the same polymer but with a different end group (DMA_20_–OH); 3) a PAM-lipid made of a different monomer (HEA_20_–H); and 4) the non-PAM polymer PEG_2k_ (structures in Figure 1A). Some cross-reactivity was seen for all polymers, likely due to polyclonal cross-reactivity with conserved elements present in the chemistry of all of the polymers, such as glycerol linker or DMG lipid tail. While changing end group has been suggested as a potential route to evade existing anti-PEG antibodies,^73,74^ we only observed a modest ∼10% reduction in anti-DMA_20_-H IgM binding with DMA_20_–OH compared to DMA_20_-H. Promisingly, however, we observed that changing the monomer chemistry of the PAM from DMA to HEA significantly alleviated cross-reactivity almost as effectively as switching to an entirely different polymer (PEG_2k_) (Figure 5I and J). This is particularly encouraging as LNPs prepared with HEA_20_-H were effective transfection reagents in primary cells and *in* vivo (Figure 1D–J) and also do not bind to anti-PEG antibodies (Figure S7A and B). As side-group chemistry changes between polymers made of different PAM monomers, but polymer backbone chemistry is conserved, the fact that HEA_20_-H resists anti-DMA_20_-H antibodies suggests that side-group chemistry is the dominant binding epitope and thus is the most judicious aspect of polymer chemistry to redesign for antipolymer antibody evasion. This is a key benefit of the development of the family of PAM-lipids, as PAM-lipids prepared with every tested monomer chemistry showed effective mRNA-LNP transfection *in vitro* and/or *in vivo*, including HEA_20_-H (Figure 1). We therefore propose that, rather than the one-by-one development of new PEG alternatives, PAM-lipids represent a family of viable PEG-alternative polymers for the future development of mRNA-LNPs.

We also note that alongside PAMs, aforementioned existing PEG alternatives poly­(oxazoline), poly­(sarcosine), and poly­(vinylpyrrolidone) all possess substituted amide groups in the repeat-units of their polymer chemistry, albeit arranged differently from a structural perspective (Figure S8).^16–18,75^ This suggests that amide-containing polymers may be particularly suited for producing effective PEG-alternative polymers. Within this context, PAM-lipids provide a distinct benefit over these three alternatives (and also PEG) as PAMs have far greater synthetic accessibility of different monomer chemistries as a result of their synthesis via RAFT polymerization.^20,76^ PAM-lipids, therefore, are uniquely situated to form the basis of a chemically varied family of viable PEG alternatives.

### Limitations and Future Directions

While PAM-lipids effectively replace PEG_2k_ and provide improved mRNA transfection across multiple organs/cell types and during repeated dosing, they do not fully abrogate polymer-directed immune responses. As observed for other polymer–lipids, PAM-lipids remain susceptible to reduced efficacy upon repeated dosing and can still induce antipolymer antibodies, indicating that their use may require monitoring for potential adverse immune-mediated events, including hypersensitivity, in clinical settings. Importantly, although the higher initial transfection efficiency of PAM-lipids may partially mitigate the impact of antibody formation during repeat administration, any single specific PAM-lipid may not represent a universal solution for all mRNA-LNP applications, highlighting the need for a library of viable PEG alternatives for formulators to choose from. Consistent with the low cross-reactivity observed between antibodies directed against PEG_2k_ and PAM-lipids, strategies incorporating multiple polymer–lipids while minimizing repeated patient exposure to any individual chemistry may be required to maximize therapeutic durability of mRNA-LNPs. In this context, further expansion of the PAM-lipid family through exploration of alternative monomer chemistries represents a promising direction for future investigation. Moreover, due to the ubiquity of PEG in other nanoparticle, biologic, and cosmetic formulations, deployment of PAMs could benefit a wide variety of applications outside of gene therapies. Logical starting points would be other formulations susceptible to anti-PEG antibody-mediated ABC or adverse reactions, such as PEGylated liposomes^77^ or peptides (e.g., Pegaspargase).^78^

## Conclusions

We present PAMs as a chemically varied family of polymers suitable for replacing immunogenic PEG in mRNA-LNP formulations. Crucially, we highlight that the polymer chemistry presented on the surface of mRNA-LNPs is a critical design parameter, having profound effects on their absolute efficacy, cell specificity, and antibody evasion despite representing less than 2 mol % of the overall formulation. We observed that small nonhydrophobic end groups are required to promote stable mRNA-LNP formulations with suitable transfection capability, while the polymer side-group is a key parameter for attenuating antipolymer antibody binding. PAM-lipids were found to produce stable LNPs with structures similar to those of benchmark PEG-lipids. The improved mRNA transfection efficacy bestowed by replacing PEG with PAM-lipids is maintained before and after storage. High-performing PAM-lipids significantly enhance transfection efficiency in various organs (lymph nodes, liver) *in vivo* and in various immune cell subpopulations *in/ex vivo*, across varied routes of administration. Finally, we demonstrated that PAM-LNPs can efficiently evade opsonization from circulating anti-PEG antibodies and hence recover the efficacy lost from repeatedly dosing conventional PEGylated mRNA-LNPs. Together, these data establish the potential role of the PAM-lipid family in next-generation mRNA therapeutics and prophylactics.

## Methods

### Materials

All reagents were used as received unless otherwise stated. Materials are listed below.

#### Sigma-Aldrich (Merck)

All solvents, cholesterol, *N*,*N*′-dimethylacrylamide (DMA) monomer, 2-hydroxyethyl acrylamide (HEA), *N*-acryloylmorpholine (NAM), sodium acetate, Tris buffer (1 M), PBS tablets, poly­(A) (polyadenylic acid), RiboGreen RNA assay kit and dialysis tubing (1 kDa MWCO), RPMI-1640 medium (R0883), Amicon Ultra centrifugal ultrafiltration devices (30–50 kDa MWCO), 3,3′,5,5′-tetramethylbenzidine (TMB) liquid substrate system for ELISA, Liberase (Cat. No. 5401119001). Tokyo Chemical Industry (TCI): hydroxymethyl acrylamide (HMA), methoxymethyl acrylamide (MOMAM), FUJIFILM Wako Chemicals: 4,4′-Azobis­(4-cyanovaleric acid) (ACVA), VA-044 initiator SM-102, DSPC, and DMG-PEG_2k_ were purchased from Avanti Polar Lipids (Croda, UK) . ARCA Cy5 eGFP mRNA (5-moUTP modified) was purchased from Stratech. CleanCap EGFP mRNA (5moU) and CleanCap FLuc luciferase mRNA (5moU) were purchased from Tebubio (on behalf of TriLink Biotechnologies). Opti-MEM I Reduced Serum Medium, DAPI, trypsin, penicillin–streptomycin, Flt3-L cytokine (Cat. No. 550704), ACK lysis buffer (Cat. No. A1049201), DNase I (Cat No. AM2222), DNase buffer (Cat. No. AM8170G), and bovine serum albumin (BSA) were purchased from Thermo Fisher Scientific. Fetal bovine serum (FBS) l-glutamine, nonessential amino acids, and β-mercaptoethanol were purchased from Gibco (Thermo Fisher Scientific, UK). One-Glo Luciferase Assay Reagent was purchased from Promega. DiD and DiR lipophilic dyes were purchased from Cayman Chemical. Anti-PEG IgM monoclonal antibody (THE PEG Antibody, mAb, Mouse; Cat. No. A01795–100) was purchased from Invitrogen (Thermo Fisher Scientific). HRP-conjugated goat antimouse IgM secondary antibody (Cat. No. 11839140) was purchased from Merck. High-binding half-area clear 96-well plates were purchased from Scientific Laboratory supplies (on behalf of Corning).

### PAM-Lipid Synthesis—RAFT Polymerization

PAM-lipids were synthesized using commercially available monomers and a synthesized DMG-based macro-CTA (see SI Section 1.2) via reversible addition–fragmentation chain transfer (RAFT) polymerization. Exact conditions for the CTA and polymer synthesis and purification can be found in the Supporting Information (SI Sections 1.2.4–1.2.7). A generalized method is presented here. The monomer (∼200 mg) and the DMG-CTA were dissolved in dioxane/water and dioxane, respectively, and mixed. The final monomer concentration was set to 1.5–2 M. ACVA was added from a 10 mg/mL stock solution in dioxane. The mixture was added to a reaction vial equipped with a magnetic stirrer and sealed with a rubber septum. A sample was taken for NMR at *t* = 0, and the remaining solution was degassed for at least 15 min with nitrogen. The vial was then heated to 70 °C for 18 h. The reaction was then stopped by cooling and exposing the solution to air. The progress of the reaction was checked via ^1^H NMR. If conversion was deemed too low (below ∼80%) fresh ACVA initiator was added to the vial, and the vial was degassed and reheated to 70 °C for 6–24 h. Once satisfactory conversion was achieved, the polymer solution was diluted in the minimum volume needed to reduce viscosity to free-flowing liquid and purified by precipitation into organic solvent (e.g., THF, or THF:diethyl ether 1:1 v/v) and dried in a vacuum desiccator. Dialysis was conducted on redissolved samples against at least 100-fold the volume of deionized water, for at least 36 h, with at least 2 water changes (1 kDa MWCO). Where no suitable precipitation solvent was found, polymers were first dialyzed against at least 100-fold the volume of 2:1 v/v IPA:H_2_O for 12 h, before dialysis against deionized water as above for 36 h. The resulting solution was freeze-dried, and polymers were characterized via ^1^H NMR and GPC. Aliquots of these polymers were taken for replacement of the −S_3_C_5_H_9_ end group with either −H or −OH (methods and characterization detailed in SI Sections 1.2.4–1.2.7). Stock solutions of these polymers were prepared to 1 mM in DMSO and stored at −20 °C.

### Preparation of LNPs

A lipid mix of SM-102:DSPC:Cholesterol:PEG-lipid or PAM-lipid at 50:10:38.5:1.5 molar ratio was prepared in ethanol by mixing stock solutions of lipids (10 mg/mL in ethanol) and PEG/PAM-lipids (1 mM in DMSO). LNPs were prepared by rapid injection of the lipid mix into aqueous mRNA via pipet-mixing.^24^ The aqueous:ethanol v/v ratio was 3:1, with N/P ratio = 6, with a final mRNA concentration of either 25 μg/mL (*in vitro* experiments) or 50 μg/mL (*in vivo* experiments). mRNA encoding either eGFP or luciferase, or poly­(A), was diluted in sodium acetate buffer (50 mM, pH 5.2). The resulting LNP suspension was incubated at room temperature for ∼10 min before being diluted with 1.5-fold the existing concentration of Tris buffer (50 mM, pH 7.4) and stored at 4 °C for a maximum of 24 h before use (unless specified elsewhere). Particle sizes were characterized via DLS after further dilution in Tris buffer (50 mM, pH 7.4) to an mRNA concentration of 2.5 μg/mL. Encapsulation efficiency was determined by the RiboGreen assay, which was performed as per supplier instructions.

#### Buffer Exchange

LNPs for *in vivo* applications were buffer-exchanged and concentrated with either Tris buffer 50 mM (IM, ID injections) or PBS (IV injections) via dialysis and/or centrifugal ultrafiltration (MWCO 30–50 kDa).

#### Fluorescent Dyes

For *in vitro* experiments in THP-1s, DiD dye was incorporated into the ethanol phase at 200:1 molar ratio of lipids:DiD. For *in vivo* biodistribution experiments, DiR dye was incorporated into the ethanol phase at 50:1 molar ratio of lipids:DiR. For *ex vivo* experiments in BMDCs, Cy5-tagged eGFP mRNA was used (Stratech, Cat. No. R1011-APE).

### Subculture of DC2.4 Cell Line

DC2.4 cells (immortalized murine DCs) were thawed and subcultured according to supplier recommendations. Briefly, they were grown in sterile conditions as 2D monolayers in cell culture-culture treated T-75 flasks and kept at 37 °C, 5% CO_2_ (humidified). Cells were grown in ∼10 mL of media comprised of RPMI-1640 (Sigma Cat. No. R0883), 10% FBS (Gibco, Fisher UK), 1X l-Glutamine (Cat. No. TMS-002-C), 1X nonessential amino acids (Cat. No. TMS-001-C), and 0.0054X β-mercaptoethanol (Cat. No. ES-007-E). Cells were passaged at 50–90% confluence (every 1–4 days) at 1:3–1:12 ratios for a maximum of 10 passages. To achieve this, cells were detached from the flask by incubating in 2–3 mL Trypsin (5%) for ∼5–10 min at 37 °C and agitating mechanically. Trypsinization was stopped by the addition of complete media. Cells were centrifuged at 1500 rpm for 5 min to obtain a cell pellet. The supernatant was discarded before resuspension in complete media, and the relevant dilution of cells was added to a new subculture flask containing ∼10 mL of fresh media. Cells were frozen in 1–2 mL complete media +10% DMSO v/v and stored at −80 °C or in liquid nitrogen vapor before use (2–5 × 10^6^ cells per vial).

### *In Vitro* Screening in DC2.4 Cells

DC2.4 cells were seeded at 20,000 cells per well in a white cell culture treated 96-well plate in their regular growth medium. After 23 h, medium was removed and cells were rinsed once with 200 μL of PBS. Next, mRNA-LNPs prepared with either PEG-lipids or PAM-lipids and containing FLuc-mRNA (10 μg/mL, in Tris, 50 mM) were diluted 1:10 to an mRNA concentration of 1 μg/mL in either Opti-MEM (low serum conditions) or Opti-MEM + 10% FBS (high serum conditions) and incubated for 15 min. 100 μL was then added to each well (3–4 wells per condition). Negative controls of no treatment, only Tris buffer (50 mM), and free mRNA were also tested. A positive control of Lipofectamine MessengerMAX was used as per the supplier’s instructions. After 24 h, the treatments were removed and the cells were gently rinsed twice with 200 μL of PBS. 50 μL of One-Glo reagent was then added to each well and analyzed using the One-Glo protocol on the Promega GloMax plate reader. Values presented as fold change are relative to the PEG condition calculated via the following equation.

1 $$\mathrm{Biolum}._{\mathrm{Relative to PEG}}=\frac{\left(\mathrm{Biolum}._{\mathrm{sample}}-\mathrm{Biolum}._{\mathrm{background}}\right)}{\left(\mathrm{Biolum}._{\mathrm{PEG}}-\mathrm{Biolum}._{\mathrm{background}}\right)}$$

where Biolum. = measured bioluminescence of the One-Glo reagent after addition to cells (relative luminescence units, RLU).

### Subculture of THP-1 Cells

THP-1 cells (human monocyte cells) were thawed and subcultured according to supplier recommendations. Briefly, they were grown in sterile conditions in suspension in T-75 flasks and kept at 37 °C, 5% CO_2_ (humidified). Cells were grown in ∼15 mL of media comprised of RPMI-1640 (Sigma Cat. No. R0883), 10% FBS (Gibco, Fisher UK), 1X l-Glutamine (Cat. No. TMS-002-C), 1X nonessential amino acids (Cat. No. TMS-001-C), and 0.0054X β-mercaptoethanol (Cat. No. ES-007-E). Cells were passaged twice per week by diluting one-quarter of the suspension culture in a new flask containing fresh media.

### *In Vitro* Uptake. Stability Studies and Transfection in THP-1 Cell Line

THP-1s were harvested from their growth flasks and centrifuged to a cell pellet to remove growth media before being resuspended to a concentrated stock in Opti-MEM. 250k live cells were then diluted in Opti-MEM and mixed with eGFP-LNPs containing the fluorescent dye DiD to a final volume of 500 μL and a final concentration of mRNA of 1 μg/mL. This suspension was added to a 24-well plate and incubated for at 37 °C, 5% CO_2_ for 24 h. After 24 h, the cells were rinsed with cold PBS, incubated with DAPI (1:1000 in PBS) for 15 min, followed by 3 rinses with 2% FBS in PBS and a final resuspension in 2% FBS in PBS and kept on ice while flow cytometric analysis was conducted.

### Isolation and Culture of BMDCs

Bone marrow (BM) was crushed from C57BL/6 mice femurs and tibias to generate a single-cell suspension in complete (c) RPMI, RPMI1640 supplemented with 10% FCS, 1% glutamine, 1% P–S, 1 mM sodium pyruvate, 1X MEM NEAA, 10 mM HEPES, and 50 μM β-mercaptoethanol. Cells were plated at 2 × 10^6^ cells/well in nontissue-culture-treated 24-well plates with 150 ng/mL Flt3-L at 37 °C, 5% CO_2_. Cells were left to culture for 9 days.

### *In Vitro* Transfection of BMDCs

On the penultimate day of the BMDC culture (day 8), media was carefully removed and replaced with 500 μL per well Opti-MEM I Reduced Serum Medium with 1 μg/mL LNP for 4–6 h. After incubation, 1 mL per well of cRPMI was added and cells were incubated for a further 24 h at 37 °C, 5% CO_2_.

### *In Vivo* Procedures

All animal studies were approved by the University College London Biological Services Ethical Review Committee and performed with UK Home Office approval (PPL 70/8709, PP1692884, and PP4506002). Animal work conformed to the UK Animals (Scientific Procedures) Act, 1986 and Directive 2010/63/EU of the European Parliament. Animals were assigned to experimental groups randomly. Researchers were not blinded to experimental conditions after groups were assigned.

### *In Vivo* Intramuscular (IM) Injection of mRNA-LNPs

LNPs prepared with either PEG-lipids (DMG-PEG_2k_) or PAM-lipids containing FLuc mRNA (2.5 μg, 50 μL, 50 μg/mL) were injected into each hind quadriceps of Balb/c mice. Bioluminescence imaging was performed using an IVIS Spectrum (Revvity UK) at 24 and 48 h. Mice were given anesthesia using 1–2% isoflurane in 100% oxygen and maintained at 37 °C using an integrated heated bed. D-luciferin was administered intraperitoneally (IP) (15 mg/mL, 100 μL, Promega) and images were acquired consistently over a ∼30 min period until bioluminescence peaked (open filter, auto exposure, F/Stop = 2, medium binning) as described elsewhere.^79^ Data in figures/text correspond to the peak bioluminescence recorded. After 48 h, mice were sacrificed and organs harvested and stored at −80 °C. Organs were thawed and submerged in warm D-luciferin solution (15 mg/mL) for 5 min before bioluminescence was measured at 37 °C using the IVIS Spectrum.

### *In Vivo* Intradermal (ID) Injection of mRNA-LNPs

LNPs prepared with either PEG-lipids (DMG-PEG_2k_) or the PAM-lipid DMA_20_-H containing mCherry mRNA (3 μg, 60 μg/mL, 50 μL) were injected into each shaved quadriceps of C57BL/6 mice. After 24, 48, or 72 h, spleen, liver, dLNs, and cLNs were collected and processed for flow cytometry.

### Processing Organs for Flow Cytometry

#### Spleens

Spleens were dissociated through a 70 μm filter in 1 mL PBS using the end of a 1 mL syringe. The filter was washed, and cells were centrifuged in 10 mL of PBS at 400 × *g* for 5 min at room temperature. Splenocytes were then resuspended in 1 mL of ACK lysis buffer for 1 min at room temperature. Ten mL of cRPMI was added, and the cells were counted.

#### Liver

Livers were manually dissected with scissors in 1 mL digestion buffer mix (1X DNase buffer, Liberase 25 μg/mL, 0.625 μg/mL DNase I in PBS) and incubated in 37 °C, 5% CO_2_ for 1 h. Cells were filtered through a 70 μm filter and centrifuged at 400 × *g* for 5 min at 5 °C. Cells were then resuspended in 1 mL of ACK lysis for 1 min at room temperature. Ten mL of cRPMI was added, and the cells were centrifuged at 400 × *g* for 5 min at 5 °C and the supernatant was removed from the cell pellet. Ten mL of cRPMI was added, and cells were counted.

#### Lymph Nodes

LNs were dissociated on ice with needle tips, then resuspended in cold cRPMI, and added to a 70 μm filter. They were then passed through the filter using the end of a 1 mL tip. The filter was washed with 5 mL of cold cRPMI, and the cell suspension was centrifuged at 400 × *g* for 5 min at 5 °C. After the supernatant was removed from the cell pellet, 1 mL of cold cRPMI was added and cells were counted.

#### *In Vivo* Single and Repeated Intravenous (IV) Dosing of mRNA-LNPs

LNPs prepared with either PEG-lipids or the PAM-lipid DMA_20_-H containing FLuc mRNA (5 μg, 50 μg/mL, 100 μL) and DiR dye (50:1 lipid:dye molar ratio) were injected into the tail vein of Balb/c mice. For live whole-body imaging, after 18 h, D-luciferin was injected IP (15 mg/mL, 100 μL) and mice were live imaged using the IVIS Spectrum as described above. For organ biodistribution imaging at 24 h, D-luciferin was injected IP (15 mg/mL, 100 μL), and after 4 min, blood was collected via cervical puncture into EDTA-coated tubes, and organs were harvested and underwent immediate bioluminescence and fluorescence (excitation/emission 745/800) imaging at 37 °C using the IVIS Spectrum.

### Enzyme-Linked Immunosorbent Assay (ELISA) for Antipolymer and Anti-LNP Antibodies

#### Diluted Plasma Isolation

Diluted plasma was isolated from whole blood by diluting 1:2 with PBS and centrifuging for up to 15 min and collecting the supernatant. The supernatant was stored at −80 °C until used.

#### ELISA Protocol

The ELISA protocol was adapted from existing protocols.^67,68^ Low-volume, high-binding clear 96-well plates were coated with PEG, DMA, PEG-LNP, DMA-LNP, or no-polymer–lipid mixes (50 μL, 0.2 mM total lipids, 10 nmol per well, 80:20 Ethanol:DMSO) and left to incubate for 1 h at room temperature to allow insertion/adsorption of lipids into/onto the well surface, followed by three washes with 60 μL of 50 mM Tris buffer (pH 7.5). Wells were blocked for 90 min with 150 μL of 2% (w/v) bovine serum albumin (BSA) in Tris-buffered saline (TBS; 50 mM Tris, 0.145 M NaCl, pH 7.5) and then rinsed once with 0.25% BSA in TBS. Diluted plasma samples were further diluted 1:33.3 in 0.25% BSA in TBS, which results in a 1:100 final plasma dilution due to the prior 1:2 dilution of plasma in PBS. 50 μL of these plasma samples, or primary anti-PEG IgM antibodies (1:2000–1:64,000 serial dilution, 0.25% BSA in TBS, with further dilutions as required) were added to each well and incubated for 1 h at room temperature then removed. Plates were rinsed 3 times with 0.25% BSA in TBS. Wells were then reblocked with 150 μL of 2% BSA in TBS for 30 min, followed by one additional rinse with 0.25% BSA in TBS. 50 μL of horseradish peroxidase (HRP)-linked secondary antibody (1:1000 dilution; 20 μL in 20 mL 0.25% BSA in TBS) was added and incubated for 1 h at room temperature. Plates were then washed six times with 0.25% BSA in TBS prior to detection. The chromogenic substrate 3,3′,5,5′-tetramethylbenzidine (TMB) was added, and absorbance was monitored at 650 nm using a plate reader. The reaction was stopped by the addition of 2 N H_2_SO_4_ after a maximum of 5 min, or earlier when a maximum optical density approaching 0.4 was reached at 650 nm. The optical density at 450 nm (OD450) was then measured using a plate reader.

### Conventional Flow Cytometry

Cells were collected into FACS tubes and incubated 30 min on ice in antibody cocktails including Fixable Viability Dye eFluor 680 (eBiosciences), in a total of 100 μL per sample of FACS buffer. Cells were subsequently washed with FACS buffer. Cells were acquired using the BD LSRFortessa analyzer. All antibodies used are listed in Table S6.

### Spectral Flow Cytometry

Cells were distributed into a 96-well V-bottom plate and incubated in TruStain FcX to block Fc receptors for 20 min on ice. Fixable Viability Dye eFluor 680 (eBiosciences) was subsequently added to the cells for 10 min. Cells were washed with FACS buffer (2% FBS, 2 mM EDTA, and PBS) before adding antibody cocktails that were prepared in a total volume of 50 μL per sample in Brilliant Stain Buffer (BD Biosciences) and FACS buffer. Cells were incubated with antibodies for 30 min on ice and then washed with FACS buffer. Cells were then acquired on a NovoCyte Opteon spectral flow cytometer. All antibodies used are listed in Table S6.

### NMR Spectroscopy

All ^1^H NMR spectra were recorded in ppm (δ) at 400 MHz in DMSO-d6 using a Bruker Avance III NMR spectrometer that was maintained at 25 °C. To analyze the spectra, MestReNova 6.0.2 Copyright 2009 (Mestrelab Research S.L.) was applied.

### Theoretical Molar Mass Calculation

The theoretical number-average molar mass (*M*_*n*,th_) was calculated as demonstrated in the following way:

2 $$M_{n,th}=\frac{{[M]}_{0}pM_{M}}{{[CTA]}_{0}}+M_{CTA}$$

Eq 2 is for the theoretical number-average molecular weight (*M*_*n*,th_). [*M*]_0_ and [CTA]_0_ are the initial concentrations (in M) of the monomer and chain transfer agent, respectively. *p* is the fractional monomer conversion as determined by ^1^H NMR spectroscopy. *M*_M_ and *M*_CTA_ are the molecular weights (g mol^–1^) of the monomer and chain transfer agent, respectively.

### Gel Permeation Chromatography

The gel permeation chromatography (GPC) analysis was performed using a Shimadzu I-series GPC system equipped with a UV/vis detector (wavelength set to 309 nm) and a differential refractive index (DRI) detector. The system was equipped with a PLgel 5 μm guard column and a 2 × PLgel Mixed-D columns (300 × 7.5 mm). Dimethylformamide (DMF) with 0.1% LiBr was utilized as an eluent with a flow rate of 1 mL/min at 50 °C. The instrument was calibrated through Poly­(methyl methacrylate) standards (Agilent EasyVials) between 550 and 955500 g mol^–1^. Using conventional calibration, in Cirrus GPC software, dispersity (*Đ*), molecular weight (*M*_w_), and experimental molar mass (*M*_*n*,GPC_) were detected.

### Dynamic Light Scattering

Dynamic light scattering (DLS) for 30 PAM-LNP formulations, PEG-LNP formulation, and PEG-free LNP formulation was conducted with a DynaPro NanoStar Plate reader (Wyatt Technology) at 25 °C to characterize the particles in terms of the size and PDI.

For all other dynamic light scattering (DLS) results, a Zetasizer Ultra (Malvern, Inc.) instrument was used to characterize the particles in terms of size, PDI, at 25 °C, scattering angle 173°.

### Small-Angle X-ray Scattering

SAXS measurements of LNPs ([LNP] ≈ 30 mg/mL in 50 mM Tris-HCl pH = 7) were performed on the Nano-inXider (Xenocs, Grenoble, France) using a microfocus sealed tube Cu 30*W*/30 mm X-ray source with λ = 1.54 Å and two Dectris Pilatus 3 hybrid photon counting detectors. Samples were loaded and sealed in borosilicate glass capillary tubes of 1.0 mm thickness, which were mounted on a sample holder. Data were collected over a scattering wavevector *q* = 4π/λsin­(θ/2) range of 0.0045–0.37 Å^–1^, where θ is the scattering angle. Samples and their corresponding buffer were measured at room temperature. The 2D scattering pattern was radially averaged, and the background buffer was subtracted using Foxtrot software (SWING | French national synchrotron facility, https://www.synchrotron-soleil.fr/en/beamlines/swing).

### Small-Angle Neutron Scattering

SANS measurements of LNPs were performed on the SANS2D beamline at the ISIS Neutron and Muon Source (Rutherford Appleton Laboratory, Oxfordshire, UK). The instrument configuration used a 12 m collimation, neutrons with a wavelength range of 1.75–12.5 Å in time-of-flight mode, and two detectors placed 5 and 12 m from the sample, respectively, giving a *q*-range of 0.0015–0.5 Å^–1^ in 2 mm thick quartz cuvettes mounted in a temperature-controlled sample changer. Samples and their corresponding buffers were measured at 25 °C and [LNP] ≈ 3 mg/mL in 50 mM Tris-DCl with 100% D_2_O (pD 7.4).

Raw 2D data were corrected (incident neutron wavelength distribution, detector efficiency, measured transmission, and sample volume), radially averaged, scaled to absolute intensity, merged, and background-subtracted using Mantid Workbench.^80,81^ The 1D reduced data correspond to the coherent elastic differential scattering cross-section [∂Σ/∂Ω­(*q*)], commonly referred to as the intensity, *I* [cm^–1^].

### Small-Angle Neutron/X-ray Scattering Data Analysis

All the SANS/SAXS data were analyzed using SasView (version 6, https://www.sasview.org/) for model fitting and BioXTAS RAW 2.3.1 (https://bioxtas-raw.readthedocs.io/en/latest/) for shape-independent fitting (inverse Fourier transform and Guinier approximation).

SAXS data were fitted, where appropriate, using the broad peak model in SasView to infer the internal structure of the LNPs. The pair distance distribution function *P*(*r*) analysis was performed on all SAS data using the RAW^82^ implementation of the indirect Fourier transform method, GNOM,^83^ to determine the maximum dimension (*D*_max_) and the radius of gyration (*R*_g_) and to estimate the shape of the particles. The DENSS algorithm^58^ was then used to calculate the ab initio density map directly from the GNOM output and to reconstruct the three-dimensional structure. The *D*_max_ and *R*_g_ values from SAXS were consistently lower than those from SANS due to the limited q-range accessed with SAXS. Guinier analysis was also performed on the SANS data, and the *R*_g_ values determined were compared to the ones derived from *P*(*r*). The SANS data were then fitted using SasView with a core–shell ellipsoid model (CSE) and a cylinder model with a core and two shells. The core is assumed to contain the RNA and most of the SM-102 and cholesterol, the first shell is made up of DSPC and DMG, and the second shell represents the PEG layer. The core radius, shell thickness, and SLD of the shells were allowed to vary with all other parameters estimated and fixed. The fittings were optimized using the DREAM fit algorithm.

### Cryo-Electron Microscopy

mRNA-LNP samples (1.5 mg/mL of total lipids; eGFP mRNA) were prepared for cryo-electron microscopy (cryo-EM) using a GP2 plunge freezer (Leica Microsystems). Three μL of LNP was applied to glow-discharged 300-mesh copper grids with a lacey carbon support film (Agar Scientific). Grid preparation was performed at 4 °C and 85% relative humidity. After application of the sample, the instrument was programmed with a 40 s wait time, followed by a 7 s front blot and 0 s drain time prior to vitrification. Grids were rapidly plunged into liquid ethane at −183 °C, then transferred to liquid nitrogen for storage.

Grids were clipped and loaded into a Titan Krios G3i transmission electron microscope (Thermo Fisher Scientific) equipped with a K3 direct electron detector and a BioQuantum energy filter (Gatan). Data were collected in counting mode with a total electron dose of 85.4 e^–^/Å^2^, a nominal magnification of 105,000×, a defocus of −3 μm, and a 20 eV energy slit width. Grids were first screened for ice thickness and particle distribution. Integrated images were saved as 2×-binned JPEG files, corresponding to a pixel size of 1.6712 Å.

### Statistics

Statistics were calculated using GraphPad Prism software (versions 10.6.0 and 10.6.1).

## Supplementary Material



## Data Availability

All relevant data can be obtained upon request from the corresponding authors at b.fiedler@ucl.ac.uk and p.gurnani@ucl.ac.uk. SANS data can be found at http://10.5286/ISIS.E.RB2510742-1.

## Notes

Encapsulation efficiency and cytotoxicity of PAM-LNPs and PEG-LNPs; effect of polymer end group on mRNA-LNP transfection in DC2.4 cells; association, transfection, and activation of primary BMDCs; SANS and SAXS data, model fitting parameters, and structural analysis of PAM-LNPs and PEG-LNPs; supporting *in vivo* biodistribution, luciferase expression, and antipolymer antibody data; structures of PEG, PAMs, and other PEG-alternative polymers; supporting materials and chemical synthesis of RAFT agents and PAM-lipids; polymer synthesis conditions and characterization; flow cytometry gating strategies and antibody panels; NMR spectra of RAFT agents and PAM-lipid polymers (PDF)
