# Supplementary material for: Poly(acrylamido) PEG-Alternative Lipids Rescue mRNA Efficacy during Repeated Dosing and Enhance Immune Cell Delivery
Source: ACS Nano. 2026 Oct 22;20(43):29015–32. doi: 10.1021/acsnano.6c11940 (PMC13738739; doi:10.1021/acsnano.6c11940)
Supplement: Supplementary file 1 [file nn6c11940_si_001.pdf]

*Supporting Information for:*

# Poly(acrylamido) PEG-alternative Lipids Rescue mRNA Efficacy During Repeated Dosing and Enhance Immune Cell Delivery

Benjamin M. Fiedler[a*], Charlotte Galley[b,†], Margarita Strimaite[a,c,†], Nga Man Cheng[d], Najet Mahmoudi[e], Zhiping Feng[c], Tammy L. Kalber[c], Maria-Jose Martinez-Bravo[a], Christopher J. Morris[a], Jenny K. W. Lam[a], Daniel J. Stuckey[c], Gareth R. Williams[a], Clare L. Bennett[b] and Pratik Gurnani[a*]

[a] UCL School of Pharmacy, University College London, London, WC1N 1AX

[b] UCL Cancer Institute, Department of Haematology, University College London, London, WC1E 6DD

[c] UCL Centre for Advanced Biomedical Imaging, University College London, London, WC1E 6DD

[d] School of Pharmacy, The University of Nottingham, Nottingham, NG7 2TQ

[e] ISIS Neutron and Muon Source, Rutherford Appleton Laboratory, Didcot OX11 0DE

† These authors contributed equally to the publication

# Supplementary information

## Supporting data

### Characterisation and screening of library of 30 PAM-LNPs

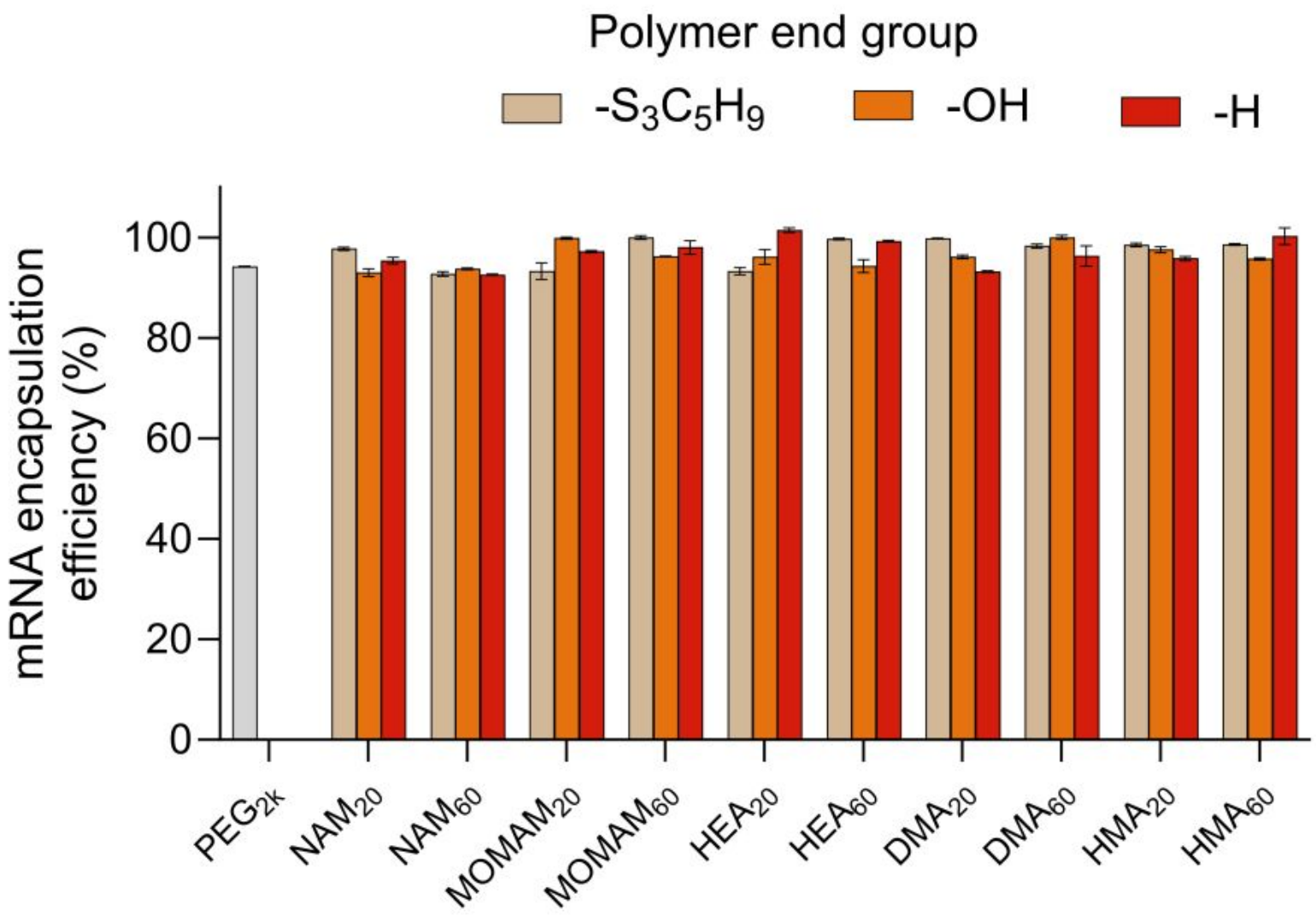


**Figure S1. Encapsulation efficiency of PEG-LNPs and PAM-LNPs** Determined by ribogreen assay (following manufacturers protocols). Data presented as mean of 3 technical replicates. Data presented as mean. Error bars represent ± s.d.

### Cytotoxicity of PAM-LNPS and PEG-LNPs

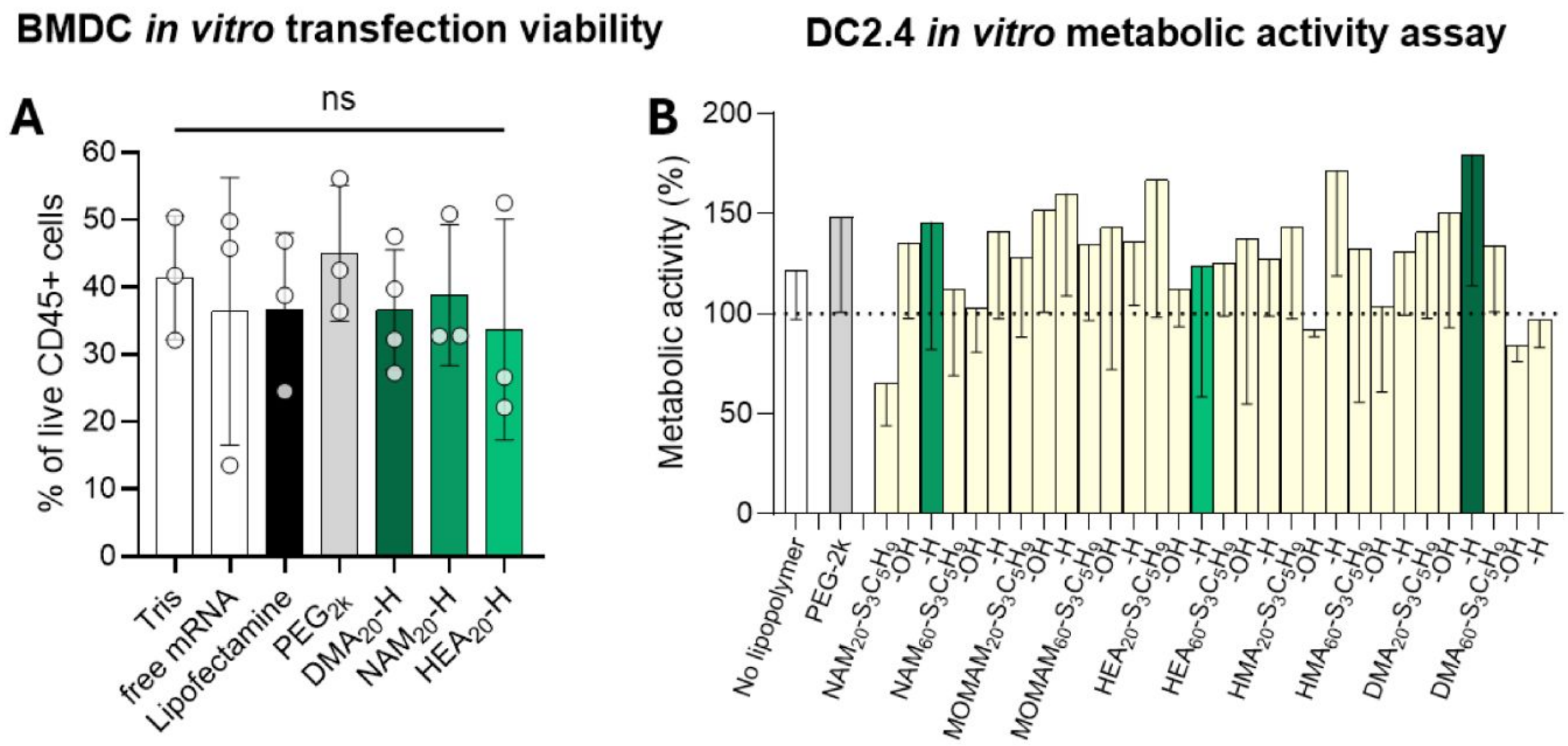


**Figure S2. PAM-LNPs are not cytotoxic and are well tolerated by immune cells. A** percentage of live CD45+ cells in BMDC cultures treated with vehicle control (tris), controls, or LNP formulations prepared with PEG-lipids or PAM-lipids for 24 hours ahead of analysis via flow cytometry. Data presented as mean biological repeats (n=3-4) ± s.d.. Statistical significance was determined by one way ANOVA with Dunnett correction. **B** Formulation of PAM-LNPs from DMG-based PAM-lipids with varied chemistries (5 monomers, 2 end-groups and 2 molar masses) and control PEG-LNPs, were incubated with DC2.4 cell line for 24 hours. Metabolic activity was determined via

PrestoBlue assay. Data presented as % of metabolic activity relative to cells treated with vehicle control (Tris buffer) and kill condition (1% Triton X-100) as the mean of *n* = 3 biological repeats, error bars represent s.d..

## Effect of polymer end-group on mRNA-LNP transfection in DC2.4s

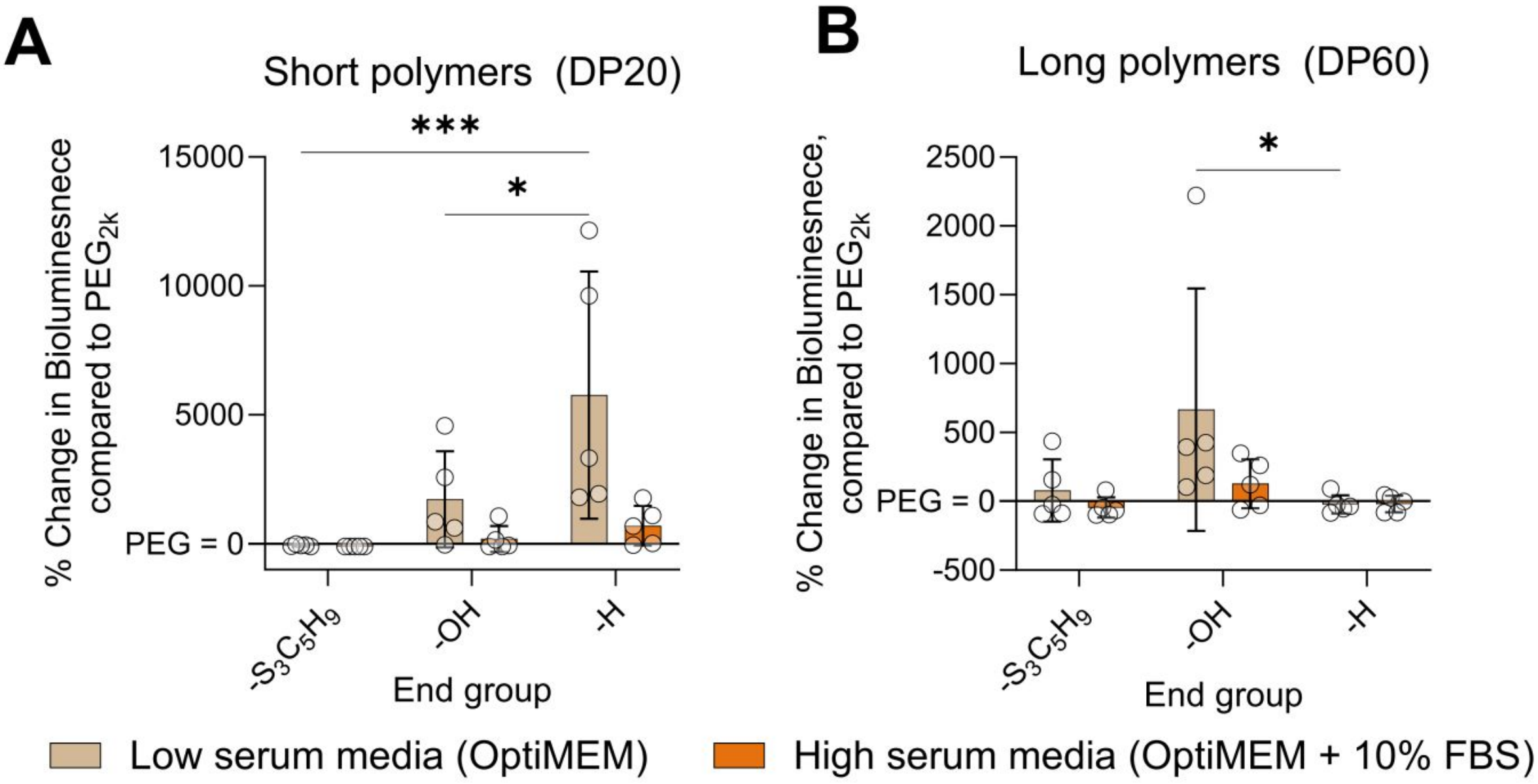


**Figure S3. End-group removal of PAM-lipids improves transfection of mRNA-LNPs more for shorter DP20 polymers than for longer DP60 PAMS (relative to PEG-LNPs).** Change in bioluminescence of DC2.4 cells after 24 hour incubation with PAM-LNPs prepared with either short (**A**) or long (**B**) PAM-lipids. Each datapoint represents the mean of 3 biological repeats for LNPs prepared with a specific polymer with a different monomer/backbone chemistry to others within the group. Data presented as mean. Error bars represent ± s.d. Significance determined by two-way ANOVA with Tukey correction. *$P < 0.05$, **$P < 0.01$., ***$P<0.001$.

## PAM-LNPs and PEG-LNPS in primary BMDCS

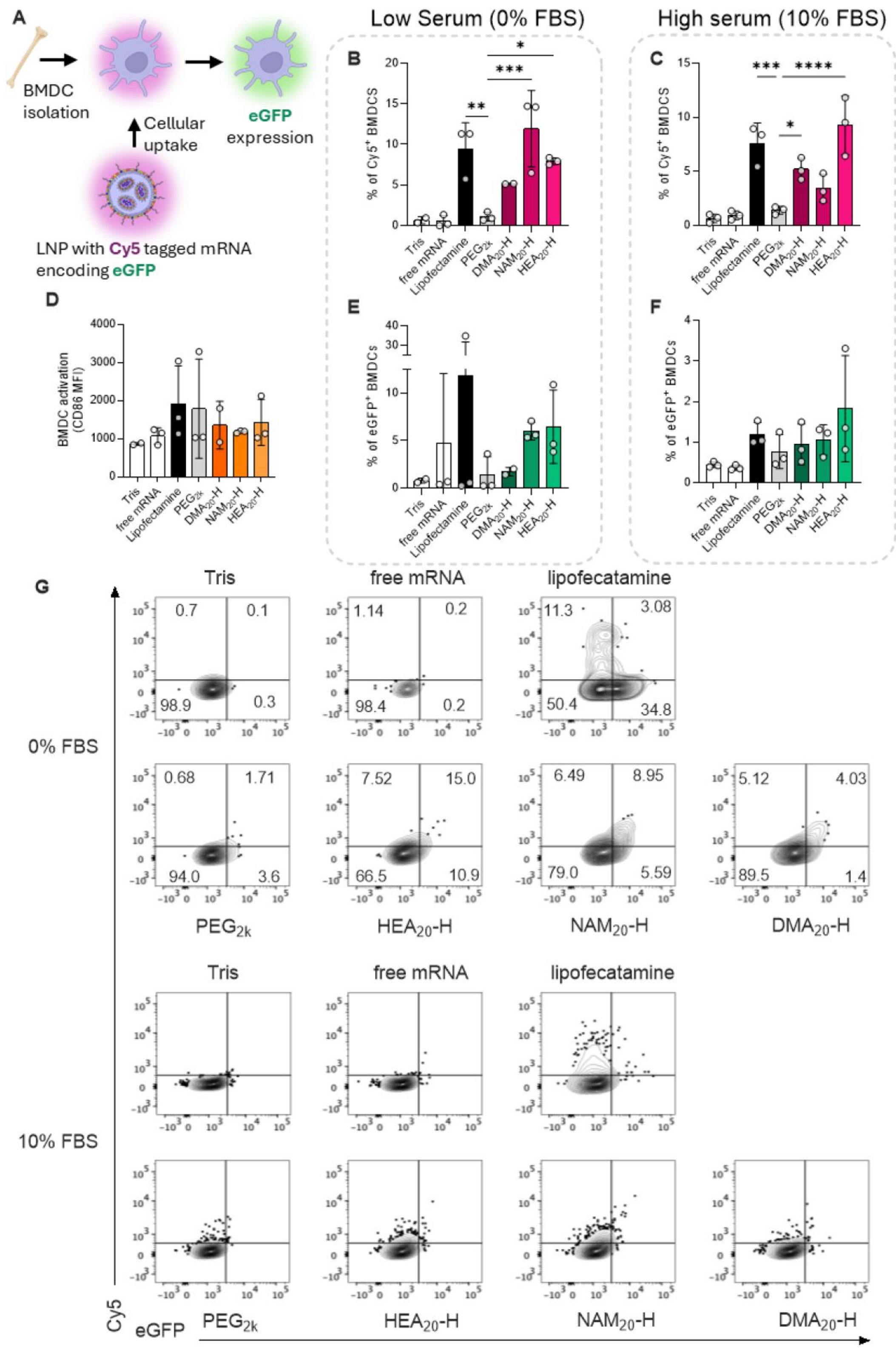


**Figure S4. PAM-LNPs show superior association/internalisation in BMDCs than PEG-LNPs PAM-LNPs show superior association/internalisation in BMDCs than PEG-LNPs. (A)** Schematic representation of experiment. BMDCs transfected with LNPs with Cy5 tagged mRNA encoding eGFP. Internalisation (Cy5+ frequency) in low **(B)** and high serum **(C)** of BMDCs by PAM-LNPS or PEG-LNPs containing Cy5-tagged mRNA encoding eGFP, and corresponding BMDC activation as measured by CD86 **(D)**. Transfection (eGFP+ frequency) in low **(E)** and high serum **(F)** of BMDCs by PAM-LNPS or PEG-LNPs containing Cy5-tagged mRNA encoding

eGFP. Representative flow cytometry plots of eGFP (X axis) Cy5 (Y axis). Data presented as mean of biological repeats (n=3 for all samples, except Tris and $DMA_{20}$-H where n=2) ± s.d.. Significance determined by one way ANOVA with Dunnett correction. *$P$ < 0.05, **$P$ < 0.01., ***$P$<0.001.

PAM-LNPs, and to a lesser extent PEG-LNPs, show a general population shift from $Cy5^-$/$eGFP^-$ to $Cy5^+$/$eGFP^+$, suggesting a correlation between mRNA uptake/association and eGFP expression. Interestingly these behave differently to lipoplex Lipofectamine, where the cell population bifurcates into $Cy5^+$/$eGFP^-$ and $Cy5^-$/$eGFP^+$. Improvement in eGFP expression by PAM-LNPs compared to PEG-LNPs is not statistically significant, which appears in contrast to the data in figure 1 showing significant improvements when using eGFP-mRNA, rather than Cy5-tagged mRNA. This is likely due to our observation that Cy5-tagged mRNA does not transfect as efficiently as unlabelled mRNA (confirmed by manufacturer).

## SANS and SAXS analysis of PAM-LNPs and PEG-LNPs

**Table S1.** Maximum dimension ($D_{max}$), radius of gyration ($R_g$), and shape factor of the LNP samples calculated using GNOM IFT on the SANS data. The shape factor is calculated with $D_{max}/R_g$ from P(r) analysis where the shape factor of a sphere is 2.58 and a prolate ellipsoid is 3.[1]

| Sample | $D_{max}$ | $R_g$ (Å) | $\chi^2$ | Shape factor |
|---|---|---|---|---|
| $DMA_{20}$-H | 1575 | 490.7 ± 3.02 | 0.9253 | 3.21 |
| $HEA_{20}$-H | 1584 | 480.4 ± 4.13 | 0.8516 | 3.30 |
| $NAM_{20}$-H | 1556 | 500.4 ± 2.09 | 0.8885 | 3.11 |
| $PEG_{2k}$ | 1343 | 426.1 ± 2.20 | 0.9638 | 3.15 |

**Table S2.** Radius of gyration (Rg) estimated using Guinier analysis on the SANS data with BioXTAS RAW 2.1.1.

| Sample | Rg (Å) | $r^2$ fit | q min*Rg | q max*Rg |
|---|---|---|---|---|
| $DMA_{20}$-H | 573.83 ± 21.59 | 0.9480 | 1.0558 | 1.4977 |
| $HEA_{20}$-H | 510.88 ± 13.79 | 0.9078 | 0.7868 | 1.4969 |
| $NAM_{20}$-H | 659.30 ± 50.07 | 0.9376 | 1.0153 | 1.4439 |
| $PEG_{2k}$ | 477.47 ± 10.09 | 0.9934 | 0.8785 | 1.4849 |

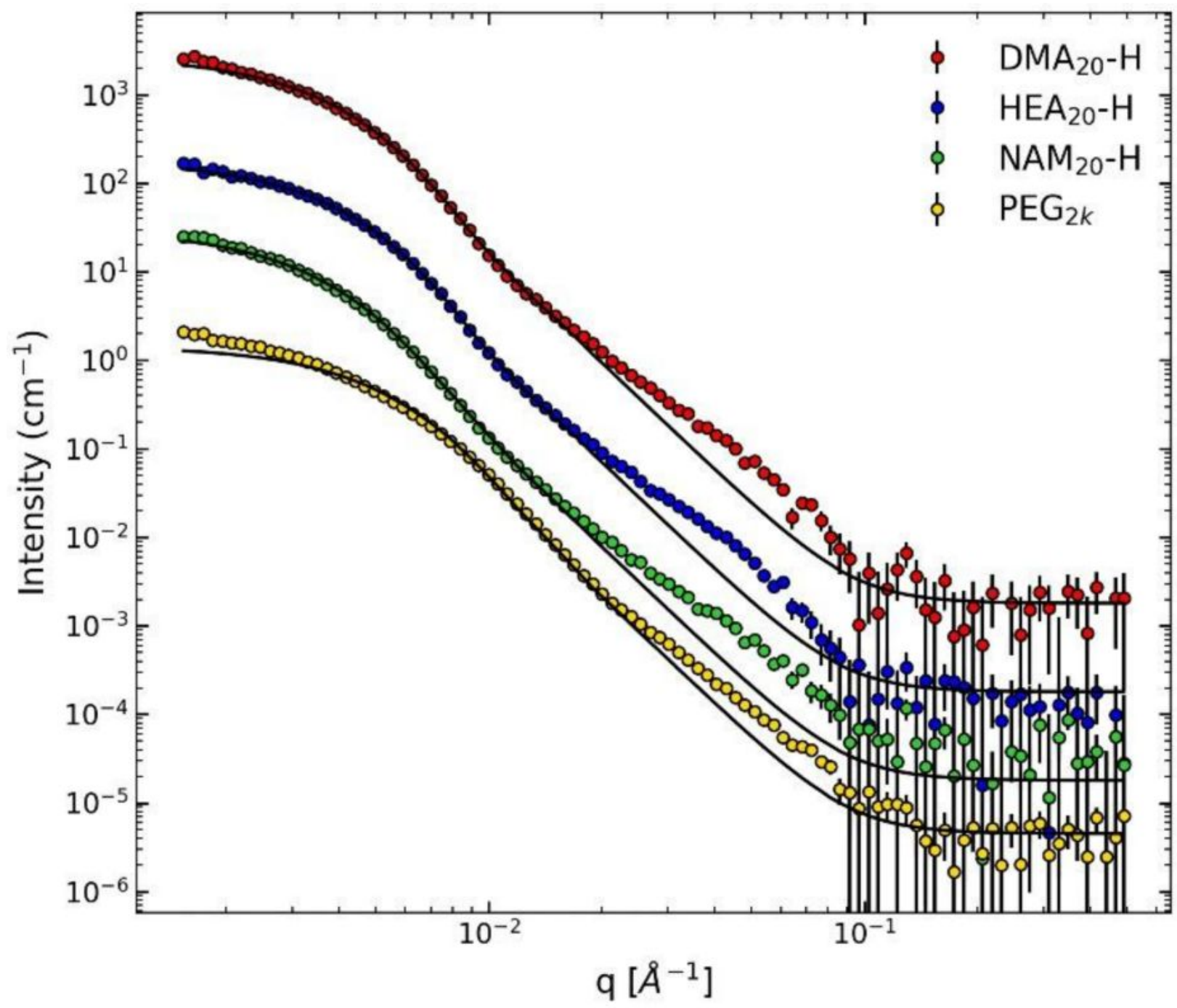


**Figure S5.** SANS data of LNPs prepared with PEG2k, DMA20-H, NAM20-H, or HEA20-H fitted using core-shell ellipsoid model with SasView and DREAM algorithm.

**Table S3**. Fitted parameters and $\chi^2$ from SANS using core-shell ellipsoid model with SasView and DREAM algorithm.

| **Sample** | **Core radius** | **x core** | **Shell thickness** | **SLD core** | **SLD shell** | **Core PDI** | **Shell PDI** | $\chi^2$ |
|---|---|---|---|---|---|---|---|---|
| $DMA_{20}$-H | 338.86 ± 8.10 | 2.00 ± 0.07 | 21.39 ± 4.05 | 0.72 ± 0.08 | 6.19 ± 0.23 | 0.29 ± 0.02 | 0.02 ± 0.12 | 29.18 |
| $HEA_{20}$-H | 334.55 ± 10.0 | 1.94 ± 0.19 | 20.14 ± 10.22 | 1.54 ± 0.18 | 6.18 ± 0.24 | 0.28 ± 0.03 | 0.02 ± 0.12 | 20.23 |
| $NAM_{20}$-H | 327.53 ± 11.2 | 1.98 ± 0.10 | 20.36 ± 5.86 | 1.12 ± 0.11 | 6.19 ± 0.31 | 0.36 ± 0.02 | 0.17 ± 0.13 | 26.20 |
| $PEG_{2k}$ | 201.39 ± 3.73 | 2.00 ± 0.03 | 20.02 ± 1.81 | 0.60 ± 0.06 | 6.16 ± 0.23 | 0.40 ± 0.01 | 0.21 ± 0.13 | 60.72 |

**Table S4.** Fitted parameters and $\chi^2$ from SANS fitting using core-two-shell cylinder model with SasView and DREAM algorithm.

| Sample | Core radius | Core length | Shell thickness 1 | Shell thickness 2 | SLD core | SLD shell 1 | SLD shell 2 | Core PDI | Shell 1 PDI | Shell 2 PDI | $\chi^2$ |
|---|---|---|---|---|---|---|---|---|---|---|---|
| DMA | 546.23 ± 4.93 | 465.13 ± 4.55 | 52.44 ± 1.65 | 34.37 ± 10.35 | 1.32 ± 0.01 | 0.12 ± 0.02 | 4.49 ± 1.08 | 0.40 ± 0.01 | 0.33 ± 0.14 | 0.40 ± 0.07 | 8.16 |
| HEA | 515.25 ± 5.74 | 482.77 ± 5.14 | 40.14 ± 1.03 | 32.29 ± 10.33 | 1.93 ± 0.02 | 0.20 ± 0.08 | 4.68 ± 1.10 | 0.40 ± 0.01 | 0.34 ± 0.13 | 0.39 ± 0.14 | 6.45 |
| NAM | 600.59 ± 7.16 | 498.14 ± 5.77 | 47.33 ± 1.88 | 42.61 ± 10.16 | 1.74 ± 0.02 | 0.12 ± 0.03 | 4.21 ± 1.07 | 0.40 ± 0.004 | 0.37 ± 0.11 | 0.39 ± 0.05 | 6.63 |
| PEG | 482.08 ± 3.01 | 257.32 ± 2.67 | 40.83 ± 1.22 | 37.80 ± 10.42 | 1.34 ± 0.01 | 0.12 ± 0.001 | 4.14 ± 1.14 | 0.40 ± 0.001 | 0.40 ± 0.01 | 0.40 ± 0.005 | 23.61 |

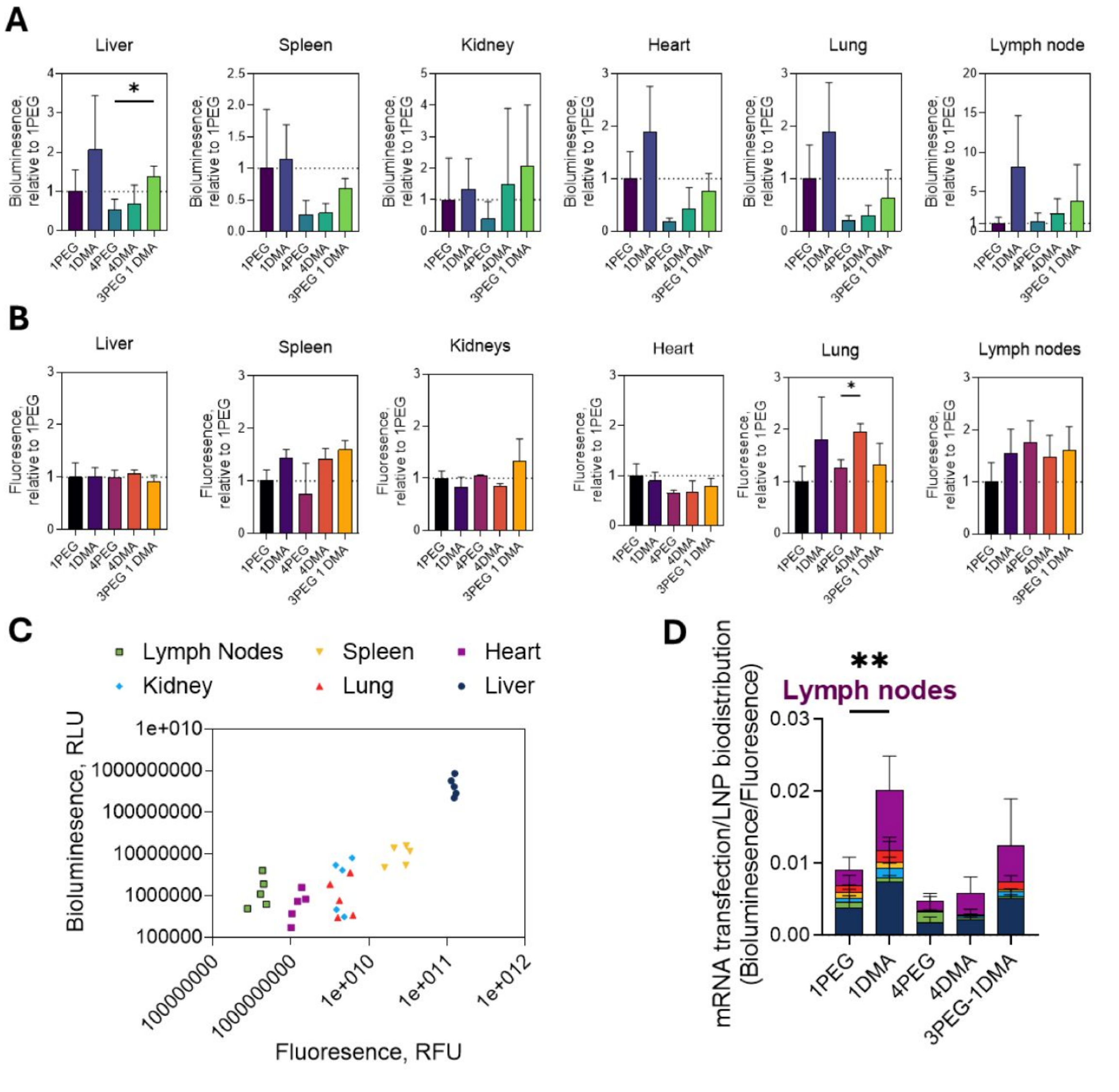


**Figure S6. Accompanies figure 5. PAM-lipids effectively replace PEG-lipids during repeat dosing of mRNA-LNPs** Bioluminescence (**A**) and fluorescence (**B**) of FLuc mRNA LNPs (5 ug of mRNA per dose) prepared with PAM-lipid $DMA_{20}$-H or PEG-lipid $PEG_{2k}$. Data presented for each organ as value relative to a single dose of PEG-LNP (1PEG condition). Organs and blood collected 24 h after final dose (either 1 dose or 4 doses) and imaged using in vivo imaging system (IVIS) to quantify bioluminescence (**A**) and fluorescence (**B**) from FLuc mRNA expression and fluorescently tagged LNP (DiR) respectively. Data presented as mean of biological repeats ± s.e.m. (n=3). Statistical significance was determined by one way ANOVA (Welch) with Dunnett correction compared against either 1PEG (1DMA) or 4PEG (4DMA, 3PEG-1DMA) condition, *P < 0.05. (**C**) shows corresponding bioluminescence and fluorescence values for each formulation, as the mean of 3 biological repeats. (**D**) shows

mRNA transfection (bioluminescence) normalised against organ accumulation (fluorescence) of mRNA-LNPs. Data presented as mean of biological repeats (n=3). Statistical significance determined by two way ANOVA with Dunnett correction, compared against either 1PEG (1DMA) or 4PEG (4DMA, 3PEG-1DMA) condition, *P < 0.05, **P<0.01.

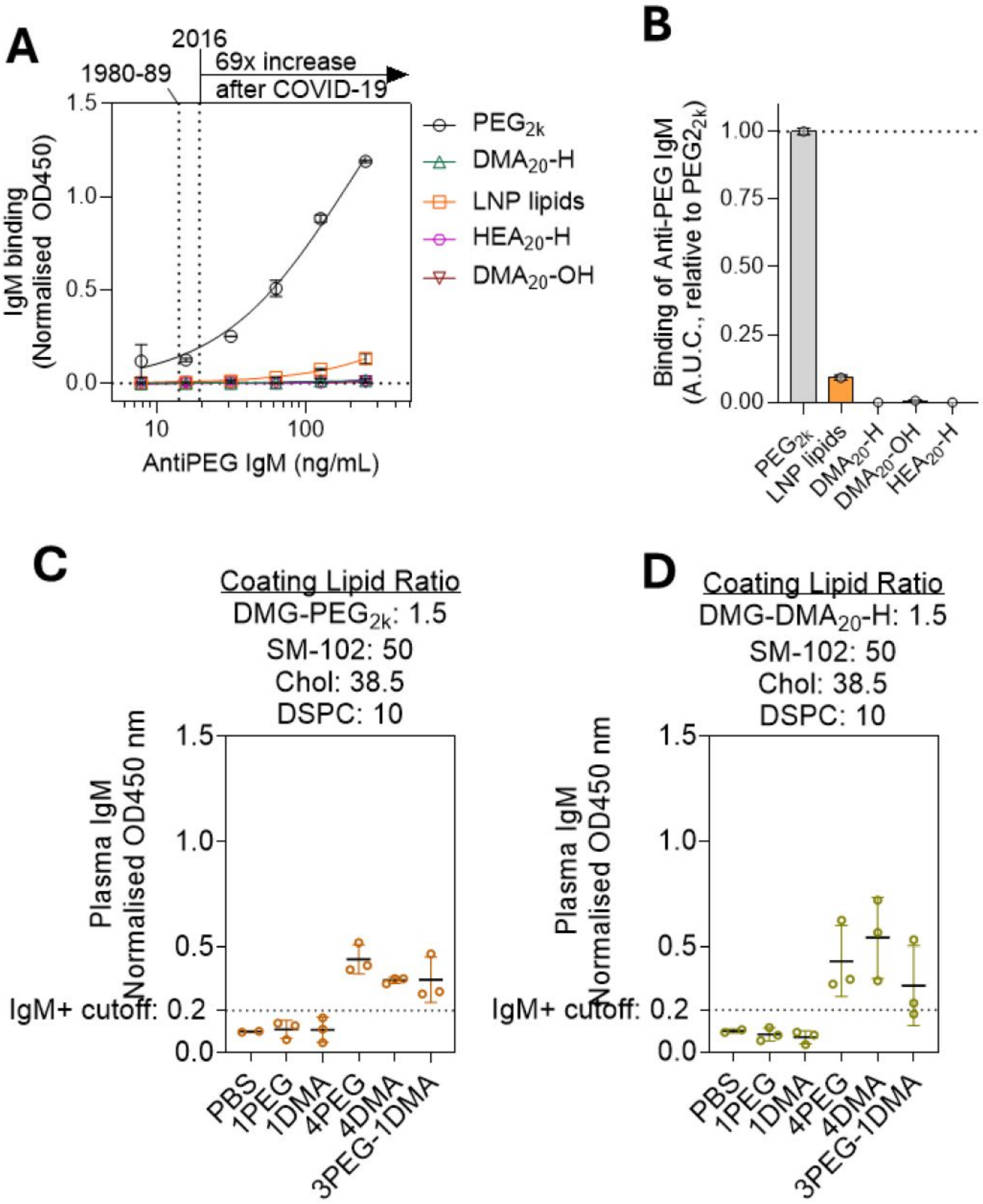


**Figure S7. Accompanies figure 5 A**) Binding of commercially available murine anti-PEG IgM to $PEG_{2k}$, $DMA_{20}$-H, $HEA_{20}$-H and $DMA_{20}$-OH or non-polymer LNP components SM102, Cholesterol, and DSPC mixed in 50:38.5:10 molar ratios. Dotted lines represent clinically determined anti-PEG IgM levels from specified years before COVID-19, and fold-increase after COVID-19 vaccination[2,3]. **B)** Total IgM binding of commercially available anti-PEG IgM determined by area under curve (AUC) integration analysis of figure, normalised to $PEG_{2k}$. **C)** and **D**) Plasma IgM detected via enzyme-linked immunosorption assay (ELISA) of plates coated with specified polymer-lipids/lipids (LNP components). Data was normalised to OD450 nm of PBS control, set to OD450=0.1. $IgM^{+}$ Cutoff set as 2x OD450nm of PBS control. Data presented as mean of biological repeats ± S.D. (n=3)

**Structures of PEG, PAMs, and other common PEG alternatives**

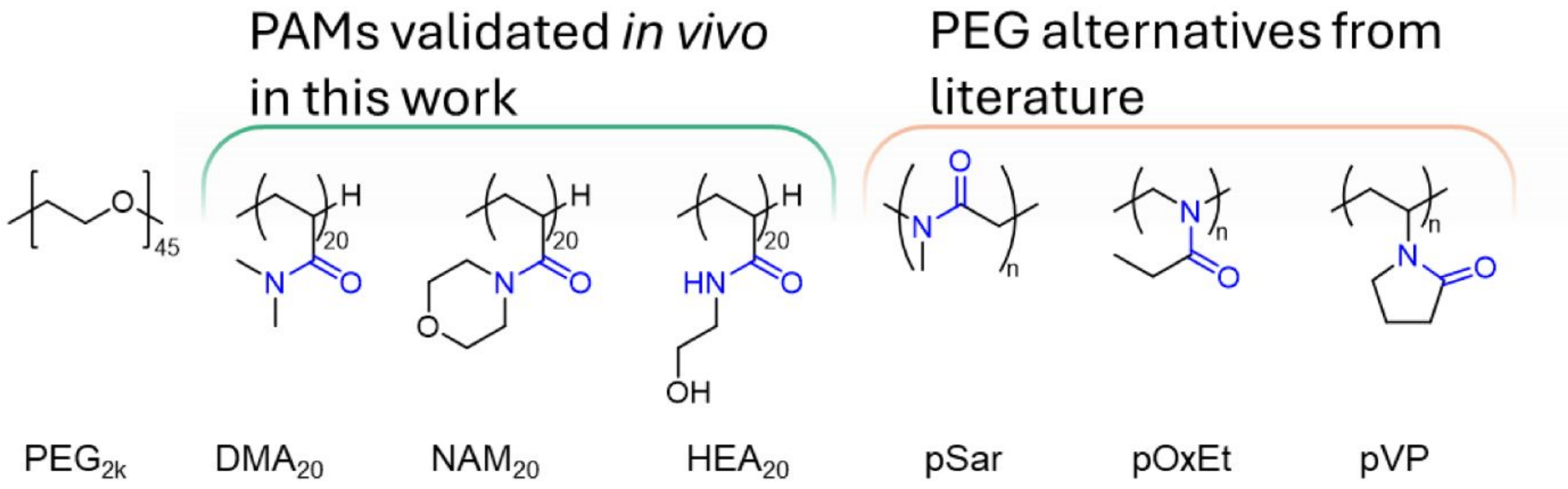


**Figure S8.** Structures of PEG, poly(acrylamido) (PAM) polymers, and PEG alternative polymers from literature. Substituted amide groups highlighted in blue.

# Supporting materials and methods

## Materials

**Sigma-Aldrich (Merck)**: All solvents, solketal, 4-dimethylaminopyridine (DMAP), EDC·HCl (1-ethyl-3-(3-dimethylaminopropyl)carbodiimide hydrochloride), myristic acid (tetradecanoic acid), ammonium bromide ($NH_4Br$), sodium sulfate ($Na_2SO_4$), hydrogen peroxide (30% w/w aqueous solution), 1-ethylpiperidine hypophosphite (EPHP), deuterated NMR solvents Silica for column chromatography and silica gel 60 TLC aluminium sheets (4 cm × 8 cm) with fluorescent indicator (254 nm) for TLC. **FUJIFILM Wako Chemicals:** VA-044 initiator (2,2′-azobis[2-(2-imidazolin-2-yl)propane] dihydrochloride). **Thermo Fisher Scientific:** Amberlyst™ 15 hydrogen form ($H^+$)

## Chemical synthesis

A DMG-based macro-CTA DMG-PABTC was synthesised via a new route adapted from other methods[4,5]

### Synthesis of Solketal-PABTC

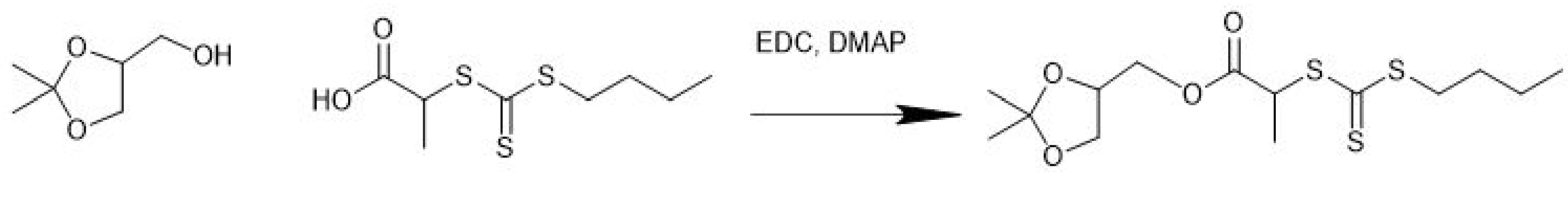


**Schematic S1.** Synthesis of solketal-PABTC

2-((butylthio)-carbonothioyl) thio propanoic acid (PABTC) was synthesized via previously reported methods[6]. PABTC (1 g, 4.02 mmol ) and Solketal (542 mg, 576 µL, 4.1 mmol) were dissolved in 10 mL dichloromethane (DCM) and cooled to 0 °C in a round bottom flask equipped with a magnetic stirrer bar. A solution of DMAP (219 mg, 1.8 mmol) dissolved in 5 mL of DCM was added slowly with stirring, and left for 5 minutes. A solution of EDC-HCl (886 mg, 4.62 mmol) in DCM (5 mL) was added dropwise with stirring and left to react overnight, allowing the ice bath melt and reach room temperature. A deep red colour was first observed which returned to bright yellow/orange overnight. Reaction progress was monitored to

satisfactory completion with thin layer chromatography (TLC) using ethyl acetate (0-40%) in hexane . The resulting reaction mixture was diluted with ~25 mL of chloroform then washed twice with roughly equal volumes of $NH_4Br$ (0.5 M), then washed twice with roughly equal volumes of brine, then dried over $Na_2SO_4$. The product was purified via column chromatography (0-15% Ethyl acetate in hexane, gradient elution). The product fractions were concentrated via rotary evaporation to a viscous yellow oil and characterised by $^1$H NMR.

## Synthesis of Glycerol-PABTC

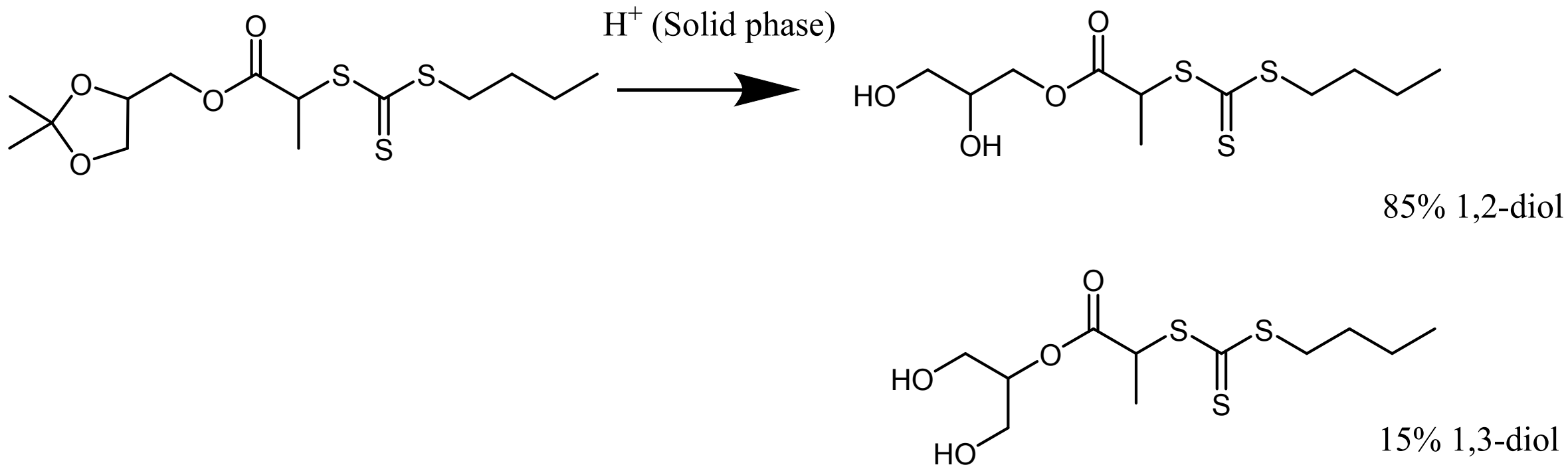


**Schematic S2. Synthesis of Glycerol-PABTC**

Method adapted from literature[7]. $H^+$ amberlyst resin (300 mg) was added to a round bottom flask equipped with magnetic stirrer bar. Solketal-PABTC (500 mg) was dissolved in 15 mL methanol and added the flask. The solution was set heated to 60 °C and was monitored to satisfactory completion (~20 hours) via TLC (0-40% ethyl acetate in hexane). The product was separated from the amberlyst with filtration paper and the crude reaction mixture was then purified via column chromatography (0-40% Ethyl acetate in hexane, gradient elution). The product fractions were concentrated via rotary evaporation to a viscous yellow oil and characterised by $^1$H NMR.

At this stage it was determined *via* $^1$H NMR that ~15% of the product was the 1,3-diol isomer, rather than the 1,2-diol isomer (Schematic S2), which was consistent between batches. This has been seen in other reports of acid-mediated deprotection of solketal to glycerol-like molecules[4,8]. This is due to isomerisation during this reaction step. Regardless, the mixed product of 1,2- and 1,3- glycerol-PABTC was deemed acceptable and used without isomeric purification due to: 1) the potential difficulty of separating these isomers from each-other; and 2) we assumed this would have little impact on final properties of polymer-lipid; and 3) commercially available DMG-$PEG_{2k}$ is ~3% 1,3- isomer (Avanti Research, DMG-PEG 2000, product no. 80151). Moreover, no evidence of the 1,3 isomer was seen in the final product (DMG-PABTC) – potentially due to further purification.

## Synthesis of lipid RAFT agent (CTA) DMG-PABTC

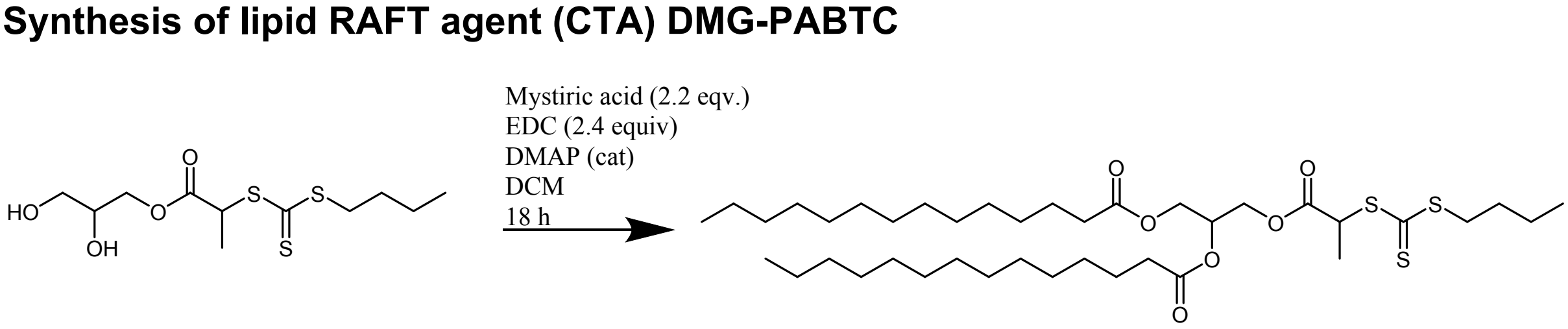


**Schematic S3. Synthesis of DMG-PABTC**

Glycerol-PABTC (1.6 g, 5.31 mmol) myristic acid (tetradecanoic acid; 2.67 g, 11.7 mmol) were dissolved in 100 mL DCM, added to a round bottom flask with a magnetic stirrer and cooled

to 0 °C with an ice bath. DMAP (525 mg, 4.3 mmol, 122.17 g/mol) was dissolved in 5 mL of DCM and added slowly to the reaction mixture with stirring and left for 5 minutes. A solution of EDC-HCl (2.00 g, 12.9 mmol) in DCM (20 mL) was added dropwise with stirring, and left to react overnight, allowing the ice bath left to melt and reach room temperature. A deep red colour was first observed which returned to bright yellow/orange overnight. Reaction progress was monitored to satisfactory completion with thin layer chromatography (TLC) using ethyl acetate (0-40%) in hexane. The resulting reaction mixture was diluted with ~50 mL of chloroform then washed twice with roughly equal volumes of $NH_4Br$ (0.5 M), then washed twice with roughly equal volumes of brine, then dried over $Na_2SO_4$. The product was purified via column chromatography (0-15% Ethyl acetate in hexane, gradient elution). The product fractions were concentrated via rotary evaporation to a viscous yellow oil which solidified upon storage at -20 °C, and was characterised by $^1H$ NMR and $^{13}C$ NMR.

## Polymer synthesis including end group removal

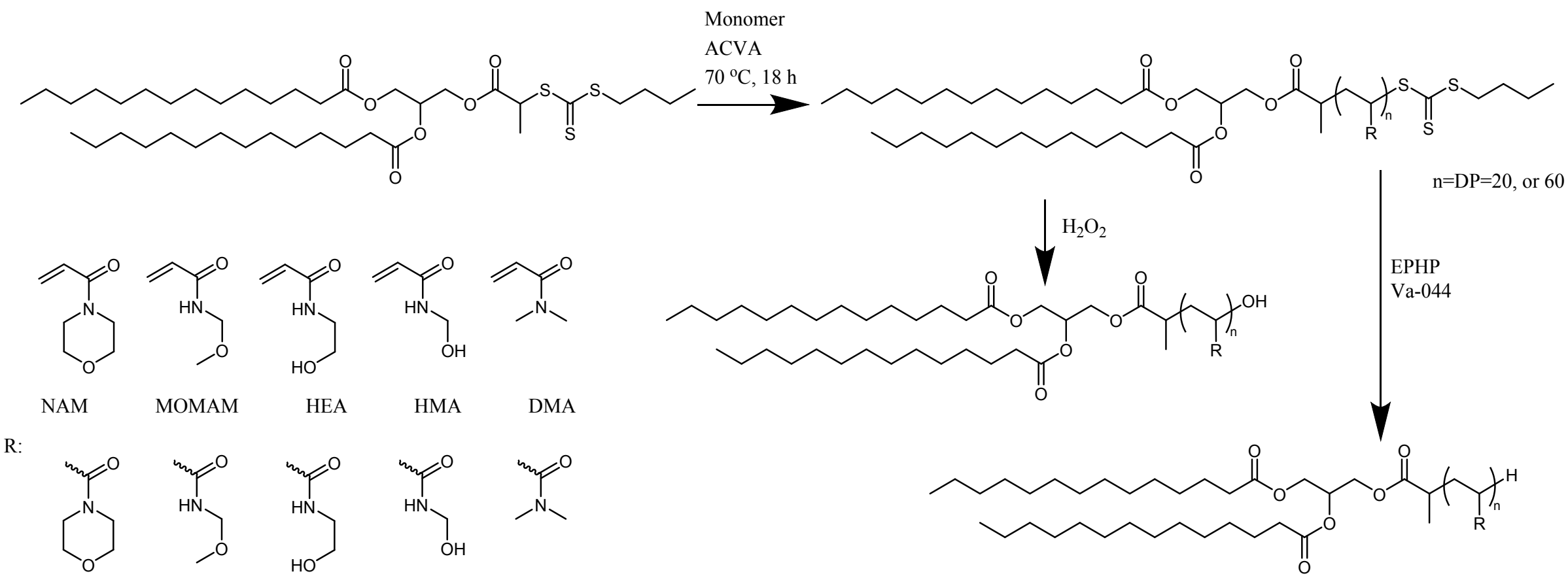


**Schematic S4. Synthesis of poly(acrylamido) lipids via RAFT polymerisation using DMG-CTA, followed by end group removal**

Synthesis of polymers (with $-S_3C_5H_9$ end group) is outlined in materials and methods, with specific conditions and characterisation shown in Table S5. All polymers were prepared using the macro-CTA DMG-PABTC (synthesis described above). The monomers used for each polymer share the abbreviation used for the polymer I.D., e.g. $NAM_{20}$-$S_3C_5H_9$ was synthesised using n-acryloyl morpholine (NAM). Polymers with their end groups replaced were synthesised by taking a portion of the polymer with the $-S_3C_5H_9$ end group and subjecting them to the reaction conditions described below, to yield either -OH or -H end groups. All polymers were characterised by $^1H$ NMR and gel permeation chromatography (GPC) analysis.

*End-group replacement with -OH*

End-group replacement with -OH group was achieved via methods modified from those previously described[9,10]. Briefly, 18 umol of PAM-lipid polymer with the $-S_3C_5H_9$ end group was dissolved to 100 mg/mL in a solvent mix with volumetric ratios of either: $H_2O$:IPA 2:1, $H_2O$:IPA 1:1, or $H_2O$:Dioxane:IPA 1:1:1 depending on polymer solubility. 180 umol of $H_2O_2$ was added from a 30% stock solution in water. The solution was added to a vial equipped with a magnetic stirrer bar stirred at 70 °C for 24 hours without degassing. End group replacement was deemed finished after loss of bright yellow colouration. Where the reaction had not reached satisfactory completion, the same amount of $H_2O_2$ was added to the vial again and left for a further 24 hours up to two more times.

*End-group replacement with -H*

End-group replacement with -H was achieved via methods modified from those previously described[11]. Briefly, polymers with $-S_3C_5H_9$ (~17 umol) and 1-ethylpiperidine hypophosphite (170 umol, 20 mg) were dissolved in 600 uL of dioxane:water 2:1, IPA:water 2:1, or dioxane:IPA:water 1:1:1. Azoinitiator 2,2'-Azobis[2-(2-imidazolin-2-yl)propane]dihydrochloride (VA-044, 1.7 umol, [CTA]/[I]=10, 0.53 mg) was added from a stock solution prepared in $H_2O$. The solution was mixed and added to a reaction vial equipped with a stirrer and rubber septa. The solution was degassed via bubbling nitrogen through for at least 15 minutes then heated to 70 $^oC$ for 2-3 hours. End group removal was deemed finished after loss of bright yellow colouration. Where the reaction had not reached satisfactory completion, the same amount of initiator and EPHP was added to the vial, followed by degassing and heating to 70 $^oC$ for 2-3 hours as before.

## Polymer characterisation

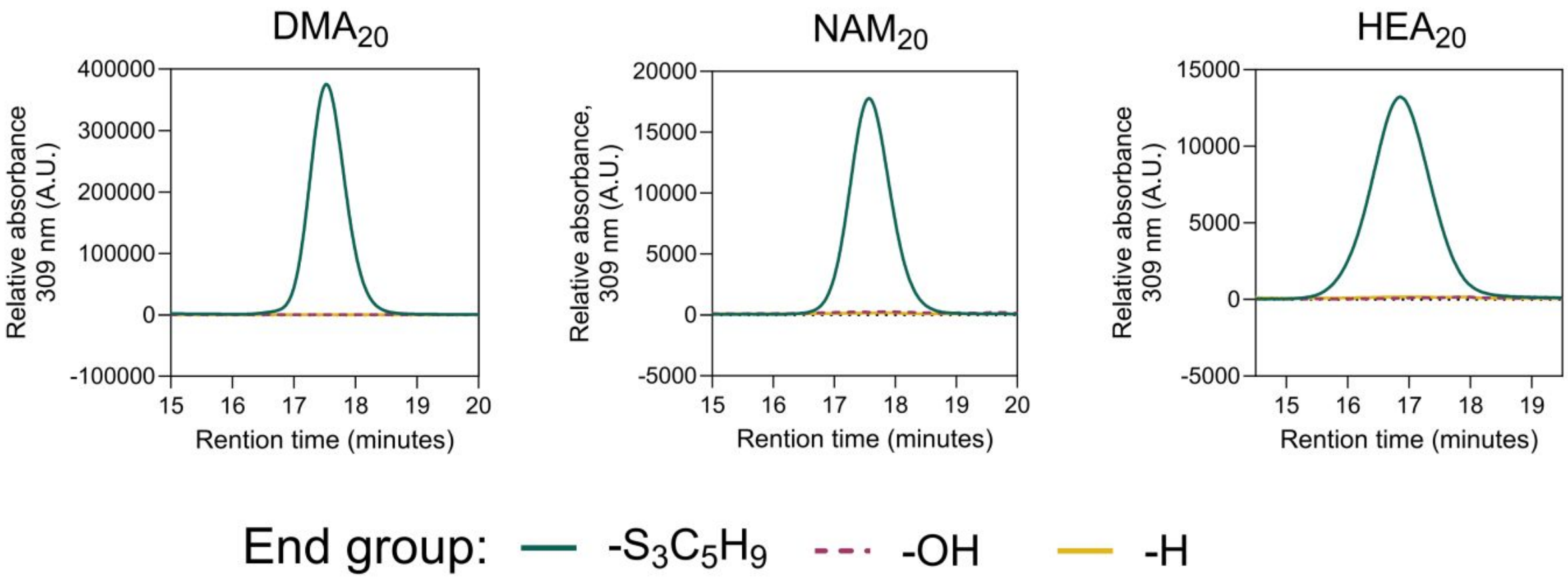


**Figure S9. Confirmation of removal of $-S_3C_5H_9$ end group** Gel permeation chromatography (GPC) coupled to UV/Vis detection of PAM-lipids at characteristic absorbance for trithiocarbonyl group (309 nm) present in $-S_3C_5H_9$ end group.

**Table S5.** Synthesis conditions and characterisation of PAM-lipid polymers prepared with DMG-based macro-CTA before end group removal (-$S_3C_5H_9$) and after end group replacement with -OH, or -H. M = monomer, CTA = chain transfer agent, I = initiator, int= integration under relevant peak in $^1$H NMR. PAMs in green were validated in vivo.

| Polymer I.D. (all prepared with DMG-CTA) | Monomer (mass, mg) | Solvent ratio (Dioxane:Water) | [M] | Target D.P., [M]/[CTA] | [M] at time=0 ($^1$H NMR) | [CTA]/[I] | Conversion, % (from $^1$H NMR) | Final D.P. at t=finished ($^1$H NMR) | $M_{n,\ th,}$ from conversion, g/mol | $M_w$ from GPC, (g/mol) | Dispersity (Mw/Mn) |
|---|---|---|---|---|---|---|---|---|---|---|---|
| $DMA_{20}$-$S_3C_5H_9$ | 250 | 10:0 | 2 | 20 | 19.2 | 20 | 97.0 % | 18.7 | 2586 | 3357 | 1.08 |
| $DMA_{20}$-OH | | | | | | | | | 2438 | 3255 | 1.12 |
| $DMA_{20}$-H | | | | | | | | | 2423 | 2839 | 1.09 |
| $DMA_{60}$-$S_3C_5H_9$ | 350 | 10:0 | 2 | 60 | 66.0 | 20 | 95.5 % | 63.0 | 6978 | 8652 | 1.05 |
| $DMA_{60}$-OH | | | | | | | | | 6830 | 9520 | 1.10 |
| $DMA_{60}$-H | | | | | | | | | 6814 | 8712 | 1.07 |
| $NAM_{20}$-$S_3C_5H_9$ | 250 | 10:0 | 2 | 20 | 25.34 | 20 | 98.7 % | 25 | 4262 | 3363 | 1.07 |
| $NAM_{20}$-OH | | | | | | | | | 4114 | 3732 | 1.09 |
| $NAM_{20}$-H | | | | | | | | | 4098 | 3517 | 1.06 |
| $NAM_{60}$-$S_3C_5H_9$ | 350 | 10:0 | 2 | 60 | 55.7 | 20 | 99.0% | 55.1 | 8497 | 7249 | 1.20 |
| $NAM_{60}$-OH | | | | | | | | | 8349 | 7765 | 1.24 |
| $NAM_{60}$-H | | | | | | | | | 8333 | 8045 | 1.11 |
| $MOMAM_{20}$-$S_3C_5H_9$ | 250 | 10:0 | 1.5 | 20 | 19.3 | 20 (2 additions) | 98.5 % | 19 | 2920 | 4825 | 1.07 |
| $MOMAM_{20}$-OH | | | | | | | | | 2772 | 5691 | 1.13 |

| | | | | | | | | | | | |
|---|---|---|---|---|---|---|---|---|---|---|---|
| MOMAM$_{20}$-H | | | | | | | | | 2756 | 5309 | 1.17 |
| MOMAM$_{60}$-$S_3C_5H_9$ | 350 | 10:0 | 1.5 | 60 | - | 20 (3 additions) | - | - | - | 22253 | 1.34 |
| MOMAM$_{60}$-OH | | | | | | | | | - | 29119 | 1.31 |
| MOMAM$_{60}$-H | | | | | | | | | - | 24612 | 1.43 |
| HEA$_{20}$-$S_3C_5H_9$ | 250 | 8:2 | 2 | 20 | 17.5 | 20 | 95.4 % | 16.7 | 2656 | 6551 | 1.18 |
| HEA$_{20}$-OH | | | | | | | | | 2508 | - | - |
| HEA$_{20}$-H | | | | | | | | | 2492 | - | - |
| HEA$_{60}$-$S_3C_5H_9$ | 350 | 8:2 | 2 | 60 | 65 | 20 | 98.5% | 64 | 8101 | 10363 | 1.32 |
| HEA$_{60}$-OH | | | | | | | | | 7953 | 6817 | 1.12 |
| HEA$_{60}$-H | | | | | | | | | 7937 | 23492 | 1.30 |
| HMA$_{20}$-$S_3C_5H_9$ | 250 | 4:6 | 1 | 20 | 20.5 | 20 | 98.0 % | 20.0 | 2755 | 4964 | 1.16 |
| HMA$_{20}$-OH | | | | | | | | | 2607 | 6529 | 1.20 |
| HMA$_{20}$-H | | | | | | | | | 2591 | | |
| HMA$_{60}$-$S_3C_5H_9$ | 350 | 4:6 | 1 | 60 | 52.1 | 20 | 96.2 % | 50.0 | 5788 | 13856 | 1.07 |
| HMA$_{60}$-OH | | | | | | | | | 5640 | - | - |
| HMA$_{60}$-H | | | | | | | | | 5624 | - | - |

# Flow cytometry gating strategies

## BMDCs

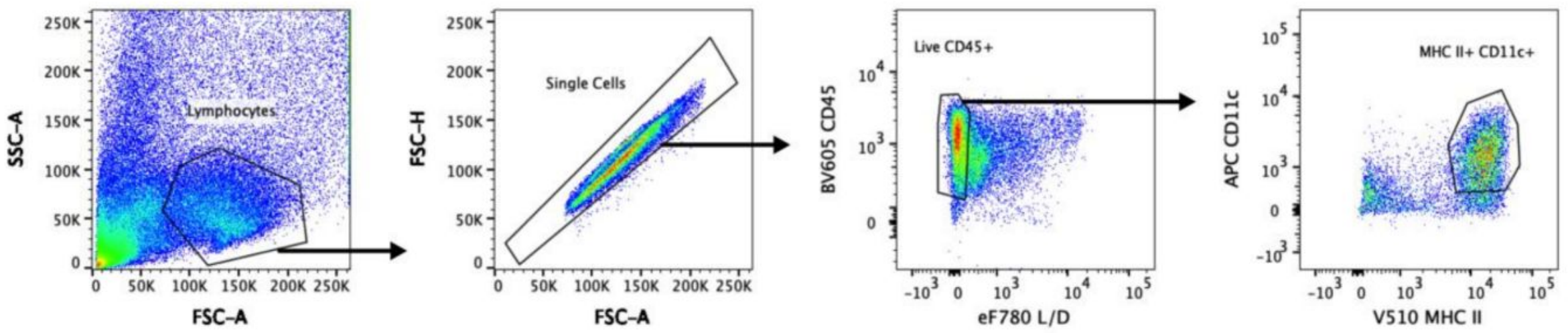


**Figure S10. Flow cytometry gating strategy for BMDCs.**

Representative gating strategy for live BMDCs (cells, single cells, live CD45+, Lin – (CD19 NK1.1 B220 Ly6c Ly6g), MHC II+ CD11c+).

## THP-1s

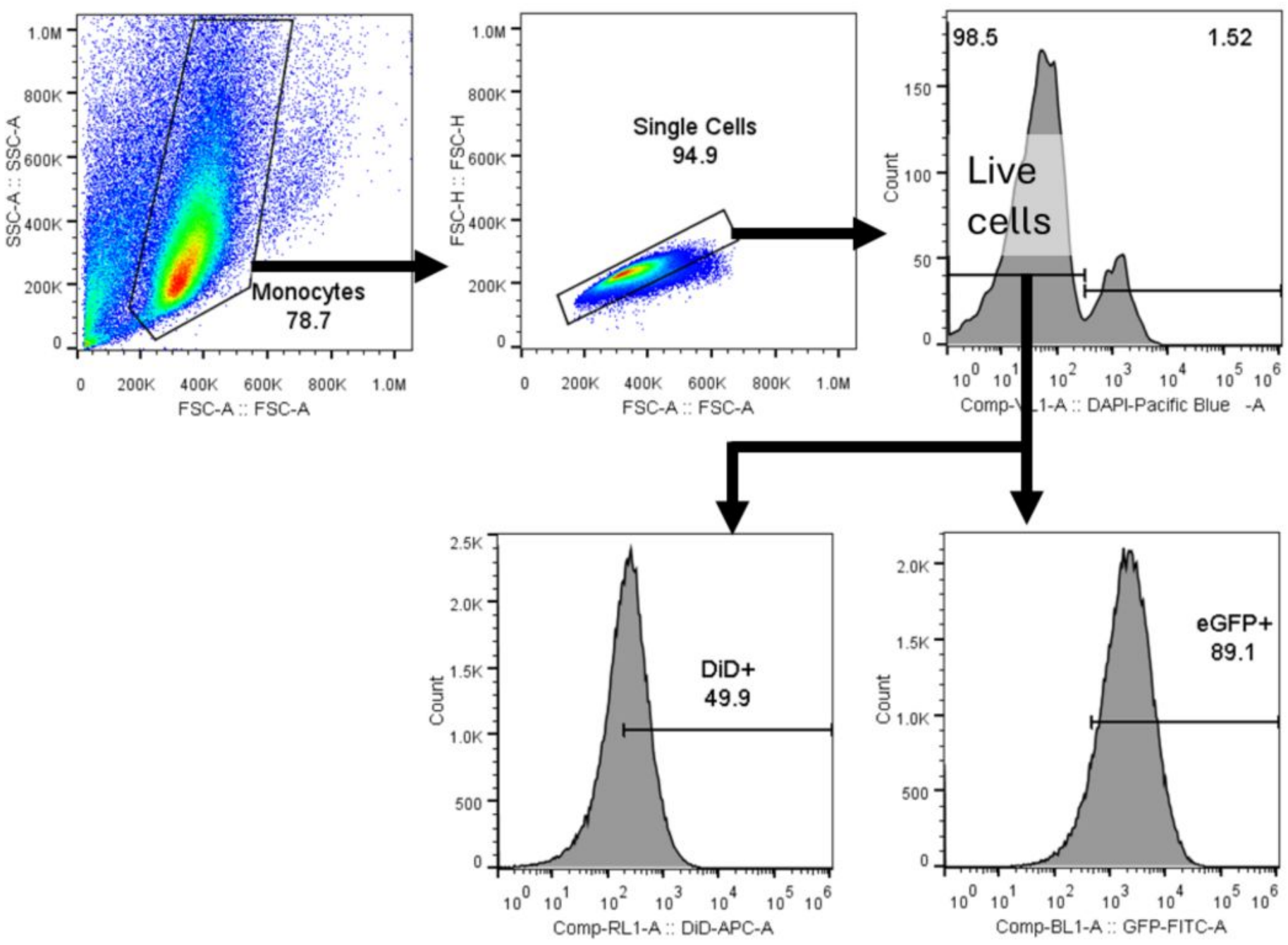


**Figure S11. Flow cytometry gating strategy for THP-1s** Monocytes, single cells, then live cells (DAPI-negative) before analysis of DiD and eGFP fluorescence.

### Cells from liver, spleen and lymph nodes after ID administration of PAM-LNPs and PEG-LNPs In vivo

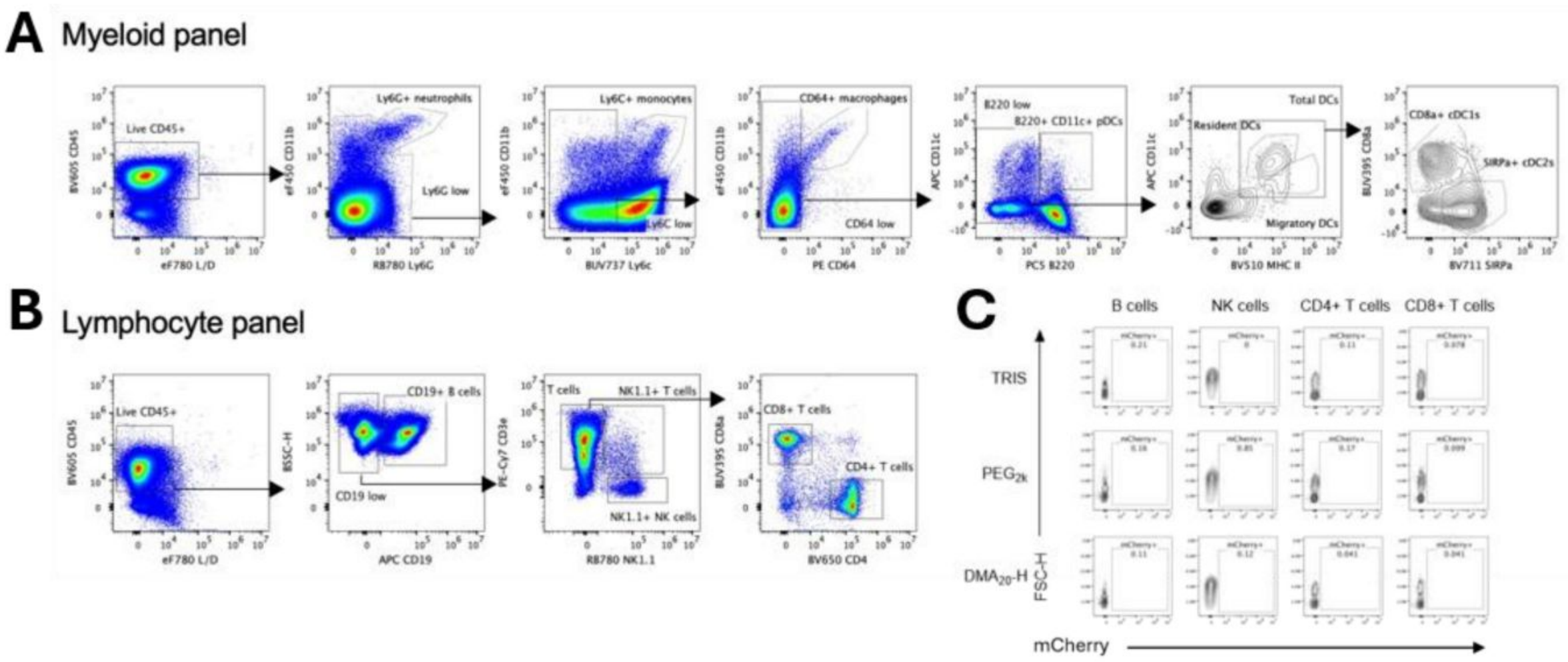


**Figure S12. Flow cytometry gating strategy for cells from liver, spleen and lymph nodes after ID administration of PAM-LNPs and PEG-LNPs**

**A** Representative flow cytometry plots for gating using the myeloid panel to identify neutrophils (singlets, live, CD45+, CD11b+, Ly6G+), monocytes (singlets, live, CD45+, CD11b+, Ly6C+), macrophages (singlets, live, CD45+, CD11b+, CD64+), plasmacytoid dendritic cells (singlets, live, CD45+ B220+ CD11c+), total conventional dendritic cells (cDCs) (singlets, live, CD45+, MHC II+, CD11c+), resident cDCs (singlets, live, CD45+, MHC II+, CD11c$^{hi}$), migratory cDCs (singlets, live, CD45+, MHC II$^{hi}$, CD11c+), cDC1s (singlets, live, CD45+, MHC II+ CD11c+, CD8a+) and cDC2s (singlets, live, CD45+, MHC II+ CD11c+, SIRPα+). **B** Representative flow cytometry plots for gating using the lymphocyte panel to identify B cells (singlets, live, CD45+, CD19+), NK cells (singlets, live, CD45+, NK1.1+), NK T cells (singlets, live, CD45+, NK1.1+, CD3e+), CD4+ T cells (singlets, live, CD45+, CD3e+ CD4+) and CD8+ T cells (singlets, live, CD45+, CD3e+ CD8+). **C** Representative flow cytometry plots of mCherry fluoresence in lymphocytes after administration of Tris (negative control) or mCherry mRNA-LNPs prepared with $PEG_{2k}$ or $DMA_{20}$-H

**Table S6.** Antibodies used to stain cells from ID administration of mCherry loaded LNPs ahead of spectral flow cytometry

| Antibody | Supplier | Catalogue number | Dilution |
|---|---|---|---|
| Anti-mouse CD3e PE-Cy7 | Biolegend | 100320 | 1:200 |
| Anti-mouse CD4 BV650 | Biolegend | 100555 | 1:200 |
| Anti-mouse CD8 BUV395 | BD Biosciences | 563786 | 1:200 |
| Anti-mouse CD11b eF450 | Thermo Fisher Scientific | 48-0112-82 | 1:400 |
| Anti-mouse CD11c APC | BD Biosciences | 550261 | 1:100 |
| Anti-mouse CD19 APC | eBiosciences | 17-0193-82 | 1:400 |

| | | | |
|---|---|---|---|
| Anti-mouse CD19 APC Cy7 | BD Biosciences | 557655 | 1:200 |
| Anti-mouse CD45 BV605 | Biolegend | 103155 | 1:1000 |
| Anti-mouse CD64 PE | Biolegend | 139304 | 1:200 |
| Anti-mouse B220 PC5 | BD Biosciences | 553091 | 1:400 |
| Anti-mouse Ly6C BUV737 | BD Biosciences | 755201 | 1:300 |
| Anti-mouse Ly6C APC Cy7 | Biolegend | 128025 | 1:300 |
| Anti-mouse Ly6G RB780 | BD Biosciences | 569153 | 1:200 |
| Anti-mouse Ly6G APC Cy7 | Biolegend | 127624 | 1:200 |
| Anti-mouse MHC II BV510 | Biolegend | 107636 | 1:800 |
| Anti-mouse NK1.1 RB780 | BD Biosciences | 569232 | 1:200 |
| Anti-mouse NK1.1 APC Cy7 | Biolegend | 108724 | 1:200 |
| Anti-mouse SIRPα BV711 | BD Biosciences | 740766 | 1:100 |

# NMR spectra

## DMG-PABTC lipid RAFT agent CTA

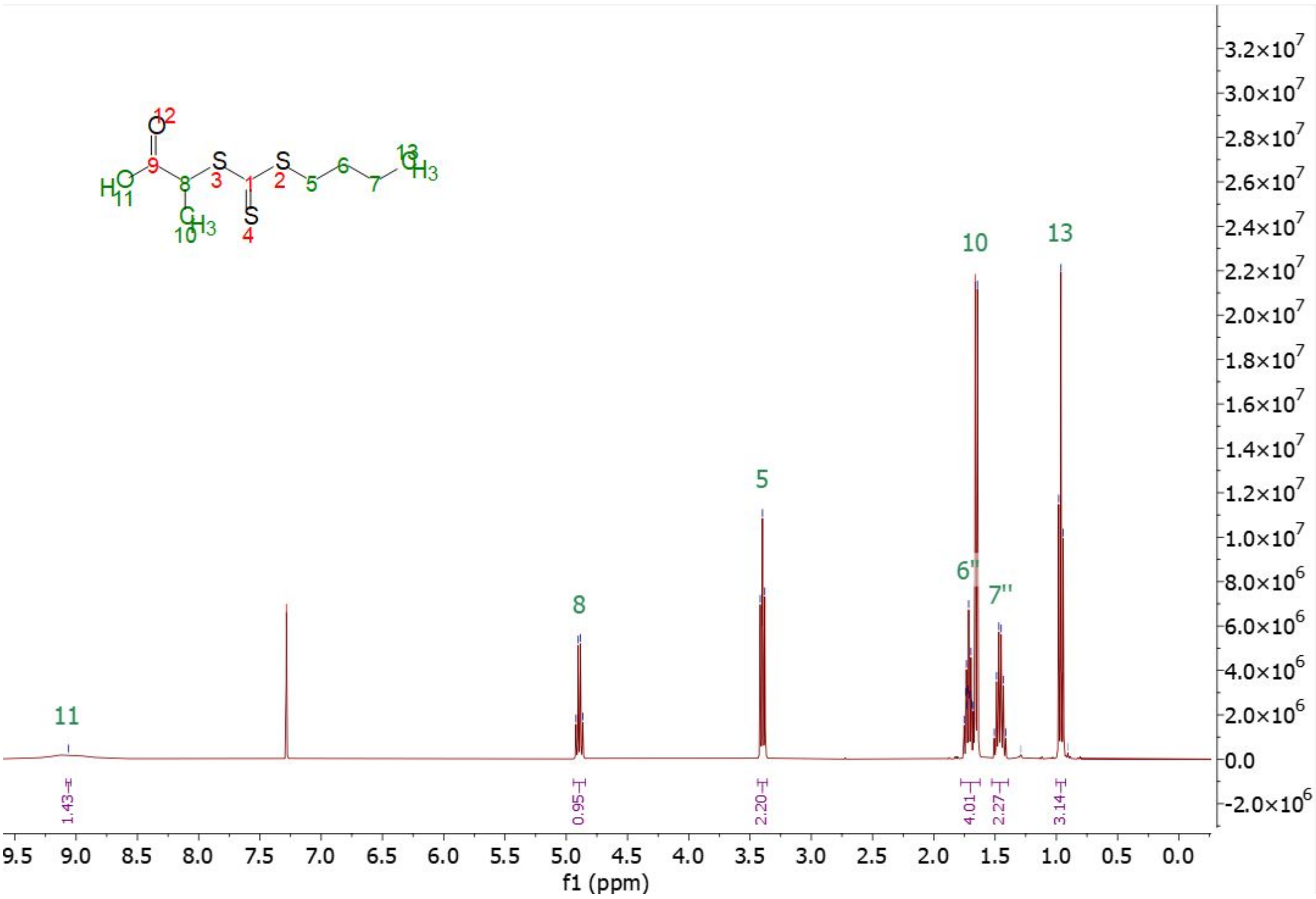


**Figure S13.** $^1$H NMR ($CDCl_3$) of PABTC

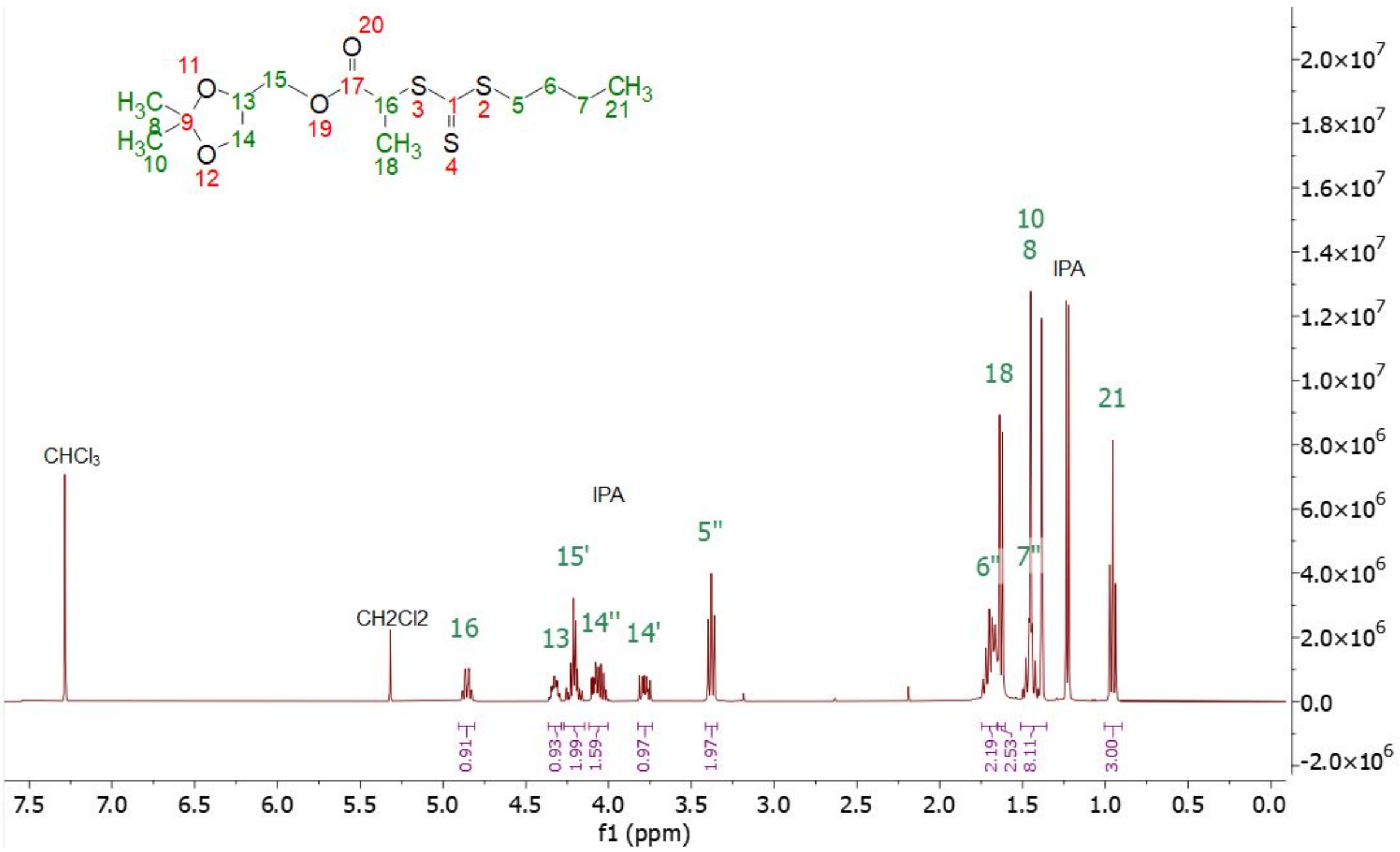


**Figure S14.** $^1$H NMR ($CDCl_3$) of Solketal-PABTC

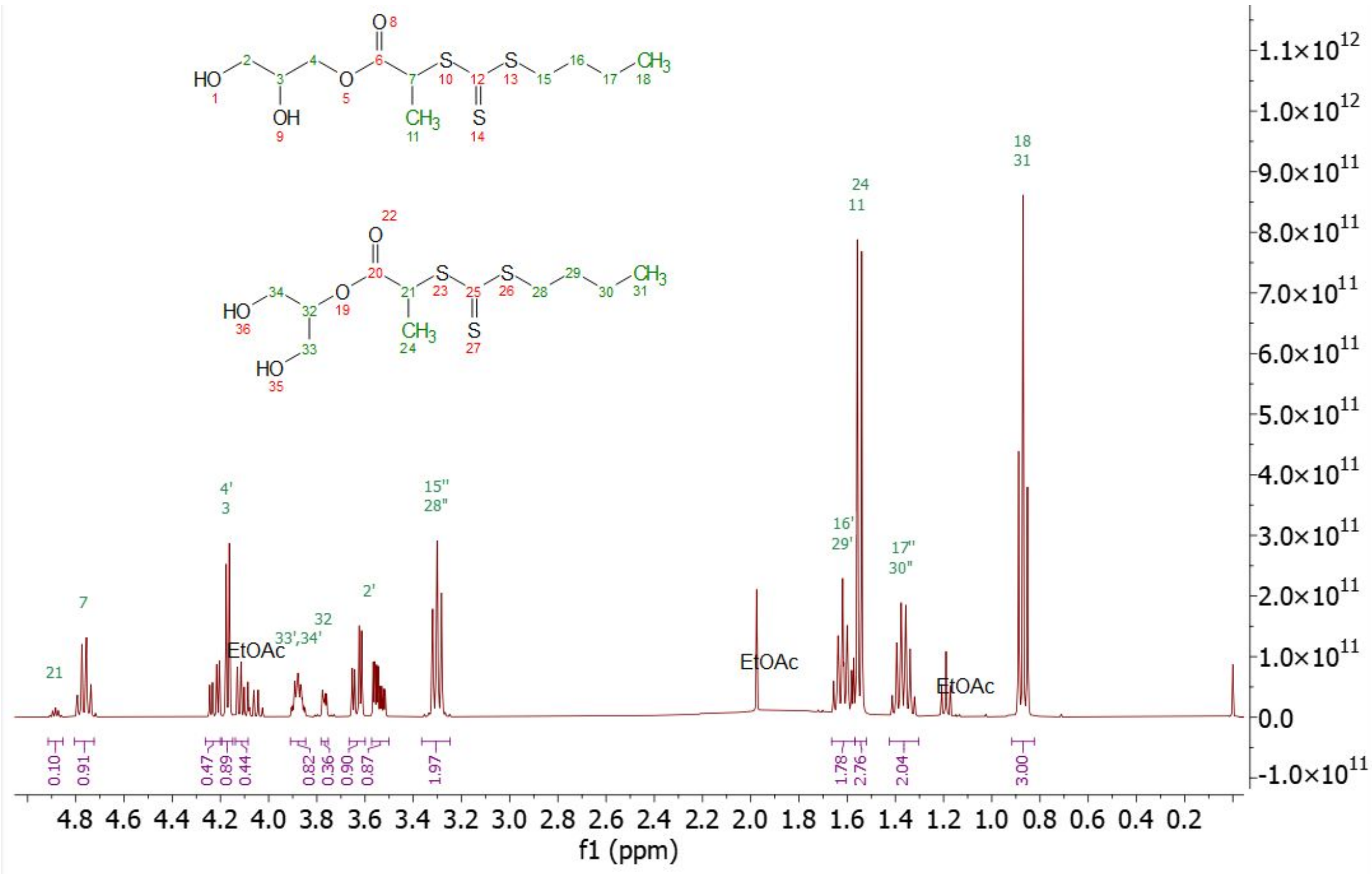

**Figure S15.** $^1H$ NMR ($CDCl_3$) of Glycerol-PABTC

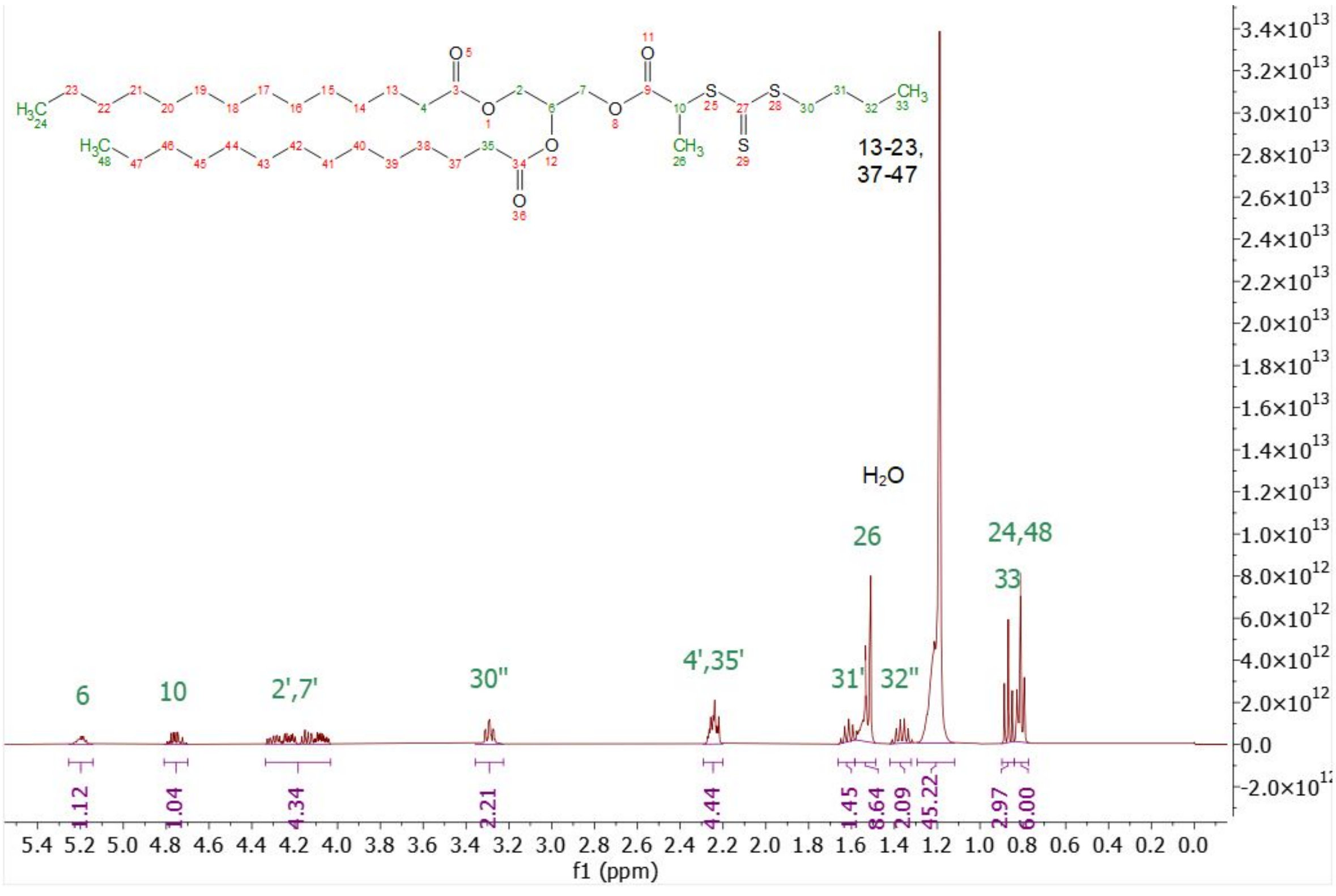

**Figure S16.** $^1H$ NMR ($CDCl_3$) of DMG-PABTC

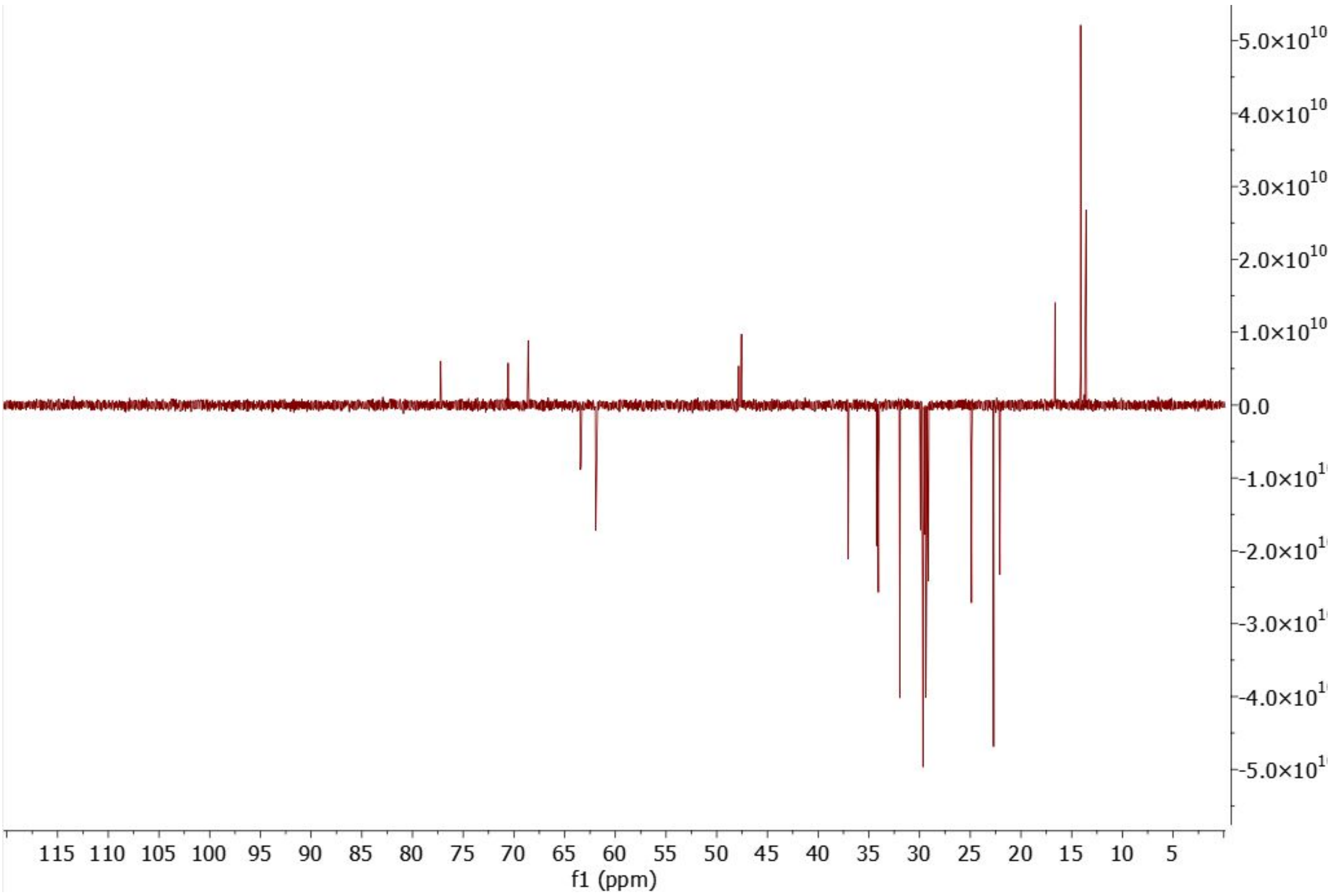


**Figure S17.** $^{13}C$ NMR ($CDCl_3$) of DMG-PABTC

## PAM polymer synthesis

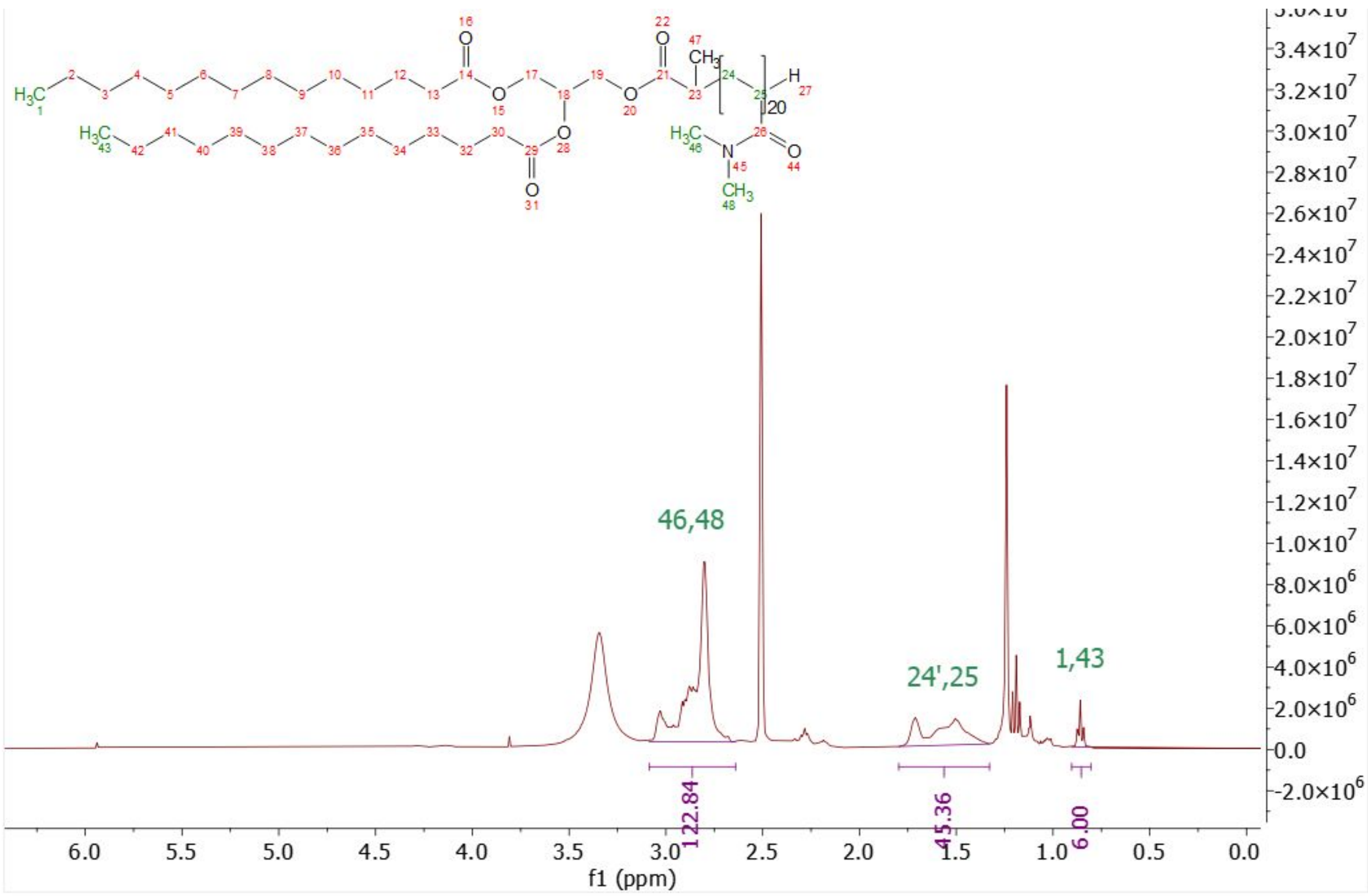


**Figure S18.** $^{1}H$ NMR (d6-DMSO, 400 MHz) of $DMA_{20}$-H

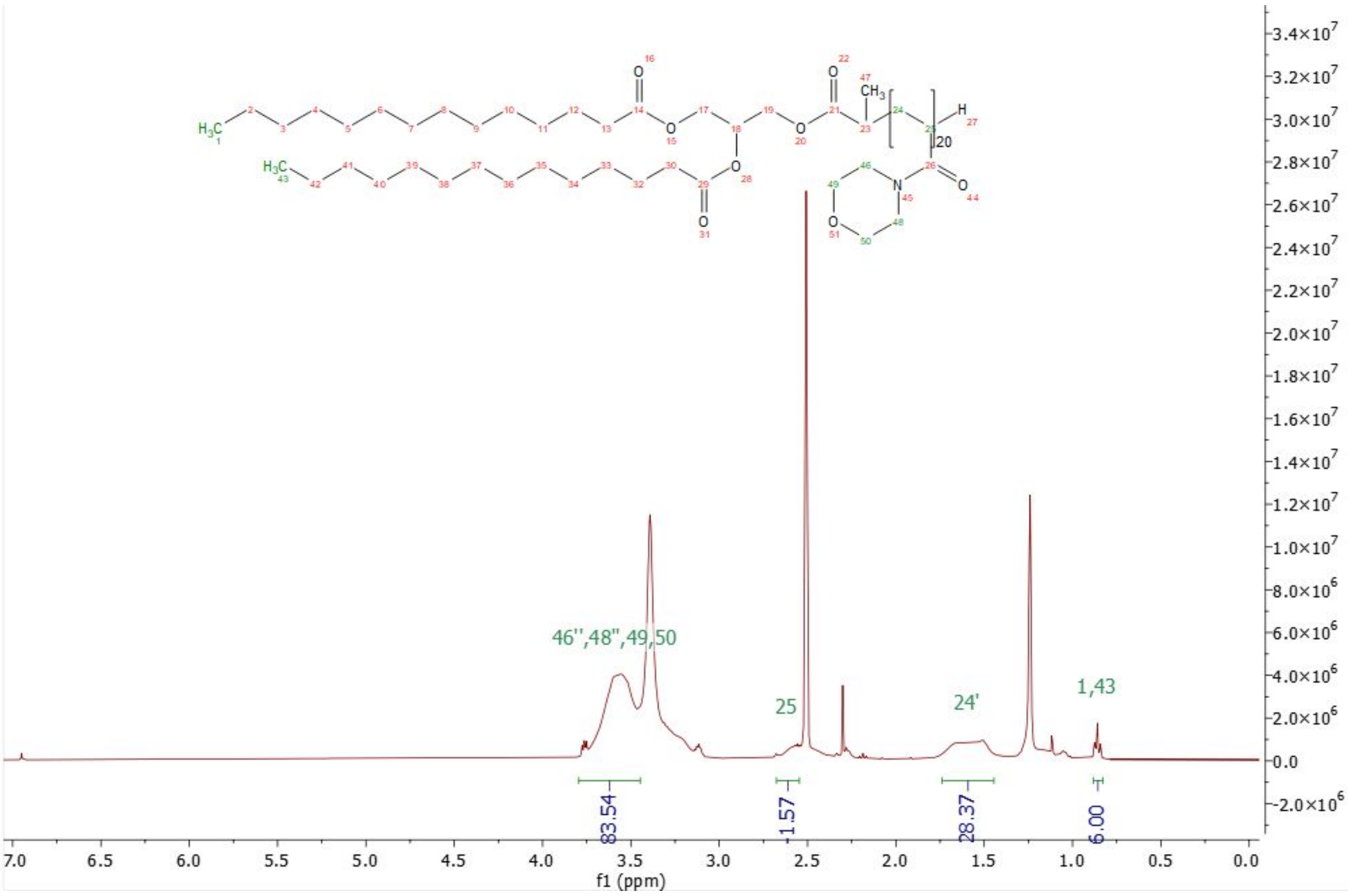


**Figure S19.** $^{1}$H NMR (d6-DMSO, 400 MHz) of $NAM_{20}$-H

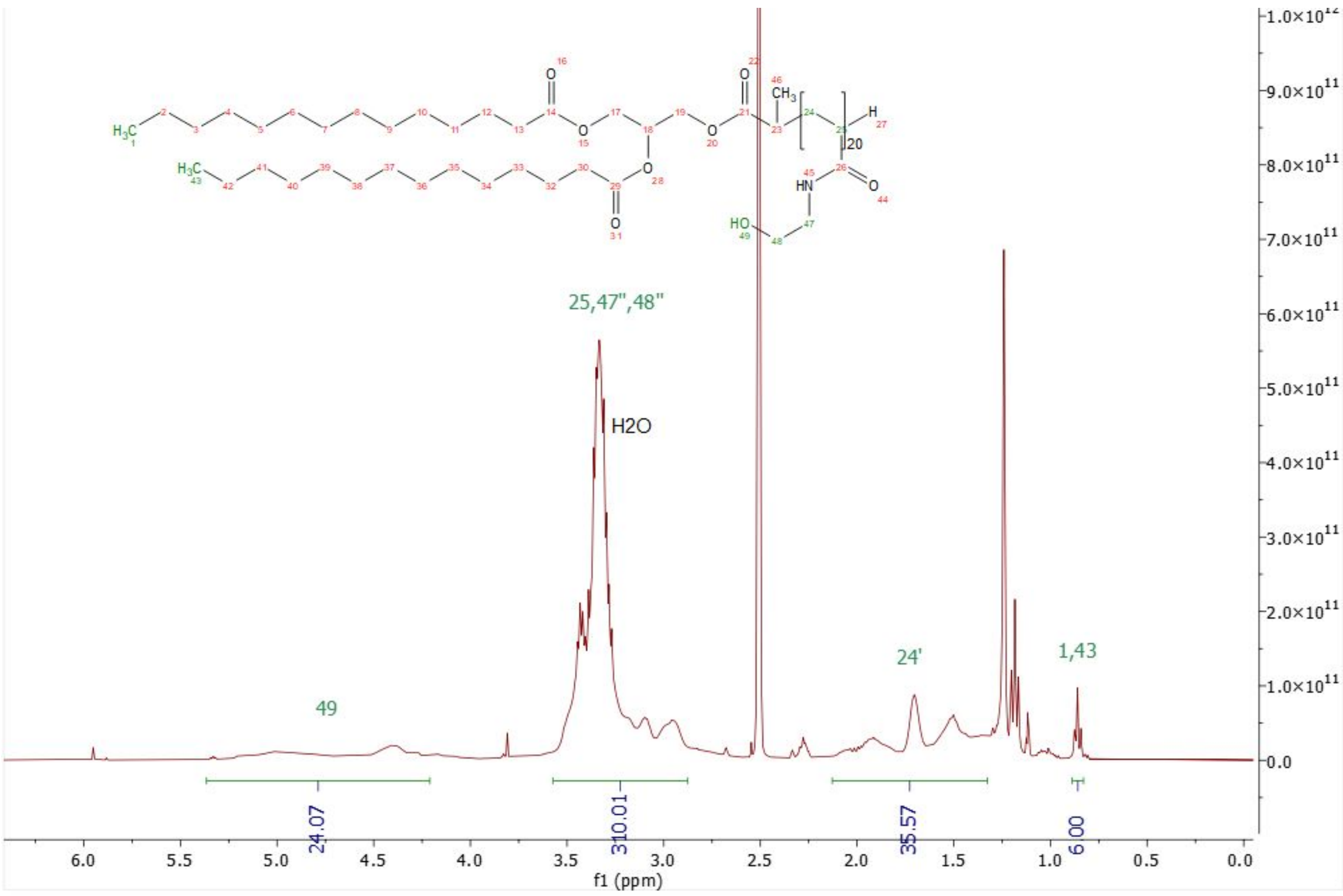


**Figure S20.** $^{1}$H NMR (d6-DMSO, 400 MHz) of $HEA_{20}$-H

*Remainder of 30 PAM-lipid library*

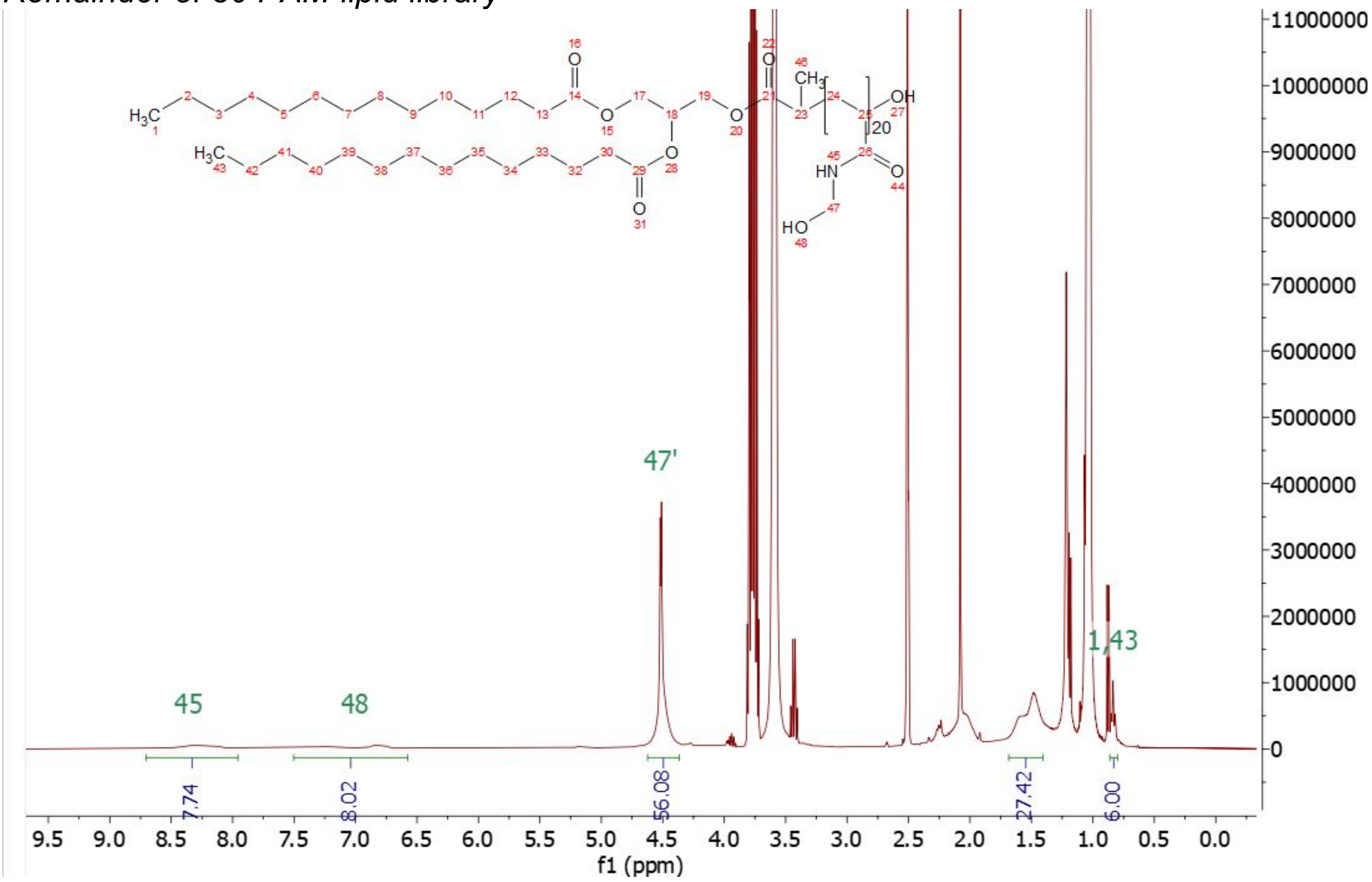


**Figure S21.** $^{1}$H NMR (d6-DMSO, 400 MHz) $HMA_{20}$-OH

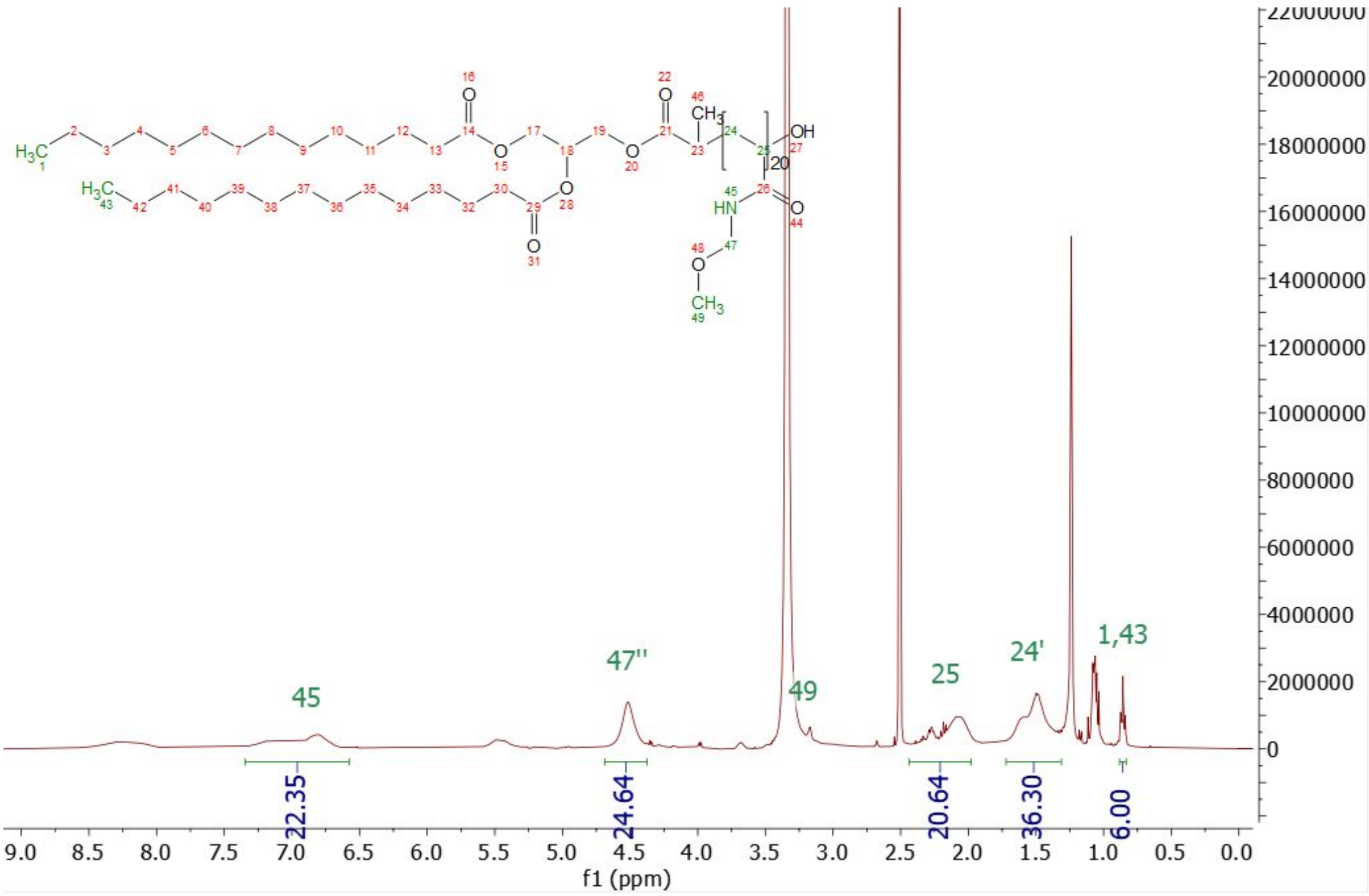


**Figure S22.** $^{1}$H NMR (d6-DMSO, 400 MHz) $MOMAM_{20}$-OH

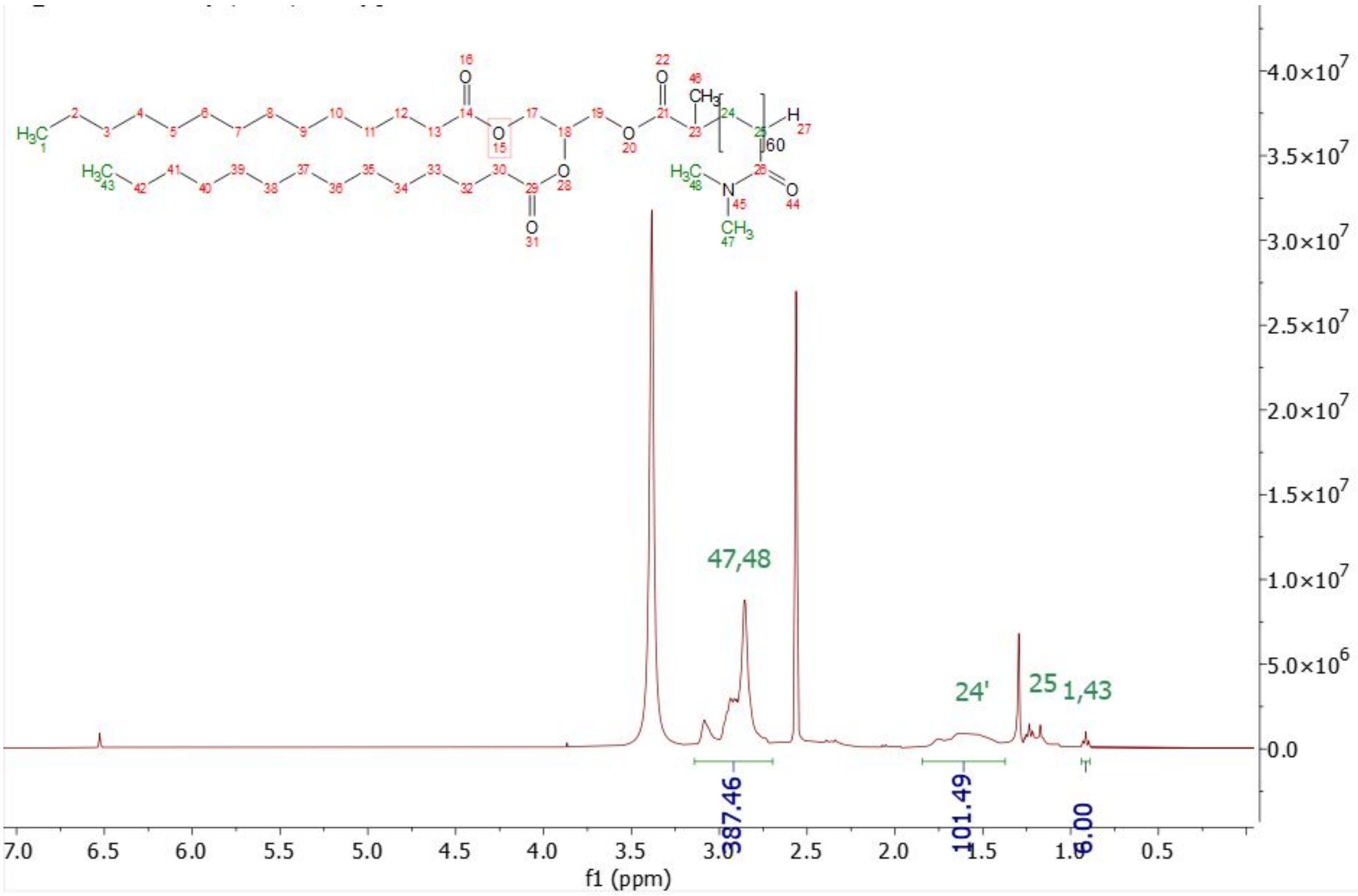


**Figure S23.** $^1$H NMR (d6-DMSO, 400 MHz) $DMA_{60}$-H

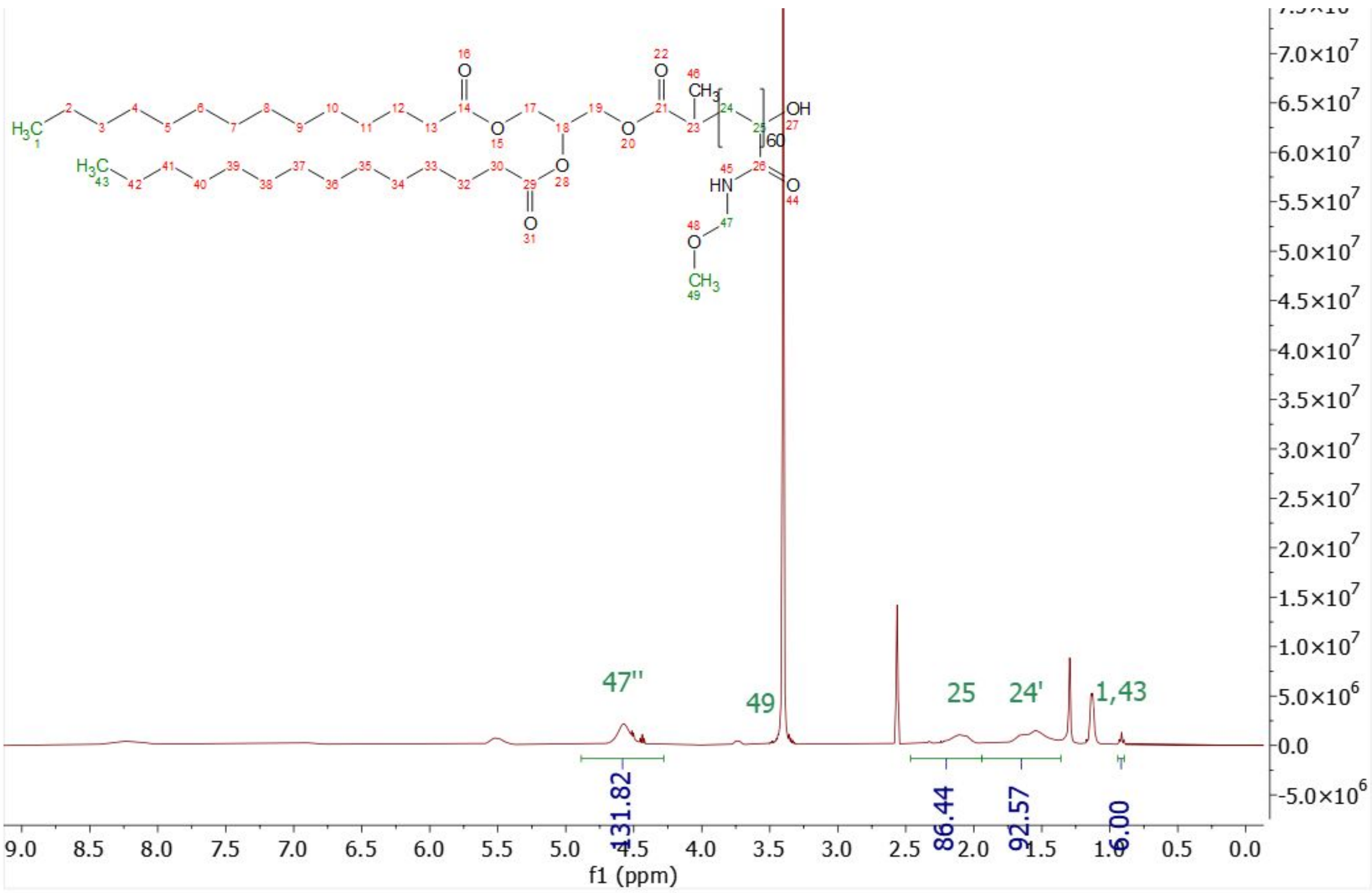


**Figure S24.** $^1$H NMR (d6-DMSO, 400 MHz) $MOMAM_{60}$-OH

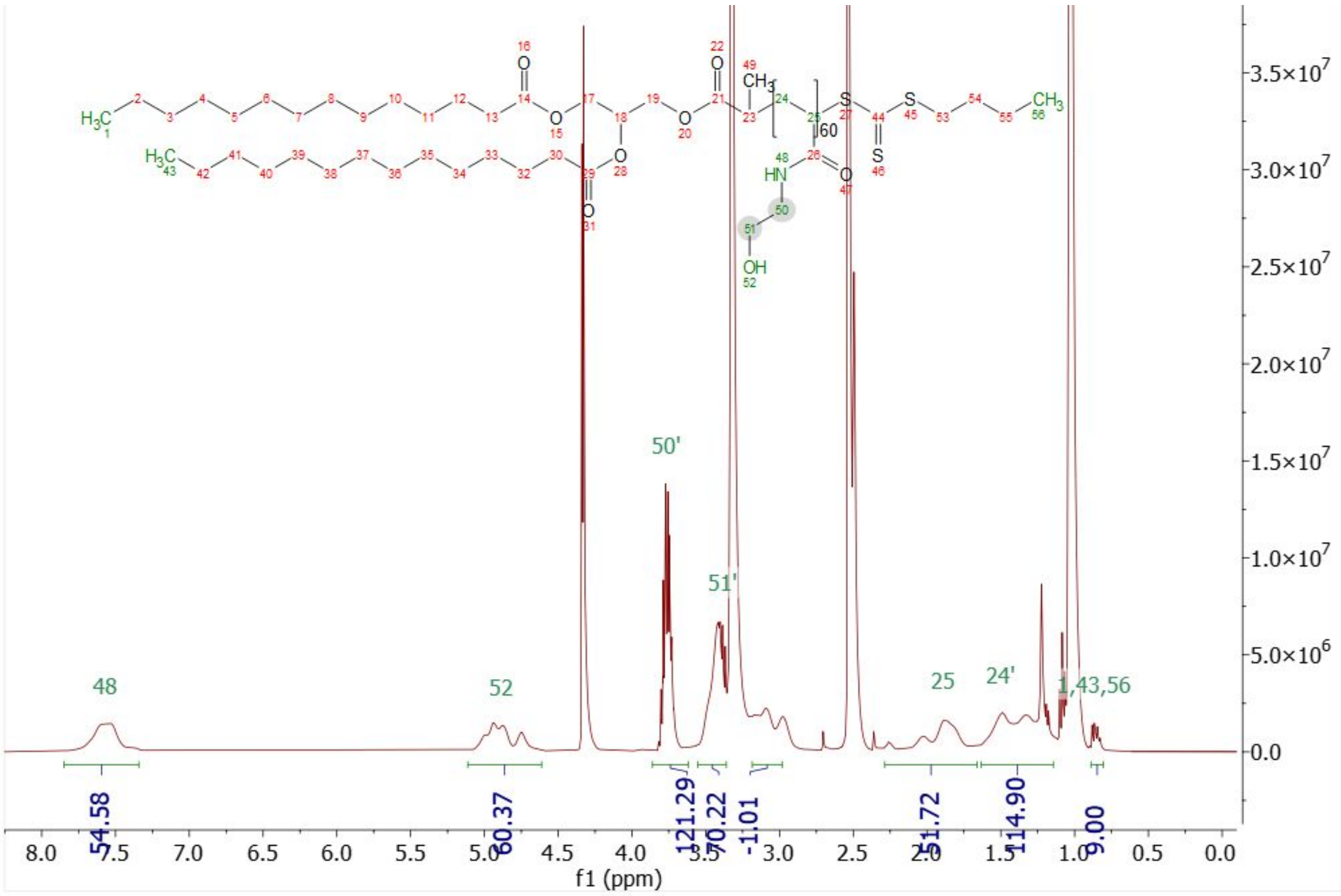


**Figure S25.** $^{1}$H NMR (d6-DMSO, 400 MHz) $HEA_{60}$-S

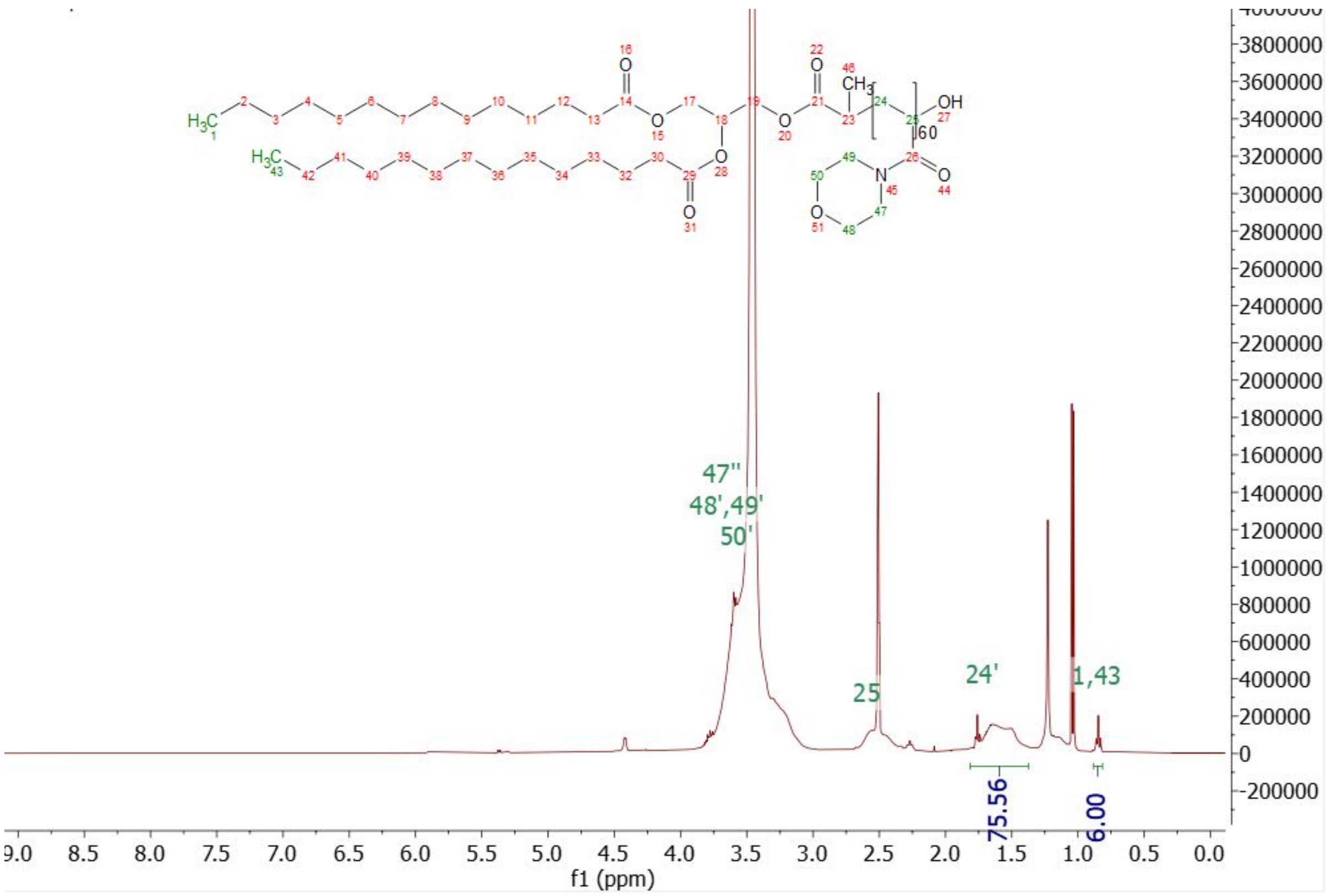


**Figure S26.** $^{1}$H NMR (d6-DMSO, 400 MHz) $NAM_{60}$-OH

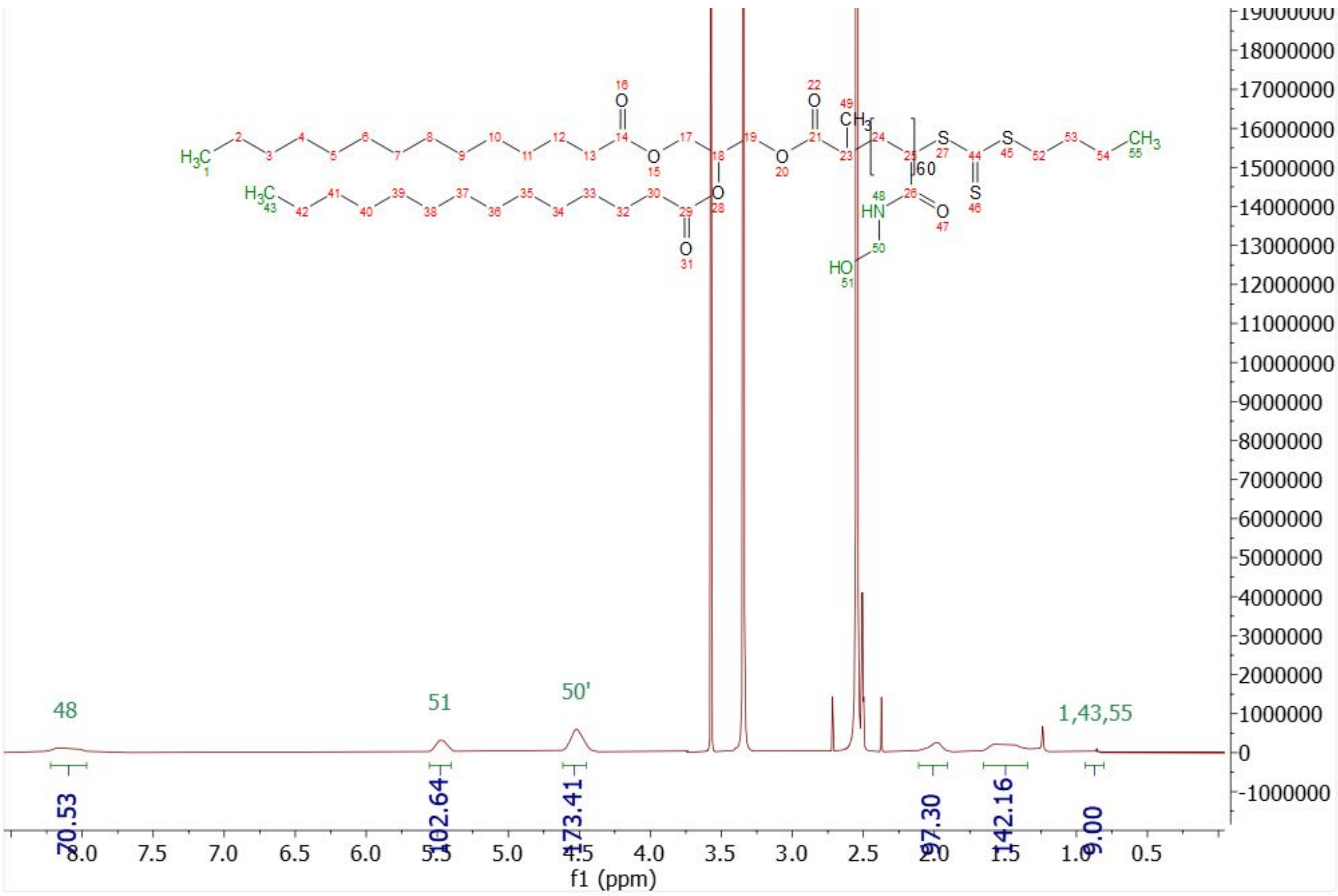

**Figure S27.** $^{1}H$ NMR (d6-DMSO, 400 MHz) $HMA_{60}$-S

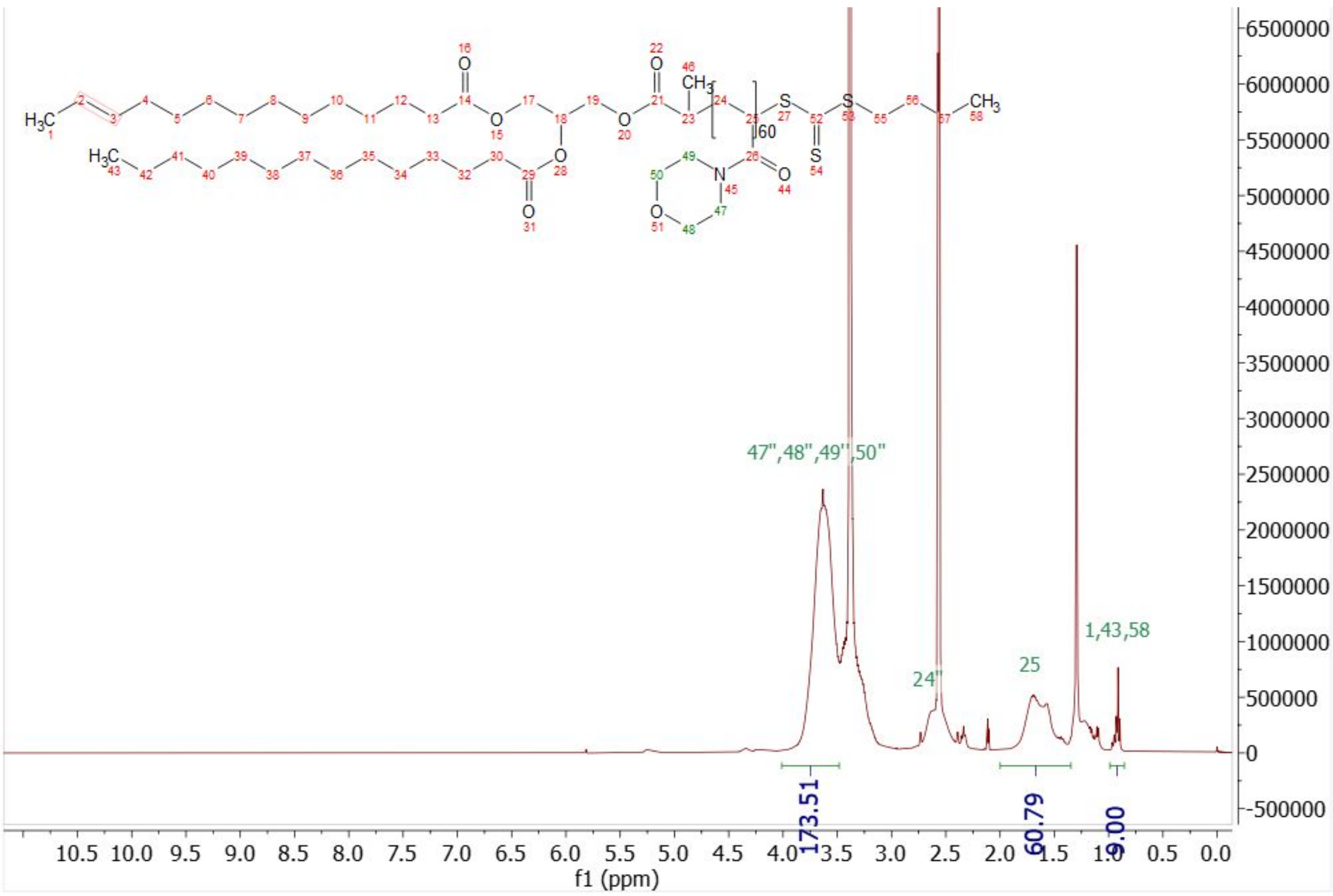

**Figure S28.** $^{1}H$ NMR (d6-DMSO, 400 MHz) $NAM_{60}$-S

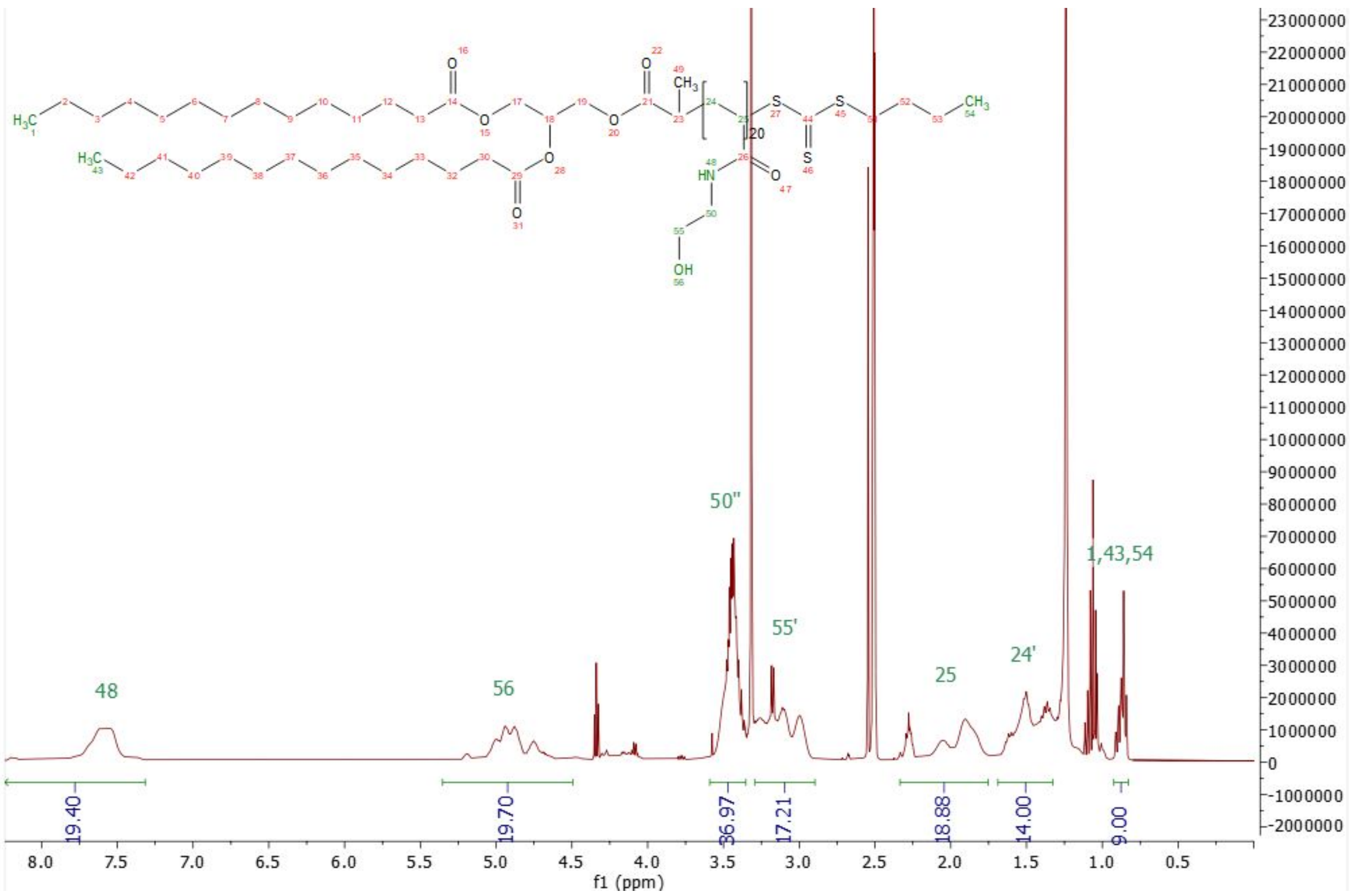


**Figure S29.** $^1$H NMR (d6-DMSO, 400 MHz) $HEA_{20}$-S

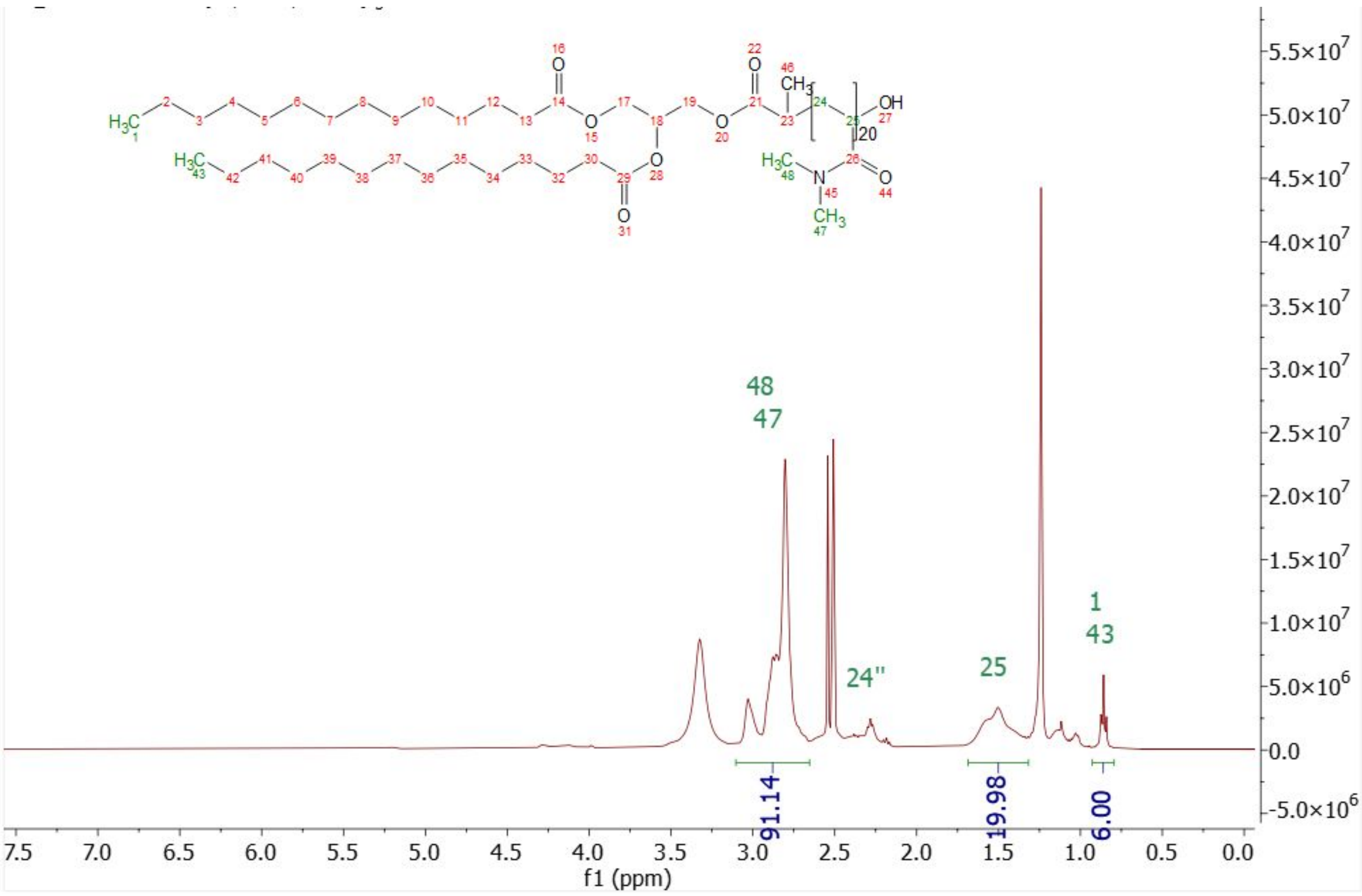


**Figure S30.** $^1$H NMR (d6-DMSO, 400 MHz) $DMA_{20}$-OH

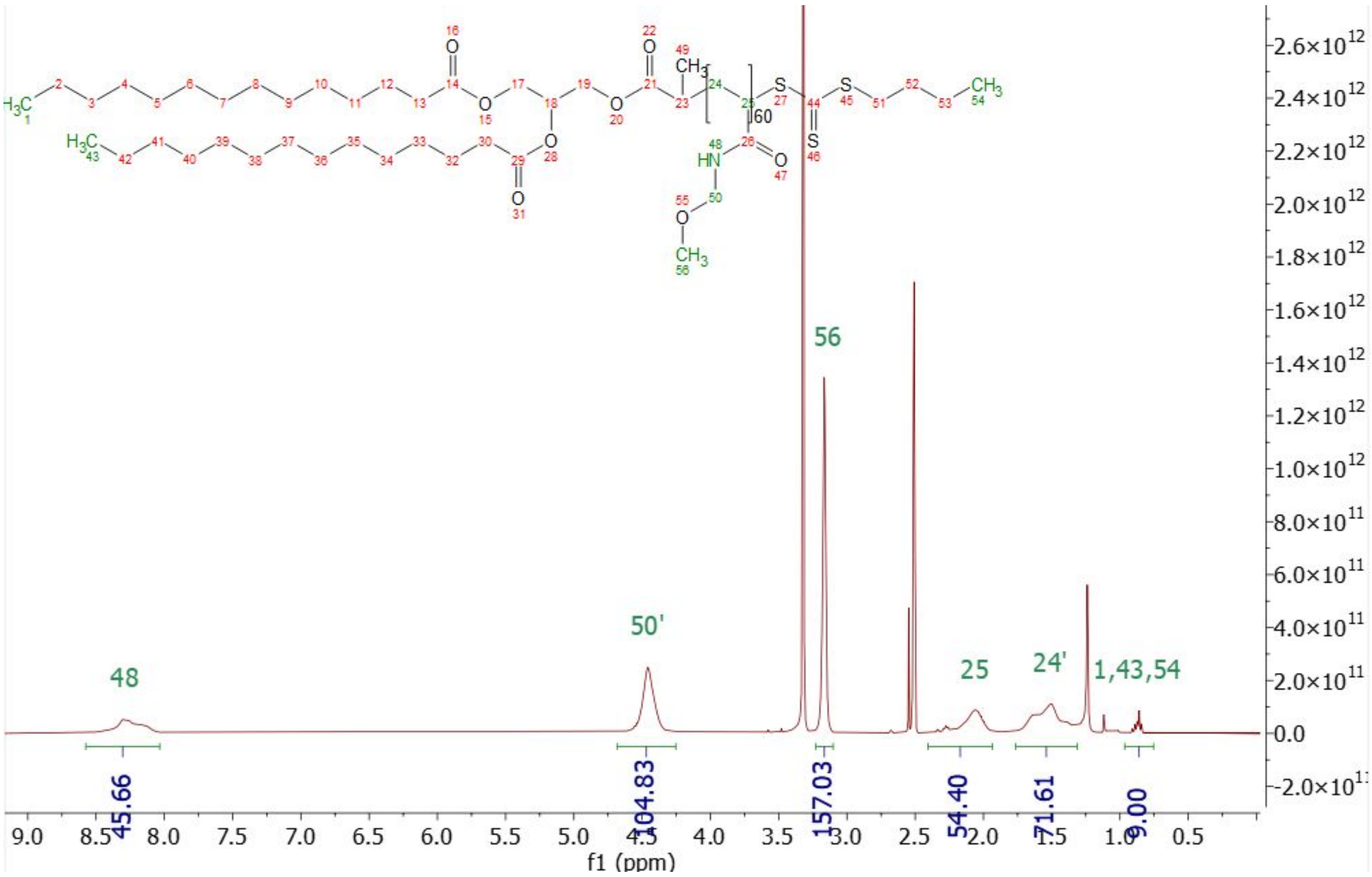


**Figure S31.** $^1$H NMR (d6-DMSO, 400 MHz) $MOMAM_{60}$-S

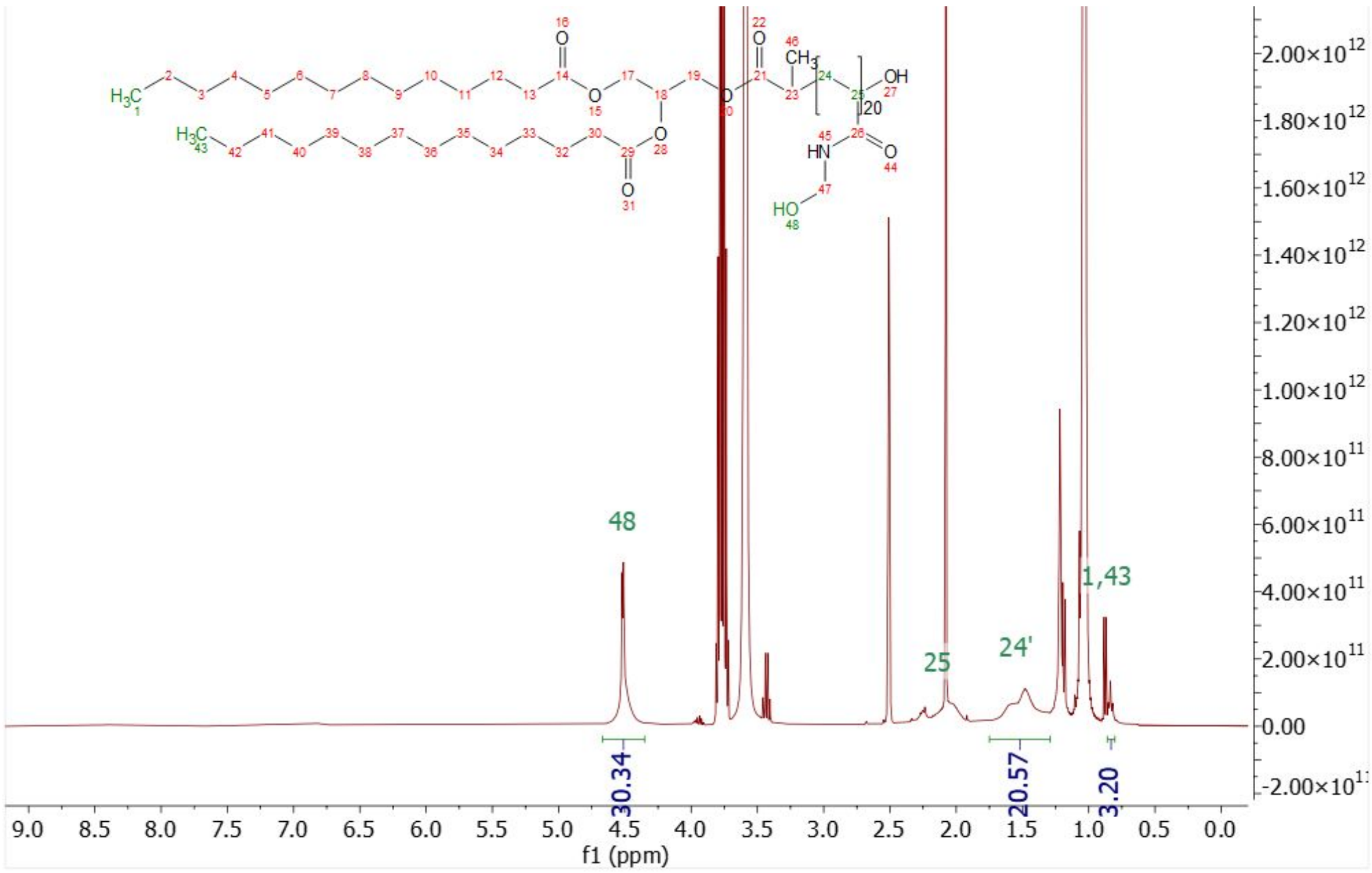


**Figure S32.** $^1$H NMR (d6-DMSO, 400 MHz) $HMA_{60}$-OH

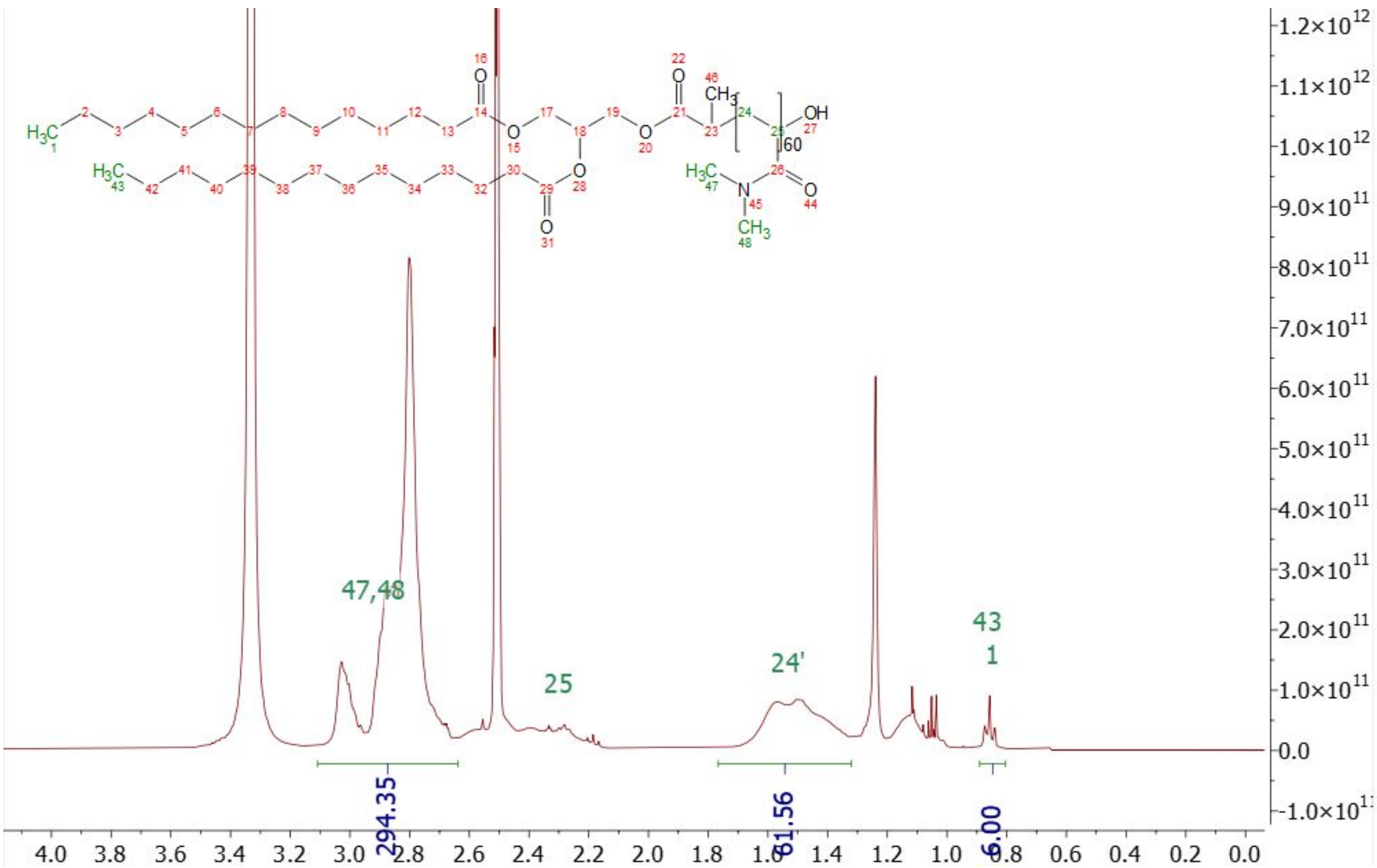


**Figure S33.** $^{1}$H NMR (d6-DMSO, 400 MHz) $DMA_{60}$-OH

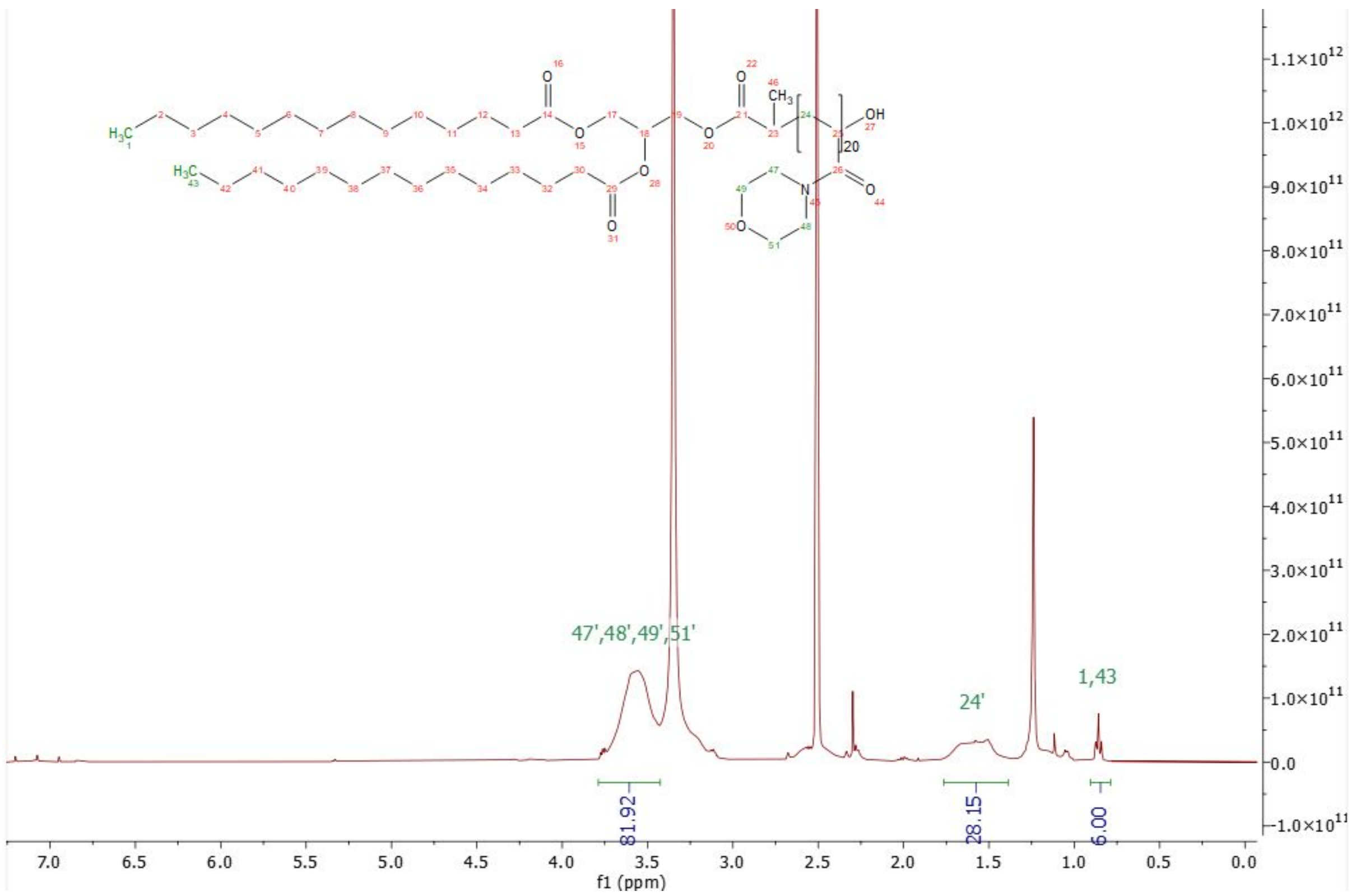


**Figure S34.** $^{1}$H NMR (d6-DMSO, 400 MHz) $NAM_{20}$-OH

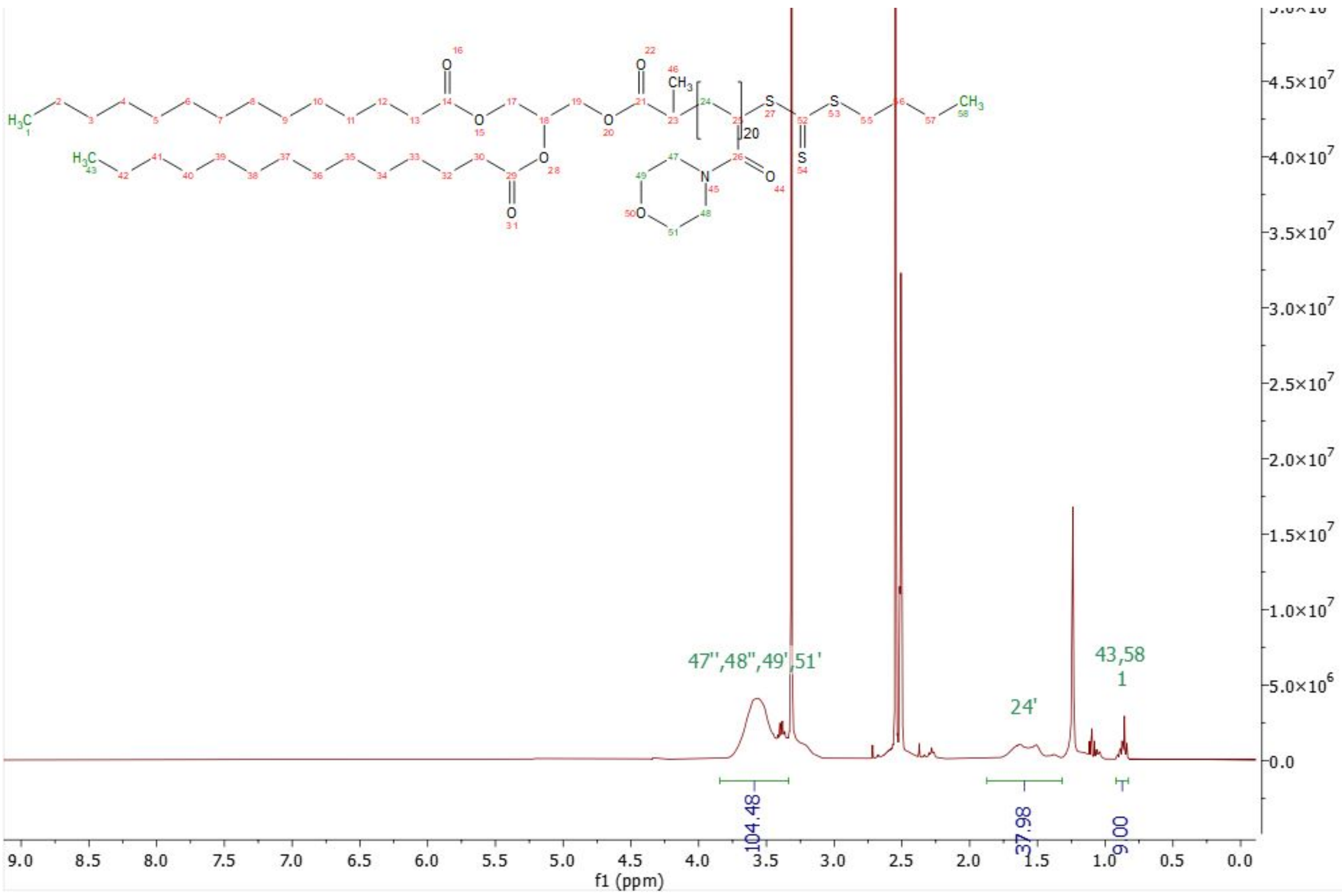


**Figure S35.** $^{1}$H NMR (d6-DMSO, 400 MHz) $NAM_{20}$-S

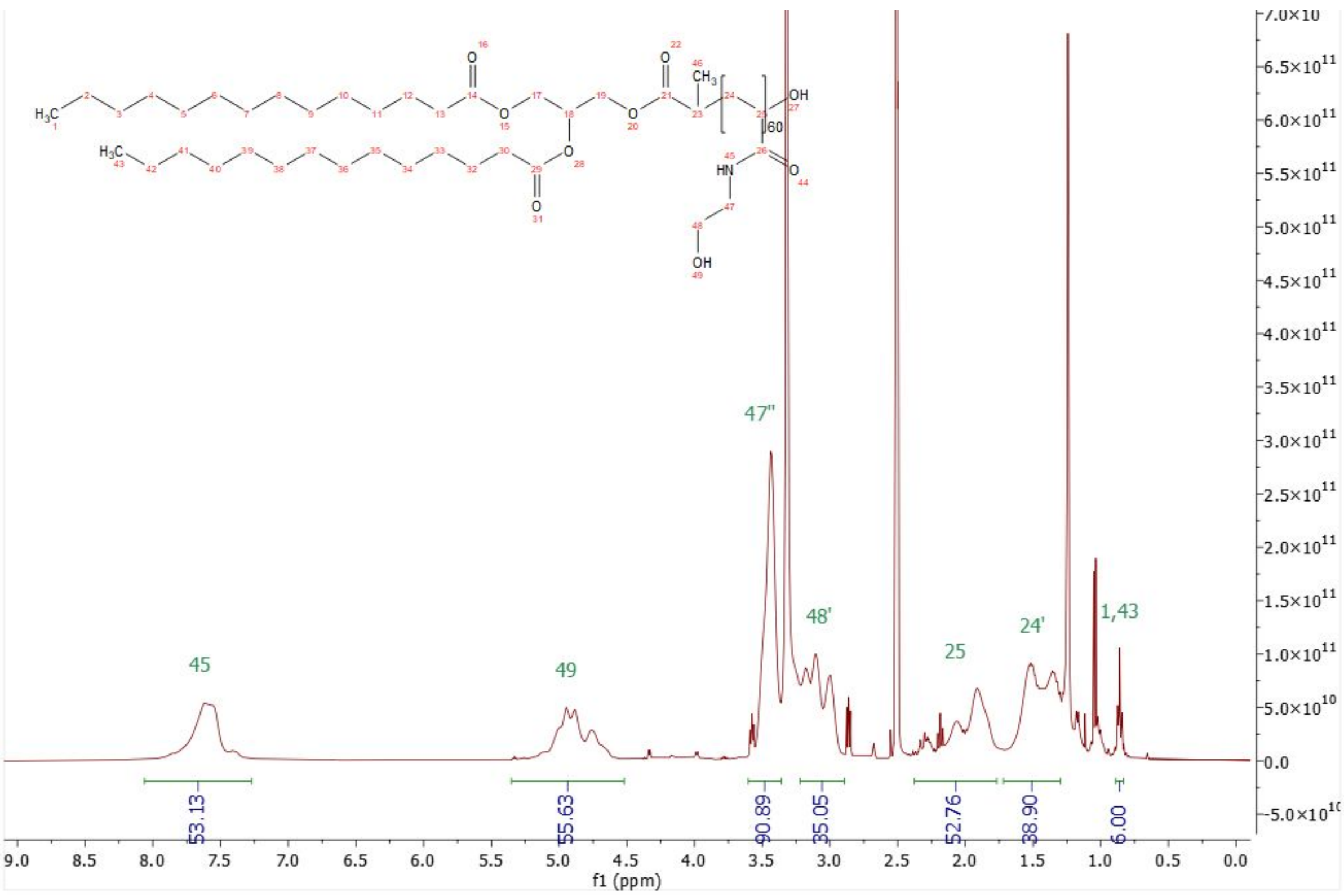


**Figure S36.** $^{1}$H NMR (d6-DMSO, 400 MHz) $HEA_{60}$-OH

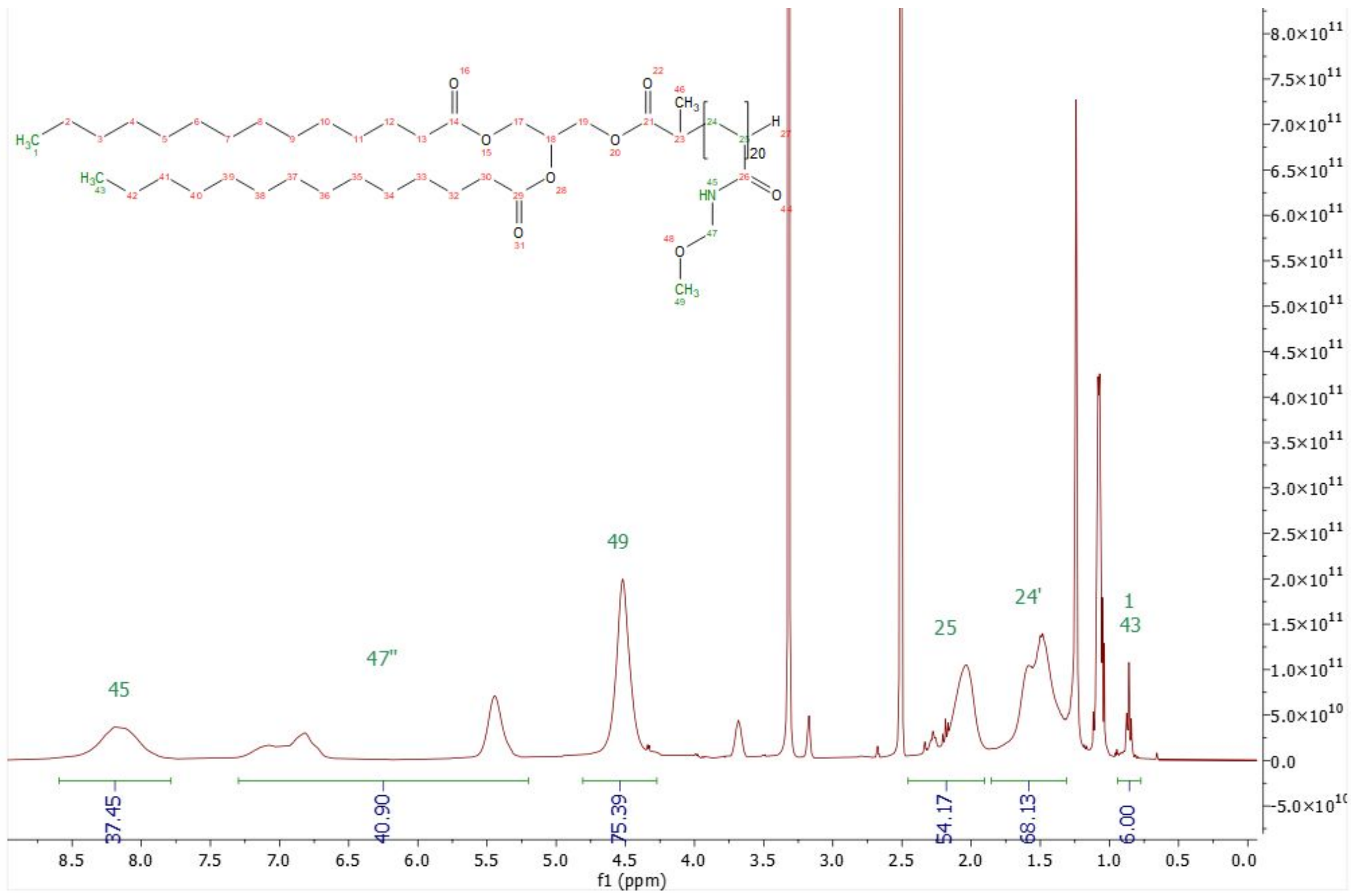


**Figure S37.** $^1$H NMR (d6-DMSO, 400 MHz) $MOMAM_{20}$-H

# References


(1) Padilla, M. S.; Shepherd, S. J.; Hanna, A. R.; Kurnik, M.; Zhang, X.; Chen, M.; Byrnes, J.; Joseph, R. A.; Yamagata, H. M.; Ricciardi, A. S.; Mrksich, K.; Issadore, D.; Gupta, K.; Mitchell, M. J. Elucidating lipid nanoparticle properties and structure through biophysical analyses. *Nat. Biotechnol.* **2025**, *44* (9), 1502–1515. https://doi.org/10.1038/s41587-025-02855-x

(2) Yang, Q.; Jacobs, T. M.; McCallen, J. D.; Moore, D. T.; Huckaby, J. T.; Edelstein, J. N.; Lai, S. K. Analysis of Pre-existing IgG and IgM Antibodies against Polyethylene Glycol (PEG) in the General Population. *Anal. Chem.* **2016**, *88* (23), 11804–11812. https://doi.org/10.1021/acs.analchem.6b03437

(3) Ju, Y.; Lee, W. S.; Pilkington, E. H.; Kelly, H. G.; Li, S.; Selva, K. J.; Wragg, K. M.; Subbarao, K.; Nguyen, T. H. O.; Rowntree, L. C.; Allen, L. F.; Bond, K.; Williamson, D. A.; Truong, N. P.; Plebanski, M.; Kedzierska, K.; Mahanty, S.; Chung, A. W.; Caruso, F.; Wheatley, A. K.; Juno, J. A.; Kent, S. J. Anti-PEG Antibodies Boosted in Humans by SARS-CoV-2 Lipid Nanoparticle mRNA Vaccine. *ACS Nano* **2022**, *16* (8), 11769–11780. https://doi.org/10.1021/acsnano.2c04543

(4) Chen, J.; Rizvi, A.; Patterson, J. P.; Hawker, C. J. Discrete Libraries of Amphiphilic Poly(ethylene glycol) Graft Copolymers: Synthesis, Assembly, and Bioactivity. *J. Am. Chem. Soc.* **2022**, *144* (42), 19466–19474. https://doi.org/10.1021/jacs.2c07859

(5) Kerr, A.; Hartlieb, M.; Sanchis, J.; Smith, T.; Perrier, S. Complex multiblock bottle-brush architectures by RAFT polymerization. *Chem. Commun.* **2017**, *53* (87), 11901–11904. https://doi.org/10.1039/c7cc07241d

(6) Gurnani, P.; Blakney, A. K.; Terracciano, R.; Petch, J. E.; Blok, A. J.; Bouton, C. R.; McKay, P. F.; Shattock, R. J.; Alexander, C. The In Vitro, Ex Vivo, and In Vivo Effect of Polymer Hydrophobicity on Charge-Reversible Vectors for Self-Amplifying RNA. *Biomacromolecules* **2020**, *21* (8), 3242–3253. https://doi.org/10.1021/acs.biomac.0c00698

(7) Zhang, J.; Tanaka, J.; Gurnani, P.; Wilson, P.; Hartlieb, M.; Perrier, S. Self-assembly and disassembly of stimuli responsive tadpole-like single chain nanoparticles using a switchable hydrophilic/hydrophobic boronic acid cross-linker. *Polym. Chem.* **2017**, *8* (28), 4079–4087. https://doi.org/10.1039/c7py00828g

(8) Walsh, D. J.; Su, E.; Guironnet, D. Catalytic synthesis of functionalized (polar and non-polar) polyolefin block copolymers. *Chem. Sci.* **2018**, *9* (20), 4703–4707. https://doi.org/10.1039/c8sc00450a

(9) Pfukwa, R.; Pound-Lana, G.; Klumperman, B. Facile End Group Modification of RAFT Made Polymers, by Radical Exchange with Hydrogen Peroxide. *Polym. Prepr. (Am. Chem. Soc., Div. Polym. Chem.)* **2008**, *49*, 118.

(10) Jesson, C. P.; Pearce, C. M.; Simon, H.; Werner, A.; Cunningham, V. J.; Lovett, J. R.; Smallridge, M. J.; Warren, N. J.; Armes, S. P. H2O2 Enables Convenient Removal of RAFT End-Groups from Block Copolymer Nano-Objects Prepared via Polymerization-Induced Self-Assembly in Water. *Macromolecules* **2017**, *50* (1), 182–191. https://doi.org/10.1021/acs.macromol.6b01963

(11) Chong, Y. K.; Moad, G.; Rizzardo, E.; Thang, S. H. Thiocarbonylthio End Group Removal from RAFT-Synthesized Polymers by Radical-Induced Reduction. *Macromolecules* **2007**, *40* (13), 4446–4455. https://doi.org/10.1021/ma062919u
